# Understanding tree failure—A systematic review and meta-analysis

**DOI:** 10.1371/journal.pone.0246805

**Published:** 2021-02-16

**Authors:** Marinus van Haaften, Yili Liu, Yuxin Wang, Yueyue Zhang, Cornelis Gardebroek, Wim Heijman, Miranda Meuwissen

**Affiliations:** 1 Agricultural Economics and Rural Policy Group, Wageningen University and Research, Wageningen, The Netherlands; 2 Inholland University of Applied Sciences, Domain Agri, Food and Life Sciences, Delft, The Netherlands; 3 Department of Economics, Czech University of Life Sciences, Prague, Czech Republic; 4 Business Economics Group, Wageningen University and Research, Wageningen, The Netherlands; Hainan University, CHINA

## Abstract

Recent research has indicated an increase in the likelihood and impact of tree failure. The potential for trees to fail relates to various biomechanical and physical factors. Strikingly, there seems to be an absence of tree risk assessment methods supported by observations, despite an increasing availability of variables and parameters measured by scientists, arborists and practitioners. Current urban tree risk assessments vary due to differences in experience, training, and personal opinions of assessors. This stresses the need for a more objective method to assess the hazardousness of urban trees. The aim of this study is to provide an overview of factors that influence tree failure including stem failure, root failure and branch failure. A systematic literature review according to the PRISMA guidelines has been performed in databases, supported by backward referencing: 161 articles were reviewed revealing 142 different factors which influenced tree failure. A meta-analysis of effect sizes and p-values was executed on those factors which were associated directly with any type of tree failure. Bayes Factor was calculated to assess the likelihood that the selected factors appear in case of tree failure. Publication bias was analysed visually by funnel plots and results by regression tests. The results provide evidence that the factors Height and Stem weight positively relate to stem failure, followed by Age, DBH, DBH squared times H, and Cubed DBH (DBH^3^) and Tree weight. Stem weight and Tree weight were found to relate positively to root failure. For branch failure no relating factors were found. We recommend that arborists collect further data on these factors. From this review it can further be concluded that there is no commonly shared understanding, model or function available that considers all factors which can explain the different types of tree failure. This complicates risk estimations that include the failure potential of urban trees.

## Introduction

In North-America, Latin America and the Caribbean, and Europe more than 70% of the people reside in urbanised regions [1]. In these urbanised regions trees increase liveability by adding climatological, financial, environmental, ornamental and social advantages. Trees contribute to the physical and mental health of citizens in many ways [2–5]. Environmental advantages include the capturing of airborne particulate matter and greenhouse gases, provision of habitats for urban fauna and flora and a reduction of rainwater runoff [5–7]. Urban trees influence the climate by mitigating urban heat island effects [7]. Besides environmental advantages, urban trees also provide positive externalities like financial benefits. Hedonic pricing methods indicate an increase in real estate property value due to urban trees [8–12]. The presence of urban trees affects multiple physical health benefits and reduces the stress levels for residents and patients [13, 14]. Despite the efforts of policymakers in expressing these benefits, sometimes people in urban regions express a negative view on urban trees [15, 16]. These are often rooted in issues of tree failure, which occasionally cause personal (injuries or death) or property damage [17, 18]. Therefore, policy makers and community managers have laid down the management of urban trees in tree risk management programs. In Europe and North-America many laws and regulations prescribe the liability for having such programs [19, 20]. These programs also deal with the assessment of tree risks, which are usually expressed to deal with ‘hazard or hazardous’ trees. A tree is hazardous when structural defects exist which can cause tree or branch failure and where this failure can cause personal or property damage [3, 21]. Failure arises when the tree or branch is unable to compensate the (lack of) forces the tree is exposed to, in the urban environment. In this study failure occurs when the systemic functioning fails, which includes overturning trees (maximum/critical overturning moment) apart from breaking stems and branches.

Despite maintenance activities of tree owners the likelihood of tree failure expressed in frequency and impact expressed in financial extent of property damage both appears to increase annually [22, 23]. Schmidlin [17] reported 407 wind-related tree failures causing death in the United States over the period 1997–2007. Another study by Dunster [22] reported an average annual increase of 39.9 online reported injuries and 23.4 online reported deaths by Google alerts for tree failure. From 2001–2010 one incident of tangible damage was estimated per 19,000 inhabitants in the Netherlands, with paid compensations up to € 49,296 [23]. Given climate change and the increase in extreme weather events (drought, windstorms) it is unlikely that the frequency of tree failure will decrease [24]. Apart from the frequency and impact of failure, the risk of tree failure is also affected by the failure potential of urban trees. Despite fluctuations in failure over time, for the majorities of urban trees the lifespan is limited. Smith, Dearborn and Hutyra [25] estimated the probability of an urban tree surviving 35 years to be 35.1 ± 8.6%, compared to the survival of a rural tree, i.e. 44.2 ± 4.5%. The failure potential includes tree characteristics and external factors that increase the risk of failure. Tree owners have tree risk assessments executed regularly, in order to prevent failure and estimate the failure potential and iterative necessary maintenance of the trees owned [26].

Since the ‘90s tree risk assessment methods to assess biomechanics, visual anomalies and the condition of trees have been developed [27–31]. There has been an increasing number of studies focussing on the applied procedures and technological devices to support these assessments. This increased simultaneously the number of variables and measured parameters and the variability of tree risk assessments. At the same time corresponding costs of urban tree inventory systems held by tree owners increased. Differences in tree risk assessments are also influenced by the individual evaluation of tree risk assessors. Field experts, i.e. arborists, who execute tree risk assessments use criteria and indicators combined with personal experience to identify and evaluate the hazardousness of urban trees [32]. Differences in educational level, personal biases and individual perceptions of risks lead to variability in the assessed hazardousness of urban trees and (possible) consequences [33, 34]. The execution of tree risk assessments often also involves a qualitative approach, the visual assessments (VTA), since biomechanical characteristics of trees cannot always be measured accurately from ground level (roots, branches) or only at high costs. Therefore, recent studies emphasize the importance of decreasing the number of variables [35–37]. Each study proposes a selection of variables promoted for tree risk assessments largely based on selections made by tree risk assessors (arborists, academics, policy makers).

Apart from tree assessments, several methods have been developed to identify tree failure, which can be broadly categorized into mechanistic models and statistical analyses. Recently, there has been some attention for machine learning techniques [38]. Gardiner et al. [39] reviewed the available mechanistic models e.g. HWIND [40] and Gales [41, 42]. Mechanical models are difficult to use, since these necessitate detailed information on every individual tree [43]. Statistical analysis often provides a better accuracy like in Kontogianni et al. [44] who discuss a Tree Stability Index based on the factors crown ratio (CR), crown asymmetry index (CAI), tree height (H) and other factors (slenderness index, crown fullness ratio, degree of spread, crown length, total tree size and crown roundness). Statistical models however include different levels of uncertainty in their models or in the factors included [38]. An increasing frequency of tree failure [22, 23], emphasizes the importance of an estimation of risks. Currently, there is no method which can predict urban tree failure accurately [45]. The increase in number of variables and parameters measured, based on technological developments and the variability in procedures and personal behaviour and opinions, necessitates an overview of factors that can be used to predict when trees fail and under which circumstances. The aim of this study is to investigate which factors relate to tree failure by conducting a systematic review and meta-analysis, which can contribute to the improvement of tree risk analysis in the future.

## Materials and methods

### Search strategy

The potential for tree or branch failure finds its origin in biological causes (fungi, decay, vigor), climatological conditions (wind, drought), mechanical (cracks, weak branch unions, leaning trees) and structural defects (branches with a sharp angle, lopsided branches) or site specific conditions [46, 47]. Therefore, it is expected that studies on tree failure are published in a broad range of scientific journals. To provide an overview of factors that influence urban tree failure a systematic review has been performed on articles published until July 25^th^ 2019. Systematic reviews are widely applied in social and medical sciences [48]. This systematic review has been conducted according to the guidelines of Preferred Reporting Items for Systematic Reviews and Meta-Analysis (PRISMA) [49]. The literature search in scholarly databases was executed by using the keywords ‘tree AND failure’, ‘tree AND stability, ‘tree AND hazard’, ‘branch AND failure’, ‘branch AND hazard’, ‘root AND failure’, ‘root AND stability’ and ‘root AND hazard’. Searches were rerun using additional search terms identified from keywords of studies that were considered relevant to this review (urban in combination with all previous keywords; stem AND failure; street trees AND failure). Databases include Science Direct, Scopus, Web of Science, Wiley Online Library, JSTOR, SpringerLink, Taylor & Francis and Google Scholar. Peer reviewed articles were selected and screened on the availability of full text.

Given the wide range of related fields and to avoid missing relevant studies [50], backward referencing was applied manually on references to identify additional relevant studies. Reference lists of primary papers, review articles and proceedings of symposia were crosschecked. Articles were selected that describe the influence of factors on the stability, failure or hazardousness of trees in urban regions or forestry. Additional manual searches were performed using the names of authors (forward and backward searching) known to have conducted research into tree failure. In the search three bibliographies were found, which were backward referenced [51–53], to identify relevant publications which were not included yet.

### Study selection and criteria

We collected and examined articles for reported effect sizes of factors related to tree failure and included studies that assessed single or multiple case studies. After conducting the search, titles and scope were screened to make sure the studies mentioned tree failure in relation to (urban) forestry or trees. Abstracts were assessed to check that these studies investigated tree failure. The articles were then checked in detail to verify the following inclusion criteria: 1) reporting of empirical data, i.e. qualitative studies were excluded, because of the absence of any measurements or associations of factors with tree failure; 2) investigation of tree failure in relation to a factor; 3) availability of full text; 4) provision of statistical data or an effect size, (articles with a biomechanical or statistical analysis do not always meet this criterion); 5) branch failure not due to abscission. This process was initially executed by the first author and then reviewed by the second author for root failure, by the third author for stem failure and by the fourth author for branch failure. In case of any differences the article was discussed and when for at least one of the researchers the study seemed relevant, the full text was assessed.

In a subsequent step, all included studies were assessed on their methodological quality, for the first time by the first author and for the second time by the second, third and fourth author for root, stem and branch failure, respectively. Any discrepancies between the first and second author were again assessed by both authors. This did not lead to further discrepancies. The methodological quality of the selected studies was assessed by criteria based on Pai et al. [54] and Wells et al. [55]. Seven criteria were adjusted for the field of tree failure, i.e. study design, analysis, description of the study population (forest, urban trees, open tree, orchard), data collection (prospective or retrospective), inclusion criteria, exclusion criteria, and full description of the relation to tree failure. The quality criteria including the scoring system are available in S1 Text.

### Data extraction and risk of bias

The selected studies were reviewed and the data extracted from the selected studies were tabulated using MS Excel for Windows. Data were registered at the level of individual species. The different studies were categorized by type of failure and factor. Furthermore detailed information was collected on author(s), year, country of the study, external influences, and sample size. Contrary to case studies, cohort studies and randomized trials were largely absent in the search results, which can influence study publication bias, since trees which did not fail while being exposed to the same circumstances are rarely mentioned in the selected studies. Data extraction was conducted twice, for the first time by the first author and for the second time the data was verified by the second, third and fourth author for root, stem and branch failure respectively. Data was extracted from the full texts of articles and supplementary materials. In case of discrepancies the data was assessed for a second time. Several studies contributed to more than one factor with multiple effect sizes, which can affect the independence of effect sizes. However, not considering them would have decreased the number of samples (i.e., number of available effect sizes). Plant nomenclature conforms to the International Plant Names Index (IPNI) by the Royal Botanic Gardens in Kew [56].

### Statistical methods

In this study correlations are used as effect sizes to explain heterogeneity of factors reported in literature, that are associated with tree failure for each species mentioned. Fisher’s z-transformation was applied to convert Pearson’s or Spearman’s correlation coefficients to obtain an approximately normally distributed value [57, 58]. We used t-tests to compare correlations between broadleaf and coniferous trees for each factor associated with tree failure. These t-tests between deciduous and coniferous trees resulted with and without transformed correlation coefficients in significant differences for the majority of factors. Therefore, the transformed effect sizes are used to calculate a mean transformed correlation weighted by the within-study variance based on the sample size [58], for deciduous and coniferous trees separately. A random-effects model with weighting of the different effect sizes was used. A separate analysis was performed for all effect sizes estimated for a specific factor. The averaged z values are used to test heterogeneity between the different samples examining whether all effect sizes are evaluating the same effect. Cochran’s Q-statistic is calculated to indicate the variance of the weighted squared deviations [59]. Heterogeneity is estimated with the I^2^-statistic and among-study variance τ^2^, to assess a proportion of the variance in effect sizes [60]. Heterogeneity was estimated in case two or more effect sizes for one factor and failure type were reported in literature [61].

We tested for publication bias by conducting the Egger regression test. Additionally, funnel plots have been drawn [62, 63]. In case of a small number of studies the publication bias is generally larger [64, 65]. The Cochrane handbook mentions a minimum of 10 studies or effect sizes [66]. Some studies set eight effect sizes as a rule of thumb for the minimum number for drawing a conclusion [67, 68]. When there are multiple studies which report effect sizes for a specific factor, the minimum included number of effect sizes in this study for analysing publication bias is eight. Since publication bias deals with the probability that significant effects are more often published than non-significant effects, both significant and non-significant effect sizes are reported.

Fisher’s combined probability test was used to determine a probable degree of heterogeneity under the assumption that a factor does not relate to tree failure [69]. The execution of the literature search revealed that many studies instead of an exact p-value just reported significance (< 0.05) or insignificance (> 0.05). Another consideration is that an insignificant effect does not cause the absence of an effect always to be most likely [70]. Therefore, all p-values (negative, positive, significant and not-significant) were included even when described as a disparity. In addition a Bayesian approach has been taken, since Bayesian factors provide a reliable alternative to problems of p-values. From the transformed r-value from each study for each factor a one-sample t-test has been conducted [71], in order to obtain a Bayes Factor for estimating the null effect. The Bayes Factors indicate the probability that the selected factors appear to have an effect on failure (H1) relative to having no effect at all (H0). The Bayes Factor has been calculated with JASP version 0.10.2 [72], all other calculations have been executed with Meta-Essentials [73].

Binary logistic regressions are used to explore the conditional probability that a factor or climate zone is associated with the presence of failure in the study populations [74, 75]. A smaller sample size lower than 50 can be sufficient if the aim of the analysis is to determine factors which are highly associated with an outcome [76]. The minimum sample size to process valid estimates and standard errors for binary logistic regression models has been estimated to be more than 20 [77], see also S4 Text in this regard. From the 161 studies included in the systematic review only the factors DBH, Height and Slenderness were reported often enough [78, 79], and 92 studies were included in this analysis. The identification of the climate zones of the locations reported in each study has been based on the Köppen climate classification [80]. Marginal effects are calculated to estimate the change in failure for different climate zones [81].

## Results

### Literature search

**Fig 1:**
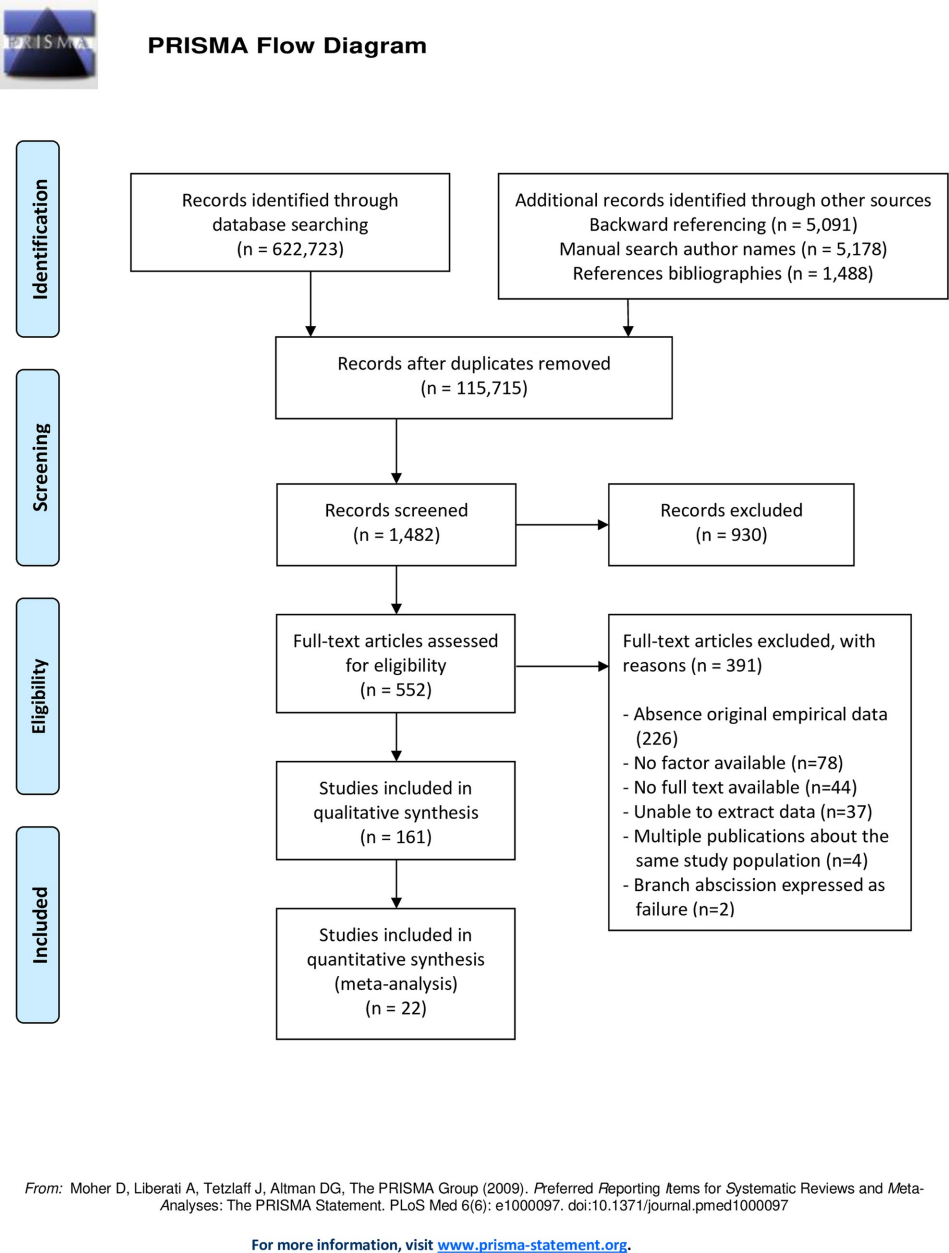
Flow chart of study selection process.

Studies discussing tree failure commonly discuss root failure, stem failure, branch failure, or a combination of two of the tree failure types. The literature search resulted in 161 observational studies as described in Fig 1. These 161 studies reported effect sizes which were associated with factors relating to one of the three failure types directly and indirectly. From the included studies direct and indirect effect sizes were collected, which are given in S1 Table for stem failure, S2 Table for root failure and S3 Table for branch failure. A separate overview of studies with significant and non-significant reported effect sizes per species is available upon request.

The total number of factors associated with failure was 93 factors for stem failure in 92 studies, 49 factors for root failure in 47 studies and 34 factors for branch failure in 22 studies. After elimination of duplicate factors the 161 observational studies reported a total of 142 different factors. The main characteristics of the studies included in the analysis are presented in S2 Text, the characteristics of all studies are given in S3 Text. The majority of the studies originated from North America (n = 82 studies), 50 studies were performed in Europe of which 22 in the UK, 13 studies were executed in Asia, 10 studies were conducted in Australia & New Zealand, 4 studies in Africa, and 2 studies in South America.

These 161 studies report correlations related to one of the three failure types and correlations between the 142 factors. Each factor related to one of the failure types was included in the meta-analysis when multiple studies reported this factor. The studies reporting associated factors with stem failure comprised 320 species, for root failure 102 species and for branch failure 32 species. Table 1 provides an overview of the distribution of species and studies per tree class and failure type.

**Table 1:** Distribution of species and studies per tree class and failure type.

	Broadleaf deciduous	Broadleaf evergreen	Conifer	Palm
**Stem failure (92 studies)**				
Total times species are observed	205	145	102	12
Total number of classes within studies	46	14	60	4
Average number of species/study/class	4.80	9.57	1.69	3.50
Total number of species/class	145	119	44	12
**Root failure (47 studies)**				
Total times species are observed	60	11	70	0
Total number of classes within studies	20	5	29	0
Average number of species/study/class	2.90	2.20	2.21	0
Total number of species/class	54	11	37	0
**Branch failure (22 studies)**				
Total times species are observed	35	4	5	1
Total number of classes within studies	20	1	4	1
Average number of species/study/class	2.15	4.00	1.25	1.00
Total number of species/class	22	4	5	1

In case of stem failure the majority of the studies investigated one or two coniferous species. All studies on deciduous trees included more than five different species. For root failure all studies on coniferous trees and deciduous trees reported characteristics on two or three species. Regarding branch failure each study commonly investigated one coniferous species and two deciduous species. The studies contained three deciduous gymnosperms, of which Ginkgo species are categorized as broadleaf deciduous, while Larix and Taxodium species are categorized as coniferous.

The different factors are affected by primary exogenous causes, being the first cause that did affect one or more of the factors. These factors are mentioned in S1 Table for stem failure, S2 Table for root failure and S3 Table for branch failure. An overview of the causes that affect these factors and its frequency are mentioned in Table 2.

**Table 2:** Overview of primary causes in the studies of the systematic review.

Cause	Total frequency	Stem failure	Root failure	Branch failure
Age (increasing)	4	4	0	0
Branch out of proportion to the crown	4	0	1	3
Civil engineering	1	1	0	0
Drought	5	1	4	0
Fungi, decay	12	10	1	1
Ice	4	1	0	3
Lack of pruning	3	0	0	3
Rockfall	1	1	0	0
Snow	2	0	0	2
Soil saturation	2	0	2	0
Urban environment	2	2	0	0
Vigour (decreasing)	4	4	0	0
Weak branch union	7	0	0	7
Weak or absent graft rootstock union	4	2	0	2
Wind	126	73	42	11

The majority of studies in the systematic review reported wind as the primary cause of tree failure, followed by fungi as primary cause. Studies which investigate stem failure also mentioned other causes as fungi and decay. Increasing tree age and decreasing vigour can occur at the same time. However, urban environmental circumstances cause trees to decline at a relatively young age. Other primary causes of tree failure mentioned are a deferred incompatibility of graft-rootstock union, civil engineering activities causing damage, drought resulting in a lower turgor pressure, the appearance of ice and rockfall. Apart from wind, studies which investigate root failure reported a shortage (drought) or abundance (soil saturation) of water supply, the presence of fungi and decay, and unbalance of the tree by branches which had grown disproportionally as other causes. Studies with the focus on branch failure reported additionally weak branch unions in case of bark inclusion, cracks or sharp bends, branches that have grown out of proportion behaving like codominant stems or lion tailing, weak or absent graft unions. Branches also fail because of gravitational loading by precipitation of ice or snow and because of the presence of fungi causing decay.

### Effect sizes (r) per factor

A total of 14 different factors have been analysed and interpreted, of which 12 relate to stem failure and 9 relate to root failure. No studies were found which reported correlations between any factor and branch failure. Most of these factors were reported in only a few studies and a comparison was executed in case of two or more reported effect sizes [61]. The total variation of each factor accounted for in the included studies, is represented by the value of the weighted mean correlation coefficients for stem failure and for root failure in Tables 3 and 5 respectively. In general the standard errors for root failure are larger than the standard errors for stem failure. This corresponds with a larger total sample size on average per study for stem failure contrary to root failure.

**Table 3:** Overview of transformed values between each factor and stem failure for related species and studies.

Factor^1^	Studies	Species	Range effect sizes	Weighted	Weighted	Standard error	Confidence interval
				average	mean		
				z-value	correlation		
Age	(Foster,1988), USA	Pinus resinosa sp.	0.68–0.84	1.0398	0.7778	0.072	LL: 0.74
		Pinus strobus L.					UL: 0.82
		Pinus sylvestris L.					
		Tsuga sp.					
		Conifer sp.					
Crown area	(Peltola et al., 2000), Finnland	Pinus sylvestris L.	0.788–0.973	1.6059	0.9226	0.111	LL: -1.00
		Picea abies L. Karst					UL: 1.00
Crown width	(Kane, 2014), USA	Acer saccharum.	0.8944–0.9644	1.3673	0.8781	0.177	LL: 0.53
	(Kane et al., 2014), USA	Quercus rubra L.					UL: 0.98
DBH	(Cucchi et al., 2004), France	Picea abies L. Karst.	0.68–0.972	14,299	0,8916	0,067	LL: 0.76
	(Hedden et al., 1995), USA	Pinus sylvestris L.					UL: 0.96
	(Moore, 2000), New Zealand	Pinus taeda L.					
	(Papesch et al., 1997), UK	Pinus radiata D.Don					
	(Peltola et al., 2000), Finnland						
DBH	(Cannon et al., 2015) USA	Betula sp.	0,8451–0.978	16,685	0,9314	0,091	LL: 0.90
	(Peltola et al., 2000), Finnland	Eschweilera spp					UL: 0.94
	(Peterson & Claassen, 2013), USA	Liriodendron tulipifera L.					
	(Ribeiro et al., 2016), Brazil	Populus fremontii Wats.					
		Quercus lobata Nee					
		Scleronema micranthum (Ducke) Ducke					
DBH^2^	(Cannon et al., 2015), USA	Liriodendron tulipifera L.	0.9295–0.9487	17,408	0,9403	0,036	LL: 0.91
	(Peterson & Claassen, 2013), USA	Populus fremontii Wats.					UL: 0.96
		Quercus lobata Nee					
DBH^2^H	(Cannon et al., 2015), USA	Chamaecyparis obtuse (Sieb. Et Zucc.) Endl.	0.670–0.9834	17,048	0,9360	0,058	LL: 0.85
	(Cucchi et al., 2004), France	Pinus taeda L.					UL: 0.98
	(Hedden et al., 1995), USA	Pinus pinaster Ait.					
	(Kamimura et al., 2012), Japan	Picea abies L. Karst					
	(Lundström et al., 2007), Switzerland	Pinus sylvestris L.					
	(Peltola et al., 2000), Finnland						
DBH^2^H	(Cannon et al., 2015), USA	Liriodendron tulipifera L.	0.9295–0.9731	19,626	0,9613	0,105	LL: 0.85
	(Peterson & Claassen, 2013), USA	Populus fremontii Wats.					UL: 0.99
		Quercus lobata Nee					
DBH^3^	(Cannon et al., 2015), USA	Pinus sylvestris L.	0,5745–0,9767	17,453	0,9408	0,048	LL: 0.82
	(Cucchi et al., 2004), France	Picea abies L. Karst					UL: 0.98
	(Fredericksen et al., 1993), USA	Pinus taeda L.					
	(Gardiner et al., 1997), UK	Pinus radiata D.Don					
	(Papesch et al., 1997), UK						
	(Peltola et al., 2000), Finnland						
DBH^3^	(Cannon et al., 2015), USA	Liriodendron tulipifera L.	0,9022–0,9985	22,261	0,9770	0,022	LL: 0.79
	(Peltola et al., 2000), Finnland	Betula sp.					UL: 0.98
	(Peterson & Claassen, 2013), USA	Populus fremontii Wats.					
		Quercus lobata Nee					
Height	(Cucchi et al., 2004), France	Pinus resinosa sp.	0.656–0.866	0.9908	0.7577	0.051	LL: 0.72
	(Foster, 1988), USA	Pinus strobus L.					UL: 0.80
	(Fredericksen et al., 1993), USA	Pinus sylvestris L.					
	(Hedden et al., 1995), USA	Tsuga sp.					
	(Papesch et al., 1997), UK	Conifer sp.					
	(Peltola et al., 2000), Finnland	Picea abies L. Karst					
		Pinus pinaster Ait.					
		Pinus taeda L.					
Height	(Cannon et al., 2015), USA	Acer rubrum L.	0.6557–0.976	12,544	0,8495	0,072	LL: 0.64
	(Foster, 1988), USA	Betula sp.					UL: 0.89
	(Peterson & Claassen, 2013), USA	Eschweilera spp					
	(Peltola et al., 2000), Finnland	Liriodendron tulipifera L.					
	(Ribeiro et al., 2016), Brazil	Populus fremontii Wats.					
		Quercus borealis					
		Quercus lobata Nee					
		Scleronema micranthum (Ducke) Ducke					
Stem volume	(Fredericksen et al., 1993), USA	Pinus radiata D.Don	0.8307–0.9695	18,037	0,9472	0,078	LL: 0.81
	(Lundström et al., 2007), Switzerland	Picea abies L. Karst					UL: 0.99
	(Papesch et al., 1997), UK						
Tree weight	(Achim et al., 2005), Canada	Abies balsamea L. Mill.	0.638–0.9798	16,388	0,9273	0,054	LL: 0.81
	(Cannon et al., 2015), USA	Chamaecyparis obtuse (Sieb. Et Zucc.) Endl.					UL: 0.98
	(Fredericksen et al., 1993), USA	Picea sitchensis Bong. Carr.					
	(Hedden et al., 1995), USA	Pinus taeda L.					
	(Kamimura et al., 2012), Japan	Picea abies L. Karst					
	(Lundström et al., 2007), Switzerland						
Stem weight	(Achim et al., 2003), UK	Picea abies L. Karst	0.721–0.9798	16,286	0,9259	0,036	LL: 0.88
	(Achim et al., 2004), Canada	Picea mariana Mill. BSP					UL: 0.95
	(Achim et al., 2005), Canada	Pinus banksiana Lamb.					
	(Cannon et al., 2015), USA	Pinus contorta Dougl. var. latifolia Engelm.					
	(Cucchi et al., 2004), France	Picea glauca (Moench) Voss					
	(Elie & Ruel, 2005), Canada	Abies balsamea L.					
	(Fraser, 1962), UK	Picea sitchensis Bong. Carr.					
	(Gardiner et al., 1997), UK						
	(Lundström et al., 2007), Switzerland						
	(Peltola et al., 2000), Finnland						
Stem weight	(Cannon et al., 2015), USA	Eschweilera spp.	.0.8268–0.9798	16,383	0,9272	0,130	LL: 0.23
	(Ribeiro et al., 2016), Brazil	Liriodendron tulipifera L.					UL: 0.99
		Scleronema micranthum (Ducke) Ducke					
Wind speed canopy top	(Hale et al,. 2012), UK	Larix decidua Mill.	0.8792–0.9854	18,407	0,9509	0,167	LL: 0.92
		Picea sitchensis Bong. Carr.					UL: 0.97

^1^ Factors: DBH = diameter breast height, DBH^2^ = diameter breast height squared, DBH^3^ = diameter breast height cubed, DBH^2^H = diameter breast height squared times height

The statistics on the factors associated with stem failure in Table 3 are presented in Table 4. The p-values indicating a significance for Cochrane’s Q differ in case of stem failure significantly for Diameter Breast Height (DBH) of coniferous trees and the related factors cubed Diameter Breast Height (DBH^3^) and squared Diameter Breast Height times Height (DBH^2^H) both of coniferous trees, Height of deciduous trees, Stem volume, Tree weight and Stem weight. The significance suggests the presence of heterogeneity especially with the larger sample sizes. Meaning that the correlations between these factors and stem failure among studies differ from each other for the different species. Table 4 shows that the non-significant values appear in cases of a small number of effect sizes for the factors Age, Crown area, Crown width and Wind speed canopy-top. In other words there is not enough statistical power to detect a variability that can explain heterogeneity. For the factor Height a non-significant Q statistic is shown with a corresponding relatively high number of effect sizes (see Table 4), appearing that there is absence of heterogeneity. Only the effect sizes related to crown width, were reported for deciduous trees. The reported effect sizes for all other factors come from coniferous trees.

**Table 4:** Meta-analytic statistics of effect sizes (r) from factors associated with stem failure.

Factor	Number of studies	Number of effect sizes	Total sample size	Q	P_Q_	I^2^ (%)	τ^2^	τ	p_F_	BF_10_
Age coniferous	1	6	195	1.40	0.924	0.00	0.00	0.00	0.000	33.616
Crown area coniferous	1	2	14	2.33	0.127	57.10	0.33	0.58	0.000	1.685
Crown width coniferous	2	4	35	2.04	0.361	1.94	0.00	0.05	0.000	5.331
DBH coniferous	7	8	224	34.12	0.000	82.41	0.19	0.43	0.000	8.688
DBH deciduous	5	6	125	1.24	0.871	0.00	0.00	0.00	0.000	32.144
DBH^2^ deciduous	2	3	93	0.44	0.801	0.00	0.00	0.00	0.000	14.073
DBH^2^H coniferous	6	8	300	65.08	0.000	89.24	0.25	0.50	0.000	29.055
DBH^2^H deciduous	2	3	93	4.28	0.118	53.26	0.04	0.21	0.000	12.238
DBH^3^ coniferous	6	7	436	170.39	0.000	96.48	0.57	0.76	0.000	30.817
DBH^3^ deciduous	3	4	96	4.57	0.102	56.26	0.05	0.22	0.000	28.816
Height coniferous	7	11	390	7.63	0.665	0.00	0.00	0.00	0.000	1297.727
Height deciduous	5	7	196	20.94	0.001	76.12	0.12	0.35	0.000	10.218
Stem volume coniferous	3	4	168	25.69	0.000	88.32	0.20	0.45	0.000	13.631
Tree weight (kg) coniferous	6	7	351	69.14	0.000	91.32	0.27	0.52	0.000	28.141
Stem weight (kg) coniferous	10	14	770	138.15	0.000	90.59	0.20	0.45	0.000	3764.541
Stem weight (kg) deciduous	2	3	62	11.27	0.004	82.26	0.31	0.56	0.000	1.831
Wind speed canopy-top coniferous	2	39	39	0.01	0.905	0.00	0.00	0.00	0.000	1.760

Q = Cochrane’s Q indicates variation around the average effect; P_Q_ = p-value indicating significance for Q; I^2^ = expresses consistency of effect sizes; τ and τ^2^ = estimates of heterogeneity; p_F_ displays the result of Fishers’ combined probability test; BF_10_ = represents Bayes Factor which indicates a favour for H_1_ over H_0_; n.a. = not available.

The statistics on factors associated with root failure in Table 5 are presented in Table 6. The p-values indicating a significance for Cochrane’s Q differ in case of root failure significantly for DBH^2^H of coniferous trees, Stem mass and Tree mass. This suggests the presence of heterogeneity, meaning that the correlations between these factors and root failure among studies differ from each other for the different species. Table 5 shows that the non-significant values for all other factors appear in cases of a small number of effect sizes, in other words that there is not enough statistical power to detect a variability which can explain heterogeneity. The reported effect sizes for two factors come from deciduous trees: DBH deciduous and DBH^2^H deciduous. The other factors originate from coniferous trees.

**Table 5:** Overview of transformed values between each factor and root failure for related species and studies.

Factor^1^	Studies	Species	Range effect sizes	Weighted	Weighted	Standard error	Confidence interval
				average	mean		
				z-value	correlation		
Angle of stem at maximum moment applied	(Peltola et al., 2000), Finnland	Pinus sylvestris L.	-0.406 –-0.513	0.4988	-0.4612	0.122	LL: -0.79
		Picea abies L. Karst					UL: 0.97
Crown area	(Peltola et al., 2000), Finnland	Pinus sylvestris L.	0.875–0.893	1.3953	0.8843	0.122	LL: -0.20
		Picea abies L. Karst					UL: 0.99
DBH	(Kane, 2014), USA	Betula spp	0.809–0.9644	1.4898	0.9033	0.104	LL: 0.22
	(Peltola et al., 2000), Finnland	Quercus rubra L.					UL: 0.99
	(Stokes et al., 2005), France	Fagus sylvatica L.					
DBH	(Papesch et al., 1997), UK	Pinus sylvestris L.	0.882–0.932	1.4884	0.903	0.087	LL: 0.80
	(Peltola et al., 2000), Finnland	Abies alba Mill.					UL: 0.96
	(Stokes et al., 2005), France	Picea abies L. Karst					
		Pinus radiate D.Don					
DBH^2^	(Lundström et al., 2007a), Switzerland	Picea abies L. Karst	0.8–0.938	1.2156	0.8383	0.092	LL: 0.82
	(Stokes et al., 2007), France	Abies alba Mill.					UL: 0.86
		Pinus sylvestris L.					
DBH^2^H	(Lundström et al., 2007a), Switzerland	Pinus sylvestris L.	0.938–0.983	1.5079	0.9066	0.12	LL: 0.77
	(Peltola et al., 2000), Finnland	Picea abies L. Karst					UL: 0.98
	(Stokes et al., 2005), France	Abies alba Mill					
DBH^2^H	(Peltola et al., 2000), Finnland	Fagus sylvatica L.	0.80–0.958	1.5074	0.9065	0.333	LL: -0.97
	(Stokes et al., 2005), France	Betula spp.					UL: 1.00
Height	(Peltola et al., 2000), Finnland	Pinus sylvestris L.	0.634–0.768	0.8868	0.7098	0.088	LL: 0.58
	(Smith, 1964), Canada	Picea abies L. Karst					UL: 0.82
		Picea mariana Mill. BSP					
Root soil plate depth	(Peltola et al., 2000), Finnland	Pinus sylvestris L.	0.495–0.693	0.6982	0.6032	0.122	LL: -0.84
		Picea abies L. Karst					UL: 0.99
Stem mass	(Lundström et al., 2007a), Switzerland	Picea abies L. Karst	0.100–0.866	1.5328	0.9109	0.063	LL: 0.88
	(Lundström et al., 2007b), Switzerland	Abies alba Mill					UL: 0.97
	(Peltola et al., 2000) Finnland	Pinus sylvestris L.					
Tree mass	(Lundström et al., 2007a), Switzerland	Picea abies L. Karst	0.872–0.990	1.7974	0.9465	0.074	LL: 0.85
	(Lundström et al., 2007b), Switzerland	Abies alba Mill					UL: 0.97
	(Stokes et al., 2005), France	Pinus sylvestris L.					

^1^ Factors: DBH = diameter breast height, DBH^2^ = diameter breast height squared, DBH^2^H = diameter breast height squared times height

**Table 6:** Meta-analytic statistics of effect sizes (r) from factors associated with root failure.

Factor	Number of studies	Number of effect sizes	Total sample size	Q	P_Q_	I^2^	τ	τ^2^	p_F_	BF_10_
Angle of stem at maximum moment applied coniferous	1	2	70	0.27	0.602	0.00	0.00	0.00	0.001	1.419
Crown area coniferous	1	2	70	0.10	0.751	0.00	0.00	0.00	0.001	2.257
DBH deciduous	3	3	19	1.75	0.417	3.07	0.00	0.03	0.000	1.516
DBH coniferous	3	4	134	2.06	0.356	0.00	0.00	0.00	0.000	4.268
DBH^2^ coniferous	2	6	182	0.34	0.953	89.84	0.27	0.52	0.000	4.447
DBH^2^H coniferous	3	7	192	39.37	0.000	0.00	0.00	0.00	0.000	2.810
DBH^2^H deciduous	2	2	12	0.56	0.455	0.00	0.00	0.00	0.000	0.924
Height coniferous	2	3	132	0.82	0.662	29.85	0.01	0.12	0.000	3.031
Root soil plate depth coniferous	1	2	70	1.43	0.232	82.05	0.14	0.38	0.001	1.210
Stem mass coniferous	3	8	256	33.42	0.000	74.51	0.09	0.30	0.000	19.864
Tree mass coniferous	3	7	188	15.96	0.003	0.00	0.00	0.00	0.000	13.443

Q = Cochrane’s Q indicates variation around the average effect; P_Q_ = p-value indicating significance for Q; I^2^ = expresses consistency of effect sizes; τ and τ^2^ = estimates of heterogeneity; p_F_ displays the result of Fishers’ combined probability test; BF_10_ = represents Bayes Factor which indicates a favour for H_1_ over H_0_.

The values above 50% for I^2^, occurring for the majority of factors for stem failure and the four factors for root failure, depict that more than half of the total heterogeneity originates from between-study variance which cannot be explained by sampling error exclusively. Meanwhile the studies are heterogeneous since the underlying true effect sizes vary (H_0_: τ^2^ = 0 is rejected in favour of H_1_: τ^2^ > 0).

All weighted mean correlation coefficients (see Tables 3 and 4) were significantly different from zero after applying Fishers’ combined probability test (see Tables 5 and 6). The Bayes Factors indicate the probability that these factors appear to have an effect on the failure types (stem/root) (H1) relative to having no effect at all (H0). Height and Stem weight of coniferous trees show an overwhelming preference for a very strong occurrence with stem failure. The factors Age (coniferous), Diameter Breast Height (DBH) (deciduous), DBH^2^ (deciduous), DBH^2^H (coniferous and deciduous), DBH^3^ (coniferous and deciduous), Height (deciduous), Stem volume (coniferous) and Tree weight (coniferous) indicate a strong evidence in favour of an occurrence with stem failure. The remaining factors appear to have a minor preference for an effect. With regard to root failure only the factors Stem mass and Tree mass show strong evidence in favour of an effect.

### Publication bias

**Fig 2:**
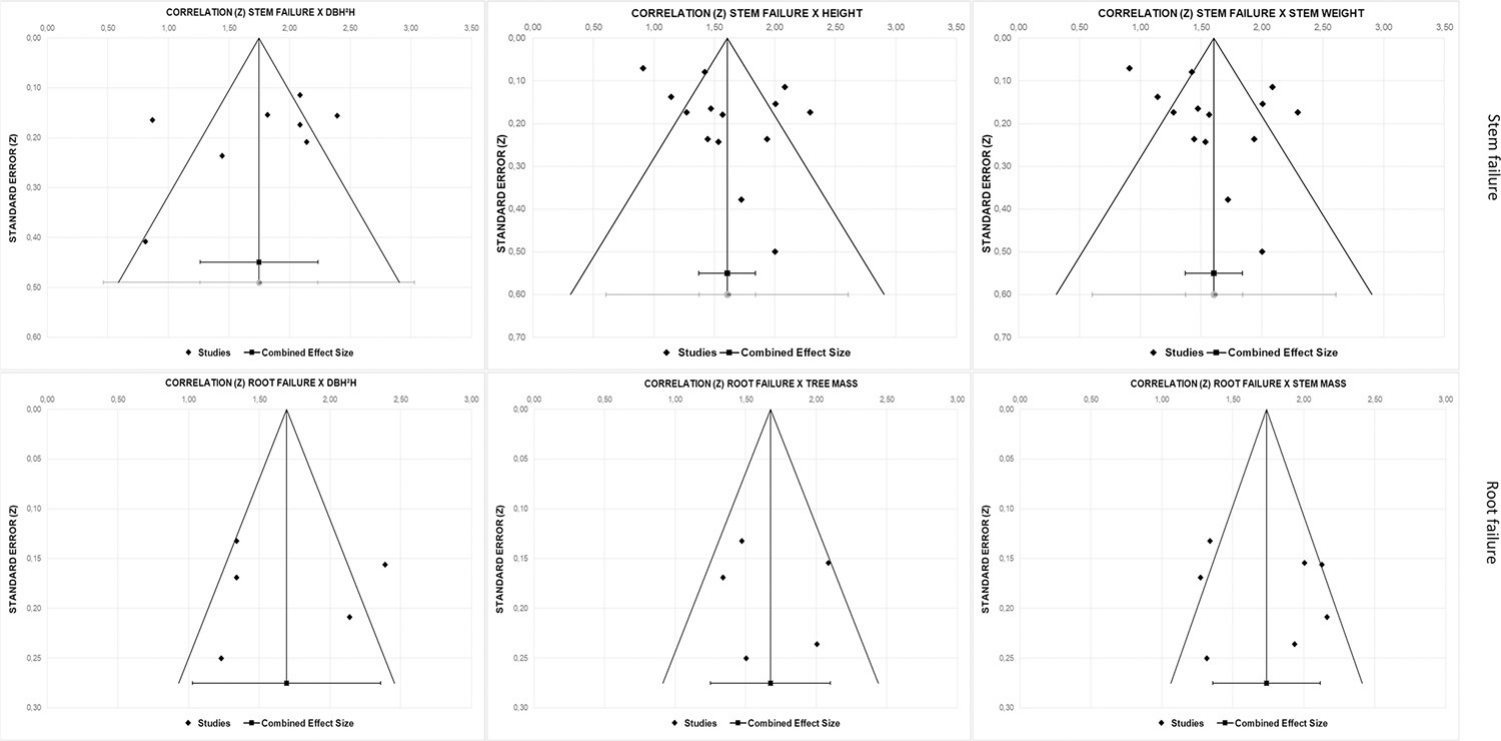
Funnel plot for factors associated with stem failure and root failure.

The number of studies and reported effect sizes on other factors than the ones mentioned in Table 7 are both small, making the effect of a publication bias relatively large. However, a visual examination of the funnel plots for factors with enough reported effect sizes did not indicate asymmetry in the reported effects (see Fig 2). Also, Eggers’ regression test provides a statistically non-significant estimate, suggesting absence of publication bias.

**Table 7:** Publication bias for factors associated with stem and root failure.

	Stem failure			Root failure		
Factor	DBH^2^H coniferous^1^	Height	Stem weight	DBH^2^H coniferous^1^	Stem mass	Tree mass
Eggers’ test	-1.69	-0.58	1.04	-0.31	0.03	0.22
p-value	0.14	0.57	0.32	0.78	0.97	0.84

^1^ DBH^2^H coniferous = diameter breast height squared times height of coniferous trees

### Logistic regression models

Individual binary logistic regressions were estimated on the presence of failure reported in the studies of the systematic review and the reported factors Diameter Breast Height (DBH), Height, Slenderness (Height/Diameter) and climate zone. The results showed a significant difference in the number of studies reporting failure for DBH, Height and climate zones. DBH, Height and climate zones do explain the variation in study populations from studies that report failure. See for detailed statistical information Tables 1 and 2 of S4 Text.

A full binary logistic regression model was fitted on failure reported in the included studies in the systematic review and climate regions, Height, DBH and Slenderness (see Table 3; S4 Text). The results indicate that DBH and Slenderness cannot be used worldwide in every climate region to predict tree failure. The marginal effects calculated from the regression results indicate small deviations of DBH for different climate zones (see Table 4; S4 Text).

## Discussion

In this discussion the outcome of the systematic review is discussed in comparison to literature after which the results from the meta-analysis follow, including strengths and limitations of this study. This discussion ends with the valorisation of the results for policy makers and industry and directions for future research.

Most of the reviewed studies originate from Anglo-Saxon countries (United States, United Kingdom and Australia). This can be explained from the facts that urban forestry as a research field has been present in those countries since the 1800s and that urban forestry was established as part of the culture and institutionalised by policies and legislation [82, 83]. The studies included have an observational character and refer to (multiple) case studies. The observational character of the majority of studies included, was limited to trees that failed and did not include data on the same factors for the trees which did not fail, which hampers the determination of a failure probability. In (urban) tree failure situations it is not always possible to extract randomized evidence from real-life effects. At best a comparison group can be formed of trees exposed to similar circumstances, expressing survival rates [84]. The observational study designs of the studies included, fit the efficacy of the reported outcomes [85]. The 161 factors initially found, depict a widely scattered set of explanatory factors which can predict tree failure. This is in line with other studies which report that there is currently no accurate method available to predict tree failure [45].

From the causes of tree failure reported in the studies of the systematic review, wind is predominantly mentioned to be the prior cause. This is in line with other study results which evaluate that windstorms were responsible for 53% of the total damage in forests over the period of 1850–2000 [24, 86]. The second most often reported cause of failure are fungi causing decay. Some fungi can cause a die off or break within a relatively short time (e.g. *Meripilus giganteus (Pers*.*) Karst*) [87].

In line with other research, regression results show significant evidence for height to be a predicting factor for tree failure in all climate zones [89]. However, there is many data described in professional journals, magazines and reports which are not reported in scientific journals from which slenderness could be calculated. Including this data in the analysis could influence findings of factors like DBH and Slenderness.

The meta-analysis presents an analysis on 14 different factors which are directly significantly associated with stem or root failure. Stem failure and root failure are the most common types of tree failure [29, 88]. The analysis on the Bayes Factor clearly indicates that the data of some of the factors is present in case of stem or root failure, confirming the effects reported in the corresponding literature. No effect sizes were found that are directly related to branch failure. Only one study reported correlations of factors related to breaking stress of branch failure, which is not enough for a statistical comparison in a meta-analysis. The limited results in the literature can be explained by the relatively recent attention for this failure type.

The available effect sizes for stem failure and root failure predominantly originate from coniferous trees, while the majority of trees in urban conditions are broadleaf deciduous or broadleaf evergreen trees. Although differences in growth between urban trees and rural trees decline with increasing age, in the temperate climate zone growth of urban trees has been slower than of rural trees [89]. This could have affected the strength of the effect sizes (correlations) reported in the included studies and influenced heterogeneity. This study also shows that the associated effect sizes are not always observed consistently across studies, even after categorizing the effect sizes for coniferous or deciduous. Other studies indicated that predictive relationships for tree failure of conifers do not apply to open-grown deciduous trees for a variety of reasons. The crown structure and dynamic properties (e.g. natural frequency, damping ratio) of broadleaf trees contrasts with coniferous trees [90, 91]. The anatomical structure of the wood differs between coniferous and broadleaf trees, providing both with different stem dynamics [92, 93]. The presence of heterogeneity can be explained by four different influences: (1) The invisibility of the presence of internal defects before a tree actually fails [94, 95], (2) the biomechanical properties of green wood which can differ even for individual trees of the same species [96–98], (3) the growing conditions influencing morphological differences between urban trees in different climate zones [99, 100] and (4) the strength of the correlation between a factor and any failure type which is influenced by its habitat [101]. A complicating issue is that the presence of external causes of tree failure like decay can contribute largely to individual differences between trees. Ribeiro et al. [79] showed that in trees (*Eperua glabriflora (Ducke) Cowan*) with a similar DBH, the presence of decay influenced the variation of stem fresh wood density both radially (1.02 g cm^-3^ at 38 cm in DBH and 0.594 g cm^-3^ at 35.3 cm in DBH) and axially.

Apart from the factors for which effect sizes were found there are also other factors mentioned in literature for which effect sizes were not found: structural defects [102, 103], condition or vigour [104, 105], maintenance history [106, 107], absence or bad execution of pruning [78, 105], civil engineering activities [104, 108], compacted soils and low nutrient levels [109, 110], site characteristics [110, 111], lack of water supply [48, 112] nursery circumstances [18, 113], planting season [114, 115], and a vast body of literature on pests and diseases [116, 117]. Absence of effect sizes (r) is partly understandable since the data collected in these studies cannot always be measured or assumed to be uniform. The limited number of studies reporting data on the level of individual species for individual factors, contribute to the absence of effect sizes as well.

To provide a more accurate estimate all effect sizes, significant, negative and positive have been included. No non-significant effect sizes were found for the factors included in the meta-analysis, except for DBH and stem failure. Assner and Goldstein [118] investigated relationships between DBH and stem failure with data collected from five different broadleaf species and found a non-significant negative effect size of -0.15 (p > 0.05) between DBH and stem failure. In their discussion they mentioned that this is not in line with results from other studies they found. In the search results of this study, this was the only non-significant effect size found.

The low numbers of effect sizes in combination with heterogeneity, limit the possibility of generalizable conclusions for stem or root failure. Since studies from non-English journals are limited in this study, the findings of this study may not hold globally. The search strategy of backward referencing can result in citation bias, because of the many possible motivations there are for citing an article [119].—The use of other keyword combinations relating to the results mentioned in Fig 1 (e.g. causes AND tilt AND diseases, causes AND fungi AND failure) might have provided a higher yield of included studies.

The wide range of reported factors which are associated with stem, root or branch failure, complicates the determination of critical values for these factors and the prediction of failure. The number of studies which show effect sizes of factors that are directly associated with stem failure is often too small to estimate whether the published data of those studies emerge in case of stem or root failure. An increase in the number of studies reporting effect sizes which can be associated directly to any failure type (stem/root/branch), is indispensable for future investigations on tree failure. It is also important to report effect sizes both for trees that failed and trees that did not fail. As can be seen from this study effect sizes are not always reported very precisely. Likewise corresponding p-values are often reported as a disparity (> / < 0.05) instead of a more exact figure. In case of a comparison of effect sizes and/or corresponding p-values, the more precise these are the more accurate the interpretation of a comparison can be [120].

A growing body of literature especially for broadleaf trees, may also allow further elucidation of tree risk assessment methods. Several studies show that pruning activities which contribute to a crown length that is proportionally formed with tree height or which reduce the slenderness factor improve tree stability and reduce failure [44, 108]. Also, sufficient water supply decreases failure by diminishing the wind load [103, 108] and summer branch drop respectively [88]. The industry of arborists often applies tree risk assessment methods based on a combination of methods which are developed for assessing the structure of a tree [121, 122]. Currently, tree risk assessment methods lack a body of observational literature that can substantiate these tree risk assessment methods quantitatively. This study emphasizes the relevance of collecting data on research done by arborists and of recording data on trees by tree owners (e.g. municipalities) for the factors Height, Stem weight and Tree weight. Additionally, this study provides quantitative support for factors arborists collect information about during an assessment, e.g. the Diameter at Breast Height, Height and Age of a tree. This does not mean that other factors should not be investigated, recorded and analyzed. Other observational studies might reveal more relevant factors.

## Conclusion

The results of this study provide a useful basis to explore differences in biological (e.g. fungi), climatological (e.g. climate zones) and mechanical (e.g. total tree height / DBH, crown dimensions) causes of tree failure among different study sites as reported by each individual study.

In the literature there is no commonly shared understanding, model or function available which expresses the joint distribution of factors explaining tree failure. The widely scattered range of factors together with the low number of studies and sample sizes reported for each factor complicates estimations of the failure potential of species based on tree characteristics and external factors.

The results provide evidence that the factors Height and Stem weight positively relate to stem failure, followed by Age, Diameter Breast Height, Diameter Breast Height squared times Height, and Cubed Diameter Breast Height (DBH^3^) and Tree weight. For stem failure there is heterogeneity between different effect sizes (i.e. species) for the factors Diameter Breast Height and the related factors Diameter Breast Height^3^ and Diameter Breast Height^2^ times Height, Stem volume, Tree weight and Stem weight for coniferous trees. For the factors Age, Crown area, Crown width and Stem weight for deciduous trees, the small number of effect sizes makes it impossible to detect heterogeneity. For the factor Height there is absence of heterogeneity between effect sizes.

For the factors Stem weight and Tree weight this study provides evidence that these positively relate to root failure. For root failure the results indicate the presence of heterogeneity between different effect sizes (i.e. species) for Diameter Breast Height^2^ times Height of coniferous trees, Stem mass and Tree mass. For the factors Angle of stem at maximum moment applied, Crown area, Diameter Breast Height, Diameter Breast Height^2^, Diameter Breast Height^2^ times Height deciduous trees, Height, and Root plate soil depth, the small number of effect sizes makes it impossible to determine any heterogeneity.

## Supporting information

S1 Checklist PRISMA 2009 checklist. (DOC)

S1 Table Overview of effect sizes for stem failure. (DOCX)

S2 Table Overview of effect sizes for root failure. (DOCX)

S3 Table Overview of effect sizes for branch failure. (DOCX)

S1 Text Quality criteria and assessment. (DOCX)

S2 Text Overview of study characteristics included in the meta-analysis. (DOCX)

S3 Text Overview of study characteristics included in the systematic review. (DOCX)

S4 Text Detailed results of data analysis for binary logistic regressions. (DOCX)
