# Supplementary material for: Understanding tree failure—A systematic review and meta-analysis
Source: PLoS One. 2021 Feb 16;16(2):e0246805. doi: 10.1371/journal.pone.0246805 (PMC7886209; doi:10.1371/journal.pone.0246805)
Supplement: S1 Table — (DOCX) [file pone.0246805.s002.docx]

S1 Table: Overview of all effect sizes (correlations) mentioned in studies of stem failure (Note: factors can be labelled horizontal and vertical)

| Stem failure | | Variables^5^ | Age | Anchorage strength (Maximum M (Nm)) | Crown depth | Crown mass | Crown length | Crown loss | Crown ratio | Damping ratio °ξ | Degree of spread | DBH | Diameter Stump | Height | Leaf retention strength | Maximum decli-nation midstem | MOE | MOR | Natural frequency ƒn | Resistance to flexing | Root failure | root plate depth (height) | root plate volume | Stem failure | Stem mass | Stem volume | Stem weight | tree center of mass | Turning moment (kN m) | vertical radius root plate (width) | Wood density/Specific gravity |
| --- | --- | --- | --- | --- | --- | --- | --- | --- | --- | --- | --- | --- | --- | --- | --- | --- | --- | --- | --- | --- | --- | --- | --- | --- | --- | --- | --- | --- | --- | --- | --- |
| Author (year) | Species |  |  |  |  |  |  |  |  |  |  |  |  |  |  |  |  |  |  |  |  |  |  |  |  |  |  |  |  |  |  |
| (Veselkin et al., 2015), Russia | *Pinus sylvestris L.* | Age | – |  |  |  |  |  |  |  |  | 0.42  (0.011) |  | 0.33  (0.048) |  |  |  |  |  |  |  |  |  |  |  |  |  |  |  |  |  |
| (Ribeiro et al., 2016), Brazil | *Eschweilera spp.* | Above ground biomass (kg) |  |  |  | 0.9529  (< 0.05) |  |  |  |  |  | 0.9433  (< 0.05) | 0.9011  (< 0.05) | 0.8249  (< 0.05) |  |  |  |  |  |  |  |  |  |  | 0.9430  (< 0.05) | 0.9275  (< 0.05) |  | 0.7997  (< 0.05) | 0.8681  (< 0.05) |  | 0.0149  (< 0.05) |
| (Ribeiro et al., 2016), Brazil | *Scleronema micranthum (Ducke) Ducke* | Above ground biomass (kg) |  |  |  | 0.9638  (< 0.05) |  |  |  |  |  | 0.9756  (< 0.05) | 0.9394  (< 0.05) | 0.8735  (< 0.05) |  |  |  |  |  |  |  |  |  |  | 0.9908  (< 0.05) | 0.9871  (< 0.05) |  | 0.8383  (< 0.05) | 0.9078  (< 0.05) |  | 0.1854  (< 0.05) |
| (Peltola et al., 2000), Finnland | *Betula spp* | Angle of stem at maximum moment applied |  |  |  |  |  |  |  |  |  |  |  |  |  |  |  |  |  |  | -0.454  (> 0.05) |  |  | -0.531  (< 0.05) |  |  |  |  |  |  |  |
| (Peltola et al., 2000), Finnland | *Picea abies L. Karst* | Angle of stem at maximum moment applied |  |  |  |  |  |  |  |  |  |  |  |  |  |  |  |  |  |  | -0.406  (< 0.05) |  |  | -0.414  (> 0.05) |  |  |  |  |  |  |  |
| (Peltola et al., 2000), Finnland | *Pinus sylvestris L..* | Angle of stem at maximum moment applied |  |  |  |  |  |  |  |  |  |  |  |  |  |  |  |  |  |  | -0.513  (< 0.02) |  |  | 0.181  (> 0.05) |  |  |  |  |  |  |  |
| (Papesch et al., 1997), UK | *Pinus radiata D. Don* | Angle stem deflection at maximum applied bending moment |  |  |  |  |  |  |  |  |  | 0.5683  (< 0.05) |  | 0.7204  (< 0.05) |  |  |  |  |  |  |  |  |  |  |  | 0.6317  (< 0.05) |  |  |  |  |  |
| (Sánchez-Medina et al., 2017), Argentina | *Tabebuia impetiginosa, Tipuana tipu, Citrus × aurantium* | Canopy area |  |  |  |  |  |  |  |  |  | 0.7095  (<0.05) |  | 0.5431  (< 0.05) |  |  |  |  |  |  |  |  |  |  |  |  |  |  |  |  |  |
| (Hedden, et al., 1995), USA | *Pinus taeda L.* | Center of gravity |  |  |  |  |  |  |  |  |  |  |  |  |  |  |  |  |  |  |  |  |  | 0.79  (<0.0001) |  |  |  |  |  |  |  |
| (Kane & Smiley, 2006), USA | *Acer rubrum L.* | Centre pressure of height |  |  |  |  | 0.83  (< 0.05) |  |  |  |  |  |  | 0.87  (<0.05) |  |  |  |  |  |  |  |  |  |  |  |  |  |  |  |  |  |
| (Peltola et al., 2000), Finnland | *Betula spp* | Crown area |  |  |  |  |  |  |  |  |  |  |  |  |  |  |  |  |  |  | 0.827  (< 0.05) |  |  | 0.801  (< 0.01) |  |  |  |  |  |  |  |
| (Peltola et al., 2000), FInnland | *Picea abies L. Karst* | Crown area |  |  |  |  |  |  |  |  |  |  |  |  |  |  |  |  |  |  | 0.893  (< 0.01) |  |  | 0.973  (< 0.01) |  |  |  |  |  |  |  |
| (Peltola et al., 2000), FInland | *Pinus sylvestris L..* | Crown area |  |  |  |  |  |  |  |  |  |  |  |  |  |  |  |  |  |  | 0.875  (< 0.01) |  |  | 0.788  (< 0.01) |  |  |  |  |  |  |  |
| (Kontogianni et al. 2011), Greece | *Platanus orientalis; Celtis australis; Albizia julibrissin; Populus x euroamericana cv. ‘I-45/51’* | Crown asymmetry + Crown ratio + Height |  |  |  |  |  |  | 0.69  (< 0.05) |  | 0.37  (< 0.05) |  |  | 0.63  (< 0.05) |  |  |  |  |  |  |  |  |  |  |  |  |  |  |  |  |  |
| (Cucchi et al., 2004), France | *Pinus pinaster Ait.* | Crown biomass (kg) |  |  |  |  |  |  |  |  |  |  |  |  |  |  |  |  |  |  |  |  |  |  |  |  |  |  | 0.95  (<0.001) |  |  |
| (Hedden et al., 1995), USA | *Pinus taeda L.* | Crown green weight |  |  |  |  |  |  |  |  |  |  |  |  |  |  |  |  |  |  |  |  |  | 0.74  (<0.0001) |  |  |  |  |  |  |  |
| (Ribeiro et al., 2016), Brazil | *Eschweilera spp.* | Crown mass (kg) |  |  |  |  |  |  |  |  |  |  | 0.7802  (< 0.05) |  |  |  |  |  |  |  |  |  |  |  | 0.7977  (< 0.05) |  |  |  | 0.8183  (< 0.05) |  |  |
| (Ribeiro et al., 2016), Brazil | *Scleronema micranthum (Ducke) Ducke* | Crown mass (kg) |  |  |  |  |  |  |  |  |  |  | 0.8782  (< 0.05) |  |  |  |  |  |  |  |  |  |  |  | 0.9188  (< 0.05) |  |  |  | 0.8862  (< 0.05) |  |  |
| (Gilmore, 2000), USA | *Larix laricina (Du Roi) K. Koch, Larix decidua Mill., Larix decidua x Larix leptolepis (Sieb. and Zucc.)* | Crown length |  |  |  |  |  |  |  |  |  | 0,8933  (< 0.05) |  | 0,9935  (< 0.05) |  |  |  |  |  |  |  |  |  |  |  |  |  |  |  |  |  |
| (Francis, 2000), USA | 24 different species^2^ | Crown loss |  |  |  |  |  |  |  |  |  |  |  |  | -0.232  (0.275) |  |  |  |  | -0.134  (0.534) |  |  |  | 0.546  (0.006) |  |  |  |  |  |  | -0.294 (0.164) |
| (Gilmore, 2000), USA | *Larix laricina (Du Roi) K. Koch, Larix decidua Mill., Larix decidua x Larix leptolepis (Sieb. and Zucc.)* | Crown radius |  |  |  |  | 0,5848  (< 0.05) |  |  |  |  | 0,6261  (< 0.05) |  | 0,5950  (< 0.05) |  |  |  |  |  |  |  |  |  |  |  |  |  |  |  |  |  |
| (Moore, 2000), New Zealand | *Pinus radiata* | Crown volume |  |  |  |  |  |  |  |  |  |  |  |  |  |  |  |  |  |  |  |  |  |  |  |  |  |  | 0.92  (<0.001) |  |  |
| (Hedden et al., 1995), USA | *Pinus taeda L.* | Crown weight : stem weight |  |  |  |  |  |  |  |  |  |  |  |  |  |  |  |  |  |  |  |  |  | 0.54  (< 0.0004) |  |  |  |  |  |  |  |
| (Kane et al., 2014), USA | *Acer saccharum Marsh.* | Crown width |  |  |  |  |  |  |  | 0.78  (< 0.05) |  |  |  |  |  |  |  |  |  |  |  |  |  |  |  |  |  |  |  |  |  |
| ([King, 1986](#_ENREF_1)), USA | *Acer saccharum Marsh.* | Crown width |  |  |  |  |  |  |  |  |  | 0.61  (< 0.01) |  |  |  |  |  |  |  |  |  |  |  |  |  |  |  |  |  |  |  |
| (Kane, 2014), USA | *Quercus rubra L.* | Crown width trunk failure |  |  |  |  |  |  |  |  |  |  |  |  |  |  | 0.54  (< 0.05) |  |  |  |  |  |  |  |  |  |  |  | 0.89  (< 0.05) |  |  |
| (Kane, 2014), USA | *Quercus rubra L.* | Crown width uprooted trees |  |  |  |  |  |  |  |  |  |  |  |  |  |  |  |  |  |  |  |  |  |  |  |  |  |  | 0.96  (< 0.05) |  |  |
| (Veselkin et al., 2015), Russia | *Pinus sylvestris L.* | Damage index | -0.17  (0.316) |  |  |  |  |  |  |  |  | -0.36 ( 0.028) |  | -0.01 (0.973) |  |  |  |  |  |  |  |  |  |  |  |  |  |  |  |  |  |
| (Hedden et al., 1995), USA | *Pinus taeda L.* | Deflection at midstem |  |  |  |  |  |  |  |  |  |  |  |  |  |  |  |  |  |  |  |  |  | -0.40  (0.0113) |  |  |  |  |  |  |  |
| (Asner & Goldstein, 1997), USA | 5 species¹ | DBH |  |  |  |  |  |  |  |  |  |  |  |  |  |  |  |  |  |  | 0.15  (> 0.15) |  |  | -0.15  (> 0.05) |  |  |  |  |  |  |  |
| (Achim et al., 2005), Canada | *Abies balsamea L. Mill.* | DBH |  |  | 0.80  (< 0.001) |  | 0.75  (< 0.001) |  |  |  |  |  |  |  |  |  |  |  |  |  |  |  |  |  |  |  |  |  |  |  |  |
| (Kamimura et al., 2012), Japan | *Chamaecyparis obtuse*  *(Sieb. Et Zucc.) Endl.* | DBH |  |  |  |  |  |  |  |  |  |  |  |  |  |  |  |  |  |  |  |  |  |  |  |  |  |  | 0.710  (0.032) |  |  |
| (Cannon et al., 2015), USA | *Liriodendron tulipifera L.* | DBH |  |  |  |  |  |  |  |  |  |  |  |  |  |  |  |  |  |  |  |  |  |  |  |  |  |  | 0,9220  (< 0.05) |  |  |
| (Cucchi et al., 2004), France | *Pinus pinaster Ait.* | DBH (cm) |  |  |  |  |  |  |  |  |  |  |  |  |  |  |  |  |  |  |  |  |  |  |  |  |  |  | 0,9487 (<0.001) |  |  |
| (Hedden et al., 1995), USA | *Pinus taeda L.* | DBH (cm) |  |  |  |  |  |  |  |  |  |  |  |  |  |  |  |  |  |  |  |  |  | 0.68  (< 0.0001) |  |  |  |  |  |  |  |
| (Lundström et al., 2007), Switzerland | *Picea abies L. Karst.* | DBH (cm) |  | 0.9695  (< 0.001) |  |  |  |  |  |  |  |  |  |  |  |  |  |  |  |  |  | 0.49  (< 0.001) | 0.52  (<0.001) |  |  |  |  |  |  | 0.55  (<0.001) |  |
| (Papesch et al., 1997), UK | *Pinus radiata D. Don* | DBH (cm) |  |  |  |  |  |  |  |  |  |  |  |  |  |  |  |  |  |  |  |  |  |  |  |  |  |  | 0.8820  (< 0.01) |  |  |
| (Peltola et al., 2000), Finnland | *Betula spp* | DBH (cm) |  |  |  |  |  |  |  |  |  |  |  |  |  |  |  |  |  |  | 0.809  (< 0.05) |  |  | 0.978  (< 0.01) |  |  |  |  |  |  |  |
| (Peltola et al., 2000), Finnland | *Picea abies L. Karst* | DBH (cm) |  |  |  |  |  |  |  |  |  |  |  |  |  |  |  |  |  |  | 0.896  (< 0.01) |  |  | 0.889  (< 0.01) |  |  |  |  |  |  |  |
| (Peltola et al., 2000), Finnland | *Pinus sylvestris L..* | DBH (cm) |  |  |  |  |  |  |  |  |  |  |  |  |  |  |  |  |  |  | 0.953  (< 0.01) |  |  | 0.913  (< 0.01) |  |  |  |  |  |  |  |
| (Peterson & Claassen, 2013), USA | *Populus fremontii Wats.* | DBH (cm) |  |  |  |  |  |  |  |  |  |  |  |  |  |  |  |  |  |  |  |  |  |  |  |  |  |  | 0,9116  (<0.0001) |  |  |
| (Peterson & Claassen, 2013), USA | *Quercus lobata Nee* | DBH (cm) |  |  |  |  |  |  |  |  |  |  |  |  |  |  |  |  |  |  |  |  |  |  |  |  |  |  | 0,9295  (<0.0001) |  |  |
| (Ribeiro et al., 2016), Brazil | *Eschweilera spp* | DBH (cm) |  |  |  | 0.8614  (< 0.05) |  |  |  |  |  |  | 0.9382  (< 0.05) | 0.7482  (< 0.05) |  |  |  |  |  |  |  |  |  |  | 0.9306  (< 0.05) | 0.9196  (< 0.05) |  |  | 0.8451  (< 0.05) |  |  |
| (Ribeiro et al., 2016), Brazil | *Scleronema micranthum (Ducke) Ducke* | DBH (cm) |  |  |  | 0.9195  (< 0.05) |  |  |  |  |  |  | 0.9741  (< 0.05) | 0.8487  (< 0.05) |  |  |  |  |  |  |  |  |  |  | 0.9771  (< 0.05) | 0.9770  (< 0.05) |  |  | 0.9390  (< 0.05) |  |  |
| (Sánchez-Medina et al., 2017), Argentina | *Tabebuia impetiginosa, Tipuana tipu, Citrus × aurantium* | DBH (cm) |  |  |  |  |  |  |  |  |  |  |  | 0.7956  (< 0.05) |  |  |  |  |  |  |  |  |  |  |  |  |  |  |  |  |  |
| (Veselkin, et al. 2015), Russia | *Pinus sylvestris L.* | DBH (cm) | 0.42  (0.011) |  |  |  |  |  |  |  |  |  |  | 0.119  (0.26) |  |  |  |  |  |  |  |  |  |  |  |  |  |  |  |  |  |
| (Kane et al., 2014), USA | *Acer saccharum Marsh.* | DBH/H² |  |  |  |  |  |  |  | 0.32  (> 0.45) |  |  |  |  |  |  |  |  | 0.44  (> 0.15) |  |  |  |  |  |  |  |  |  |  |  |  |
| (Jonsson et al., 2007), Switzerland | *Picea abies (L.) Karst* | DBH/H² |  |  |  |  |  |  |  |  |  |  |  |  |  |  |  |  | 0.56  (< 0.001) |  |  |  |  |  |  |  |  |  |  |  |  |
| (Kane & James, 2011), USA | *Pyrus calleryana Decne 'Bradford* | DBH/H² |  |  |  |  |  |  |  |  |  |  |  |  |  |  |  |  | 0.73  (< 0.01) |  |  |  |  |  |  |  |  |  |  |  |  |
| (Kane & James, 2011), USA | *Quercus prinus L.* | DBH/H² |  |  |  |  |  |  |  |  |  |  |  |  |  |  |  |  | 0.75  (< 0.01) |  |  |  |  |  |  |  |  |  |  |  |  |
| (Cannon et al., 2015), USA | *Liriodendron tulipifera L.* | DBH² |  |  |  |  |  |  |  |  |  |  |  |  |  |  |  |  |  |  |  |  |  |  |  |  |  |  | 0,9220  (< 0.05) |  |  |
| (Peterson & Claassen, 2013), USA | *Populus fremontii Wats.* | DBH² |  |  |  |  |  |  |  |  |  |  |  |  |  |  |  |  |  |  |  |  |  |  |  |  |  |  | 0,9413 (<0.0001) |  |  |
| (Peterson & Claassen, 2013), USA | *Quercus lobata Nee* | DBH² |  |  |  |  |  |  |  |  |  |  |  |  |  |  |  |  |  |  |  |  |  |  |  |  |  |  | 0,9295  (<0.0001) |  |  |
| (Achim et al., 2005), Canada | *Abies balsamea L. Mill.* | DBH²H |  |  |  |  |  |  |  |  |  |  |  |  |  |  |  |  |  |  |  |  |  |  |  |  | 0.9798  (< 0.01) |  |  |  |  |
| (Kamimura et al., 2012), Japan | *Chamaecyparis obtuse*  *(Sieb. Et Zucc.) Endl.* | DBH²H |  |  |  |  |  |  |  |  |  |  |  |  |  |  |  |  |  |  |  |  |  |  |  |  |  |  | 0.670  (0.049) |  |  |
| (Cannon et al., 2015), USA | *Liriodendron tulipifera L.* | DBH²H |  |  |  |  |  |  |  |  |  |  |  |  |  |  |  |  |  |  |  |  |  |  |  |  |  |  | 0,9434  (< 0.05) |  |  |
| (Lundström et al., 2007), Switzerland | *Picea abies L. Karst.* | DBH²H |  | 0.9487  (< 0.001) |  |  |  |  |  |  |  |  |  |  |  |  |  |  |  |  |  |  |  |  |  |  |  |  | 0,8944  (< 0.001) |  |  |
| (Hedden et al., 1995), USA | *Pinus taeda L.* | DBH²H |  |  |  |  |  |  |  |  |  |  |  |  |  |  |  |  |  |  |  |  |  | 0.70  (< 0.0001) |  |  |  |  |  |  |  |
| (Peterson & Claassen, 2013), USA | *Populus fremontii Wats.* | DBH²H |  |  |  |  |  |  |  |  |  |  |  |  |  |  |  |  |  |  |  |  |  |  |  |  |  |  | 0,9731 (<0.0001) |  |  |
| (Peterson & Claassen, 2013), USA | *Quercus lobata Nee* | DBH²H |  |  |  |  |  |  |  |  |  |  |  |  |  |  |  |  |  |  |  |  |  |  |  |  |  |  | 0,9295  (<0.0001) |  |  |
| (Cannon et al., 2015), USA | *Liriodendron tulipifera L.* | DBH³ |  |  |  |  |  |  |  |  |  |  |  |  |  |  |  |  |  |  |  |  |  |  |  |  |  |  | 0,9165  (< 0.05) |  |  |
| (Peltola et al., 2000), Finnland | *Betula spp* | DBH³ |  |  |  |  |  |  |  |  |  |  |  |  |  |  |  |  |  |  |  |  |  | 0.998  (0.0001) |  |  |  |  |  |  |  |
| (Peltola et al., 2000), Finnland | *Picea abies L. Karst* | DBH³ |  |  |  |  |  |  |  |  |  |  |  |  |  |  |  |  |  |  |  |  |  | 0.977  (0.0001) |  |  |  |  |  |  |  |
| (Gardiner et al., 1997), UK | *Picea sitchensis (Bong.) Carr., Larix decidua Mill.* | DBH³ |  |  |  |  |  |  |  |  |  |  |  |  |  |  |  |  |  |  |  |  |  |  |  |  |  |  | 0.5746  (< 0.05) |  |  |
| (Papesch et al., 1997), UK | *Pinus radiata D. Don* | DBH³ |  |  |  |  |  |  |  |  |  |  |  |  |  |  |  |  |  |  |  |  |  |  |  |  |  |  | 0.8544  (< 0.01) |  |  |
| (Peltola et al., 2000), Finnland | *Pinus sylvestris L..* | DBH³ |  |  |  |  |  |  |  |  |  |  |  |  |  |  |  |  |  |  |  |  |  | 0.962  (0.0001) |  |  |  |  |  |  |  |
| (Fredericksen et al., 1993), USA | *Pinus taeda L.* | DBH³ |  |  |  |  |  |  |  |  |  |  |  |  |  |  |  |  |  |  |  |  |  |  |  |  |  |  | 0.9644  (0.0001) |  |  |
| (Cucchi et al., 2004), France | *Pinus pinaster Ait.* | DBH³ (cm³) |  |  |  |  |  |  |  |  |  |  |  |  |  |  |  |  |  |  |  |  |  |  |  |  |  |  | 0.97  (<0.001) |  |  |
| (Peterson & Claassen, 2013), USA | *Populus fremontii Wats.* | DBH³ |  |  |  |  |  |  |  |  |  |  |  |  |  |  |  |  |  |  |  |  |  |  |  |  |  |  | 0,9487 (<0.0001) |  |  |
| (Peterson & Claassen, 2013), USA | *Quercus lobata Nee* | DBH³ |  |  |  |  |  |  |  |  |  |  |  |  |  |  |  |  |  |  |  |  |  |  |  |  |  |  | 0,9022  (<0.0001) |  |  |
| (Hedden et al. 1995), USA | *Pinus taeda L.* | Deflection midstem |  |  |  |  |  |  |  |  |  |  |  |  |  |  |  |  |  |  |  |  |  | -0.4  (< 0.0001) |  |  |  |  |  |  |  |
| (Francis, 2000), USA | 24 different species^2^ | Defoliation |  |  |  |  |  | 0.573  (0.003) |  |  |  |  |  |  | -0.660  (0.000) |  |  |  |  | -0.312  (0.138) |  |  |  | 0.168  (0.432) |  |  |  |  |  |  | -0.256  (0.227) |
| (Veselkin et al., 2015), Russia | *Pinus sylvestris L.* | Degree of defoliation | -0.25  (0.141) |  |  |  |  |  |  |  |  | -0.24 (0.148) |  | -0.10 (0.553) |  |  |  |  |  |  |  |  |  |  |  |  |  |  |  |  |  |
| (Cucchi et al., 2004), France | *Pinus pinaster Ait.* | Depth of root soil plate |  |  |  |  |  |  |  |  |  |  |  |  |  |  |  |  |  |  |  |  |  |  |  | Abies |  |  | 0.77  (<0.001) |  |  |
| (Putz et al., 1983), Panama | Several species^3^ | Diameter growth rate |  |  |  |  |  |  |  |  |  |  |  |  |  |  |  |  |  |  |  |  |  |  |  |  |  |  |  |  | - 0,44  (< 0,01) |
| (Gilmore, 2000), USA | *Larix laricina (Du Roi) K. Koch, Larix decidua Mill., Larix decidua x Larix leptolepis (Sieb. and Zucc.)* | Diameter at base of live crown (cm, measured immediately below lowest) |  |  |  |  |  |  |  |  |  | 0,9607  (< 0.05) |  |  |  |  |  |  |  |  |  |  |  |  |  |  |  |  |  |  |  |
| (Dorren & Berger, 2006), France | *Abies alba* | Energy dissipation rock fall |  |  |  |  |  |  |  |  |  | 0,9592  (< 0.001) |  |  |  |  |  |  |  |  |  |  |  |  |  |  |  |  |  |  |  |
| (Dorren & Berger, 2006), France | *Fagus sylvatica* | Energy dissipation rock fall |  |  |  |  |  |  |  |  |  | 0,9434  (< 0.001) |  |  |  |  |  |  |  |  |  |  |  |  |  |  |  |  |  |  |  |
| (Dorren & Berger, 2006), France | *Picea abies* | Energy dissipation rock fall |  |  |  |  |  |  |  |  |  | 0,8888  (<0.001) |  |  |  |  |  |  |  |  |  |  |  |  |  |  |  |  |  |  |  |
| (Bergeron et al., 2008), Canada | *Picea mariana (Mill.) BSP* | Frontal crown area |  |  |  |  |  |  |  |  |  | 0.70  (<0.0001) |  |  |  |  |  |  |  |  |  |  |  |  |  |  |  |  |  |  |  |
| (Bergeron et al., 2008), Canada | *Pinus banksiana Lamb.* | Frontal crown area |  |  |  |  |  |  |  |  |  | 0.62  (<0.0001) |  |  |  |  |  |  |  |  |  |  |  |  |  |  |  |  |  |  |  |
| (Smith et al., 1987), Canada | *Picea sitchensis (Bong.) Carr.* | Green crown weight |  |  |  |  |  |  |  |  |  |  |  |  |  |  |  |  |  |  |  |  |  |  |  |  |  |  | 0.9414  (1.67) |  |  |
| (Hedden et al., 1995), USA | *Pinus taeda L.* | Green tree weight |  |  |  |  |  |  |  |  |  |  |  |  |  |  |  |  |  |  |  |  |  | 0.71  (< 0.0001) |  |  |  |  |  |  |  |
| (Veselkin et al., 2015), Russia | *Pinus sylvestris L.* | Growth class | 0.04  (0.83) |  |  |  |  |  |  |  |  | -0.02 (0.927) |  | -0.80  (< 0.001) |  |  |  |  |  |  |  |  |  |  |  |  |  |  |  |  |  |
| (Cucchi et al., 2004), France | *Pinus pinaster Ait.* | H (cm) |  |  |  |  |  |  |  |  |  |  |  |  |  |  |  |  |  |  |  |  |  |  |  |  |  |  | 0.83  (<0.001) |  |  |
| (Peltola et al., 2000), Finnland | *Betula spp* | H/DBH |  |  |  |  |  |  |  |  |  |  |  |  |  |  |  |  |  |  | 0.368  (> 0.05) |  |  | 0.904  (< 0.01) |  |  |  |  |  |  |  |
| (Peltola et al., 2000), Finnland | *Picea abies L. Karst* | H/DBH |  |  |  |  |  |  |  |  |  |  |  |  |  |  |  |  |  |  | 0.362  (> 0.05) |  |  | 0.043  (> 0.05) |  |  |  |  |  |  |  |
| (Peltola et al., 2000), Finnland | *Pinus sylvestris L..* | H/DBH |  |  |  |  |  |  |  |  |  |  |  |  |  |  |  |  |  |  | 0.818  (< 0.01) |  |  | 0.861  (< 0.05) |  |  |  |  |  |  |  |
| (Chiba, 2000), Japan | *Cryptomeria japonica D. Don* | Height |  |  |  |  |  |  |  |  |  | 0,9061  (< 0,05) |  |  |  |  |  |  |  |  |  |  |  |  |  |  |  |  |  |  |  |
| (Hedden et al., 1995), USA | *Pinus taeda L.* | Height |  |  |  |  |  |  |  |  |  |  |  |  |  |  |  |  |  |  |  |  |  | 0.67  (< 0.0001) |  |  |  |  |  |  |  |
| (Papesch et al., 1997), UK | *Pinus radiata D. Don* | height |  |  |  |  |  |  |  |  |  |  |  |  |  |  |  |  |  |  |  |  |  |  |  |  |  |  | 0.7836  (< 0.01) |  |  |
| (Peltola et al., 2000), Finnland | *Betula spp* | Height |  |  |  |  |  |  |  |  |  |  |  |  |  |  |  |  |  |  | 0.665  (> 0.05) |  |  | 0.809  (< 0.05) |  |  |  |  |  |  |  |
| (Peltola et al., 2000), Finnland | *Picea abies L. Karst* | Height |  |  |  |  |  |  |  |  |  |  |  |  |  |  |  |  |  |  | 0.634  (< 0.01) |  |  | 0.804  (< 0.05) |  |  |  |  |  |  |  |
| (Peltola et al., 2000), Finnland | *Pinus sylvestris L..* | Height |  |  |  |  |  |  |  |  |  |  |  |  |  |  |  |  |  |  | 0.752  (< 0.01) |  |  | 0.866  (< 0.05) |  |  |  |  |  |  |  |
| (Ribeiro et al., 2016), Brazil | *Eschweilera spp* | Height |  |  |  | 0.7287  (< 0.05) |  |  |  |  |  |  | 0.7670  (< 0.05) |  |  |  |  |  |  |  |  |  |  |  | 0.8388  (< 0.05) | 0.8193  (< 0.05) |  |  | 0.8378  (< 0.05) |  |  |
| (Ribeiro et al., 2016), Brazil | *Scleronema micranthum (Ducke) Ducke* | Height |  |  |  | 0.7893  (< 0.05) |  |  |  |  |  |  | 0.8010  (< 0.05) |  |  |  |  |  |  |  |  |  |  |  | 0.8914  (< 0.05) | 0.8847  (< 0.05) |  |  | 0.7499  (< 0.05) |  |  |
| (Veselkin et al., 2015), Russia | *Pinus sylvestris L.* | Height | 0.33  (0.048) |  |  |  |  |  |  |  |  | 0.26  (0.119) |  |  |  |  |  |  |  |  |  |  |  |  |  |  |  |  |  |  |  |
| (Peterson & Claassen, 2013), USA | *Populus fremontii Wats.* | Height |  |  |  |  |  |  |  |  |  |  |  |  |  |  |  |  |  |  |  |  |  |  |  |  |  |  | 0,8112 (<0.0001) |  |  |
| (Peterson & Claassen, 2013), USA | *Quercus lobata Nee* | Height |  |  |  |  |  |  |  |  |  |  |  |  |  |  |  |  |  |  |  |  |  |  |  |  |  |  | 0,7021 (<0.0001) |  |  |
| (Bergeron et al., 2008), Canada | *Picea mariana (Mill.) B.S.P.* | Height irregular spacing |  |  |  |  |  |  |  |  |  | 0.8113  (0.048) |  |  |  |  |  |  |  |  |  |  |  |  |  |  |  |  |  |  |  |
| (Bergeron et al., 2008), Canada | *Picea mariana (Mill.) B.S.P.* | Height regular spacing |  |  |  |  |  |  |  |  |  | 0.7891  (<0.0001) |  |  |  |  |  |  |  |  |  |  |  |  |  |  |  |  |  |  |  |
| (Cannon et al., 2015), USA | *Liriodendron tulipifera L.*  *Pinus taeda L.* | HxDBH² |  |  |  |  |  |  |  |  |  |  |  |  |  |  |  |  |  |  |  |  |  |  |  |  |  |  | 0,9695  (< 0.05) |  |  |
| (Peltola et al., 2000), Finnland | *Betula spp* | HxDBH² |  |  |  |  |  |  |  |  |  |  |  |  |  |  |  |  |  |  | 0.958  (0.0001) |  |  |  |  |  |  |  |  |  |  |
| (Peltola et al., 2000), Finnland | *Picea abies L. Karst* | HxDBH² |  |  |  |  |  |  |  |  |  |  |  |  |  |  |  |  |  |  | 0.973  (0.0001) |  |  |  |  |  |  |  |  |  |  |
| (Peltola et al., 2000), Finnland | *Pinus sylvestris L..* | HxDBH² |  |  |  |  |  |  |  |  |  |  |  |  |  |  |  |  |  |  | 0.983  (0.0001) |  |  |  |  |  |  |  |  |  |  |
| (Bergeron et al., 2008), Canada | *Picea mariana (Mill.) BSP* | HxDBH² (cm³) |  |  |  |  |  |  |  |  |  |  |  |  |  |  |  |  |  |  |  |  |  |  |  |  | 0.95  (<0.0001) |  |  |  |  |
| (Bergeron et al., 2008), Canada | *Pinus banksiana Lamb.* | HxDBH² (cm³) |  |  |  |  |  |  |  |  |  |  |  |  |  |  |  |  |  |  |  |  |  |  |  |  | 0.96  (<0.0001) |  |  |  |  |
| (Cucchi et al., 2004), France | *Pinus pinaster Ait.* | HxDBH² (cm³) |  |  |  |  |  |  |  |  |  |  |  |  |  |  |  |  |  |  |  |  |  |  |  |  |  |  | 0,9695 (<0.001) |  |  |
| (Fraser, 1962), USA | *Picea sitchensis (Bong.) Carr.* | H x Green Crown Weight |  |  |  |  |  |  |  |  |  |  |  |  |  |  |  |  |  |  |  |  |  |  |  |  |  |  | 0.9414  (< 0.05) |  |  |
| (Francis, 2000), USA | 24 different species^2^ | Leaf retention strength |  |  |  |  |  |  |  |  |  |  |  |  |  |  |  |  |  | 0.500  (0.013) |  |  |  |  |  |  |  |  |  |  | 0.544  (0.006) |
| (Veselkin et al., 2015), Russia | *Pinus sylvestris L.* | Living timber stock | 0.01  (0.956) |  |  |  |  |  |  |  |  | 0.14  (0.415) |  | 0.52  (0.001) |  |  |  |  |  |  |  |  |  |  |  |  |  |  |  |  |  |
| (Ribeiro et al., 2016), Brazil | *Eschweilera spp.* | Maximum critical turning moment |  |  |  |  |  |  |  |  |  |  | 0.8069  (< 0.05) |  |  |  |  |  |  |  |  |  |  |  | 0.8268  (< 0.05) |  |  |  |  |  |  |
| (Ribeiro et al., 2016), Brazil | *Scleronema micranthum (Ducke) Ducke* | Maximum critical turning moment |  |  |  |  |  |  |  |  |  |  | 0.8927  (< 0.05) |  |  |  |  |  |  |  |  |  |  |  | 0.8946  (< 0.05) |  |  |  |  |  |  |
| (Moore, 2000), New Zealand | *Pinus radiata* | Maximum resistive bending moment |  |  |  |  |  |  |  |  |  | 0.91  (0.001) |  | 0.84  (< 0.001) |  |  |  |  |  | -0.312  (< 0.05) |  | 0.3834  (< 0.001) | 0.9241  (<0.001) |  |  |  |  |  |  | 0.84  (<0.001) |  |
| (Stokes, 1999), France | *Pinus pinaster Ait.* | Mean bending moment |  |  |  |  |  |  |  |  |  | 0,9110  (0.002) |  |  |  |  |  |  |  |  |  |  |  |  |  |  |  |  |  |  |  |
| (Onoda et al., 2010), Australia | 32 species^4^ | MOE |  |  |  |  |  |  |  |  |  |  |  |  |  |  |  | 0.925  (<0.000)  0.838  (<0.001)  0.956  (<0.000)  0.978  (<0.000) |  |  |  |  |  |  |  |  |  |  |  |  |  |
| (Asner & Goldstein, 1997), USA | 5 species¹ | MOE |  |  |  |  |  |  |  |  |  | -0.17  (> 0.80) |  |  |  |  |  |  |  |  | -0.70  (0.01) |  |  | 0.84  (0.05) |  |  |  |  |  |  | -0,64  (> 0,20) |
| (Heräjärvi, 2004), Finnland | *Betula pubescens* | MOE |  |  |  |  |  |  |  |  |  |  |  |  |  |  |  |  |  |  |  |  |  |  |  |  |  |  |  |  | 0.663  (< 0,05) |
| (Heräjärvi, 2004), Finnland | *Betulus pendula* | MOE |  |  |  |  |  |  |  |  |  |  |  |  |  |  |  |  |  |  |  |  |  |  |  |  |  |  |  |  | 0.694  (< 0,05) |
| (Heräjärvi, 2004), Finnland | *Betula pubescens* | MOR |  |  |  |  |  |  |  |  |  |  |  |  |  |  | 0.866  (< 0.05) |  |  |  |  |  |  |  |  |  |  |  |  |  | 0.792  (< 0,05) |
| (Heräjärvi, 2004), Finnland | *Betulus pendula* | MOR |  |  |  |  |  |  |  |  |  |  |  |  |  |  | 0.877  (< 0.05) |  |  |  |  |  |  |  |  |  |  |  |  |  | 0.827  (< 0,05) |
| (Putz et al., 1983), Panama | Several species^3^ | Mortality rate |  |  |  |  |  |  |  |  |  |  |  |  |  |  |  |  |  |  |  |  |  |  |  |  |  |  |  |  | - 0,72  (< 0,05) |
| (Veselkin et al., 2015), Russia | *Pinus sylvestris L.* | Needle lifespan | 0.3 (0.068) |  |  |  |  |  |  |  |  | 0.17 (0.305) |  | 0.02 (0.901) |  |  |  |  |  |  |  |  |  |  |  |  |  |  |  |  |  |
| (Francis, 2000), USA | 24 different species^2^ | Resistance to flexing |  |  |  |  |  |  |  |  |  |  |  |  |  |  |  |  |  |  |  |  |  |  |  |  |  |  |  |  | 0.443  (0.030) |
| (Foster, 1988), USA | *Acer rubrum - Quercus borealis* | root failure, stem failure | 0.6633  (< 0.001) |  |  |  |  |  |  |  |  |  |  | 0.6557  (< 0.001) |  |  |  |  |  |  |  |  |  |  |  |  |  |  |  |  |  |
| (Foster, 1988), USA | Conifer stands | root failure, stem failure | 0.7810  (< 0.001) |  |  |  |  |  |  |  |  |  |  | 0.7616  (< 0.001) |  |  |  |  |  |  |  |  |  |  |  |  |  |  |  |  |  |
| (Foster, 1988), USA | Hardwood stands | root failure, stem failure | 0.7746  (< 0.001) |  |  |  |  |  |  |  |  |  |  | 0.6782  (< 0.01) |  |  |  |  |  |  |  |  |  |  |  |  |  |  |  |  |  |
| (Foster, 1988), USA | *Northern* Hardwoods *– Tsuga sp.* | root failure, stem failure | 0.7483  (< 0.05) |  |  |  |  |  |  |  |  |  |  | 0.7348  (< 0.05) |  |  |  |  |  |  |  |  |  |  |  |  |  |  |  |  |  |
| (Foster, 1988), USA | *Pinus resinosa sp.* | root failure, stem failure | 0.6782  (< 0.001) |  |  |  |  |  |  |  |  |  |  | 0.6557  (< 0.01) |  |  |  |  |  |  |  |  |  |  |  |  |  |  |  |  |  |
| (Foster, 1988), USA | *Pinus strobus L.* | root failure, stem failure | 0.8124  (< 0.001) |  |  |  |  |  |  |  |  |  |  | 0.7937  (< 0.001) |  |  |  |  |  |  |  |  |  |  |  |  |  |  |  |  |  |
| (Foster, 1988), USA | *Quercus-Carya-Pinus strobus* | root failure, stem failure | 0.8426  (< 0.01) |  |  |  |  |  |  |  |  |  |  | 0.6557  (< 0.05) |  |  |  |  |  |  |  |  |  |  |  |  |  |  |  |  |  |
| (Kamimura et al., 2012), Japan | *Chamaecyparis obtuse*  *(Sieb. Et Zucc.) Endl.* | Root plate area |  |  |  |  |  |  |  |  |  |  |  |  |  |  |  |  |  |  |  |  |  |  |  |  |  |  | 0.789  (0.011) |  |  |
| (Kamimura et al., 2012), Japan | *Chamaecyparis obtuse*  *(Sieb. Et Zucc.) Endl.* | Root plate volume |  |  |  |  |  |  |  |  |  |  |  |  |  |  |  |  |  |  |  |  |  |  |  |  |  |  | 0.691  (0.039) |  |  |
| (Peltola et al., 2000), Finnland | *Betula spp* | Root-soil plate depth |  |  |  |  |  |  |  |  |  |  |  |  |  |  |  |  |  |  | 0.718  (< 0.05) |  |  | 0.000 |  |  |  |  |  |  |  |
| (Peltola et al., 2000), Finnland | *Picea abies L. Karst* | Root-soil plate depth |  |  |  |  |  |  |  |  |  |  |  |  |  |  |  |  |  |  | 0.495  (< 0.05) |  |  | 0.000 |  |  |  |  |  |  |  |
| (Peltola et al., 2000), Finnland | *Pinus sylvestris L..* | Root-soil plate depth |  |  |  |  |  |  |  |  |  |  |  |  |  |  |  |  |  |  | 0.693  (< 0.01) |  |  | 0.000 |  |  |  |  |  |  |  |
| (Peltola et al., 2000), Finnland | *Betula spp* | Root-soil plate radius |  |  |  |  |  |  |  |  |  |  |  |  |  |  |  |  |  |  | 0.661  (> 0.05) |  |  | 0.000 |  |  |  |  |  |  |  |
| (Peltola et al., 2000), Finnland | *Picea abies L. Karst* | Root-soil plate radius |  |  |  |  |  |  |  |  |  |  |  |  |  |  |  |  |  |  | 0.606  (> 0.05) |  |  | 0.000 |  |  |  |  |  |  |  |
| (Peltola et al., 2000), Finnland | *Pinus sylvestris L..* | Root-soil plate radius |  |  |  |  |  |  |  |  |  |  |  |  |  |  |  |  |  |  | 0.476  (< 0.05) |  |  | 0.000 |  |  |  |  |  |  |  |
| (Veselkin et al., 2015), Russia | *Pinus sylvestris L.* | Share of deadwood in timber stock | 0.33 (0.043) |  |  |  |  |  |  |  |  | -0.04  (0.814) |  | 0.15  (0.386) |  |  |  |  |  |  |  |  |  |  |  |  |  |  |  |  |  |
| (Fredericksen et al., 1993), USA | *Pinus taeda L.* | Soil moisture content |  |  |  |  |  |  |  |  |  |  |  |  |  | 0.01  (0.52) |  |  |  |  |  |  |  |  |  |  |  |  |  |  |  |
| (Silins et al., 2000), Canada | *Pinus contorta var. latifolia Engelm.* | Specific gravity/wood density |  |  |  |  |  |  |  |  |  |  |  |  |  |  | 0.253  (< 0.05) |  |  |  |  |  |  |  |  |  |  |  |  |  |  |
| (Silins et al., 2000), Canada | *Pinus contorta var. latifolia Engelm.* | Spring (temp. ± 8,8° C) |  |  |  |  |  |  |  |  |  |  |  |  |  |  | 0.1  (> 0.05) | 0.28  (> 0.05) |  |  |  |  |  |  |  |  |  |  |  |  |  |
| (Foster, 1988), USA | *Acer rubrum - Quercus borealis* | Standing trees | 0.82  (0.001) |  |  |  |  |  |  |  |  |  |  |  |  |  |  |  |  |  |  |  |  |  |  |  |  |  |  |  |  |
| (Foster, 1988), USA | Hardwood stands | Standing trees | 0.91  (0.001) |  |  |  |  |  |  |  |  |  |  |  |  |  |  |  |  |  |  |  |  |  |  |  |  |  |  |  |  |
| (Foster, 1988), USA | *Pinus resinosa sp.* | Standing trees | 0.68  (0.001) |  |  |  |  |  |  |  |  |  |  |  |  |  |  |  |  |  |  |  |  |  |  |  |  |  |  |  |  |
| (Foster, 1988), USA | *Pinus strobus L.* | Standing trees | 0.82  (0.001) |  |  |  |  |  |  |  |  |  |  |  |  |  |  |  |  |  |  |  |  |  |  |  |  |  |  |  |  |
| (Foster, 1988), USA | *Quercus-Carya-Pinus strobus* | Standing trees | 0.61  (0.001) |  |  |  |  |  |  |  |  |  |  |  |  |  |  |  |  |  |  |  |  |  |  |  |  |  |  |  |  |
| (Fredericksen et al., 1993), USA | *Pinus taeda L.* | Stem deflection at breast height (degrees) |  |  |  |  |  |  |  |  |  |  |  |  |  |  |  |  |  |  |  |  |  |  |  | 0.4899 (0.0002) |  |  |  |  |  |
| (Fredericksen et al., 1993), USA | *Pinus taeda L.* | Stem deflection at midstem (degrees) |  |  |  |  |  |  |  |  |  |  |  |  |  |  |  |  |  |  |  |  |  |  |  | 0.7071  (0.0001) |  |  |  |  |  |
| (Francis, 2000), USA | 24 different species^2^ | Stem failure |  |  |  |  |  |  |  |  |  |  |  |  | -0.227  (0.287) |  |  |  |  | -0.010  (0.961) |  |  |  |  |  |  |  |  |  |  | -0.460  (0.024) |
| (Ribeiro et al., 2016), Brazil | *Eschweilera spp.* | Stem fresh wood density (g.cm^-3^) |  |  |  | 0.1307  (< 0.05) |  |  |  |  |  | -0.0179  (< 0.05) | 0.0029  (< 0.05) | 0.0188  (< 0.05) |  |  |  |  |  |  |  |  |  |  | -0.1114  (< 0.05) | -0.1896  (< 0.05) |  | 0.1412  (< 0.05) | -0.0664  (< 0.05) |  |  |
| (Ribeiro et al., 2016), Brazil | *Scleronema micranthum (Ducke) Ducke* | Stem fresh wood density (g.cm^-3^) |  |  |  | 0.1965  (< 0.05) |  |  |  |  |  | 0.1784  (< 0.05) | 0.1789  (< 0.05) | 0.1614  (< 0.05) |  |  |  |  |  |  |  |  |  |  | 0.1778  (< 0.05) | 0.0969  (< 0.05) |  | 0.1807  (< 0.05) | 0.1987  (< 0.05) |  |  |
| (Cannon et al., 2015), USA | *Liriodendron tulipifera L.* | Stem mass (kg) |  |  |  |  |  |  |  |  |  |  |  |  |  |  |  |  |  |  |  |  |  |  |  |  |  |  | 0,9592  (< 0.05) |  |  |
| (Ribeiro et al., 2016), Brazil | *Eschweilera spp.* | Stem mass (kg) |  |  |  |  |  |  |  |  |  |  | 0.9366  (< 0.05) |  |  |  |  |  |  |  |  |  |  |  |  |  |  |  | 0.8268  (< 0.05) |  |  |
| (Ribeiro et al., 2016), Brazil | *Scleronema micranthum* | Stem mass (kg) |  |  |  |  |  |  |  |  |  |  | 0.9444  (< 0.05) |  |  |  |  |  |  |  |  |  |  |  |  |  |  |  | 0,8946  (< 0.05) |  |  |
| (Fredericksen et al., 1993), USA | *Pinus taeda L.* | Stem moisture content |  |  |  |  |  |  |  |  |  |  |  |  |  | 0.04  (0.27) |  |  |  |  |  |  |  |  |  |  |  |  |  |  |  |
| (Cucchi et al., 2004), France | *Pinus pinaster Ait.* | Stem taper (H/DBH) |  |  |  |  |  |  |  |  |  |  |  |  |  |  |  |  |  |  |  |  |  |  |  |  |  |  | -0.69  (<0.001) |  |  |
| (Lundström et al., 2007), Switzerland | *Picea abies L. Karst.* | Stem volume |  | 0.9644  (< 0.001) |  |  |  |  |  |  |  |  |  |  |  |  |  |  |  |  |  |  |  |  |  |  |  |  | 0,9592  (< 0.001) |  |  |
| (Fredericksen et al., 1993), USA | *Pinus taeda L.* | Stem volume |  |  |  |  |  |  |  |  |  |  |  |  |  |  |  |  |  |  |  |  |  |  |  |  |  |  | 0.9695  (0.0001) |  |  |
| (Hedden et al., 1995), USA | *Pinus taeda L.* | Stem volume |  |  |  |  |  |  |  |  |  |  |  |  |  |  |  |  |  |  |  |  |  | 0.7  (0.0001) |  |  |  |  |  |  |  |
| (Ribeiro et al., 2016), Brazil | *Eschweilera spp.* | Stem volume (kg) |  |  |  | 0.7743  (< 0.05) |  |  |  |  |  |  | 0.9262  (< 0.05) |  |  |  |  |  |  |  |  |  |  |  | 0.9948  (< 0.05) |  |  |  | 0.8248  (< 0.05) |  |  |
| (Ribeiro et al., 2016), Brazil | *Scleronema micranthum (Ducke) Ducke* | Stem volume (kg) |  |  |  | 0.9169  (< 0.05) |  |  |  |  |  |  | 0.9405  (< 0.05) |  |  |  |  |  |  |  |  |  |  |  | 0.9953  (< 0.05) |  |  |  | 0.9003  (< 0.05) |  |  |
| (Achim et al., 2003), UK | *Picea sitchensis (Bong.) Carr.* | Stem weight (kg) |  |  |  |  |  |  |  |  |  |  |  |  |  |  |  |  |  |  |  |  |  |  |  |  |  |  | 0.8544  (<0.001) |  |  |
| (Achim et al., 2005), Canada | *Abies balsamea (L.) Mill.* | Stem weight |  |  |  |  |  |  |  |  |  |  |  |  |  |  |  |  |  |  |  |  |  |  |  |  |  |  | 0,9  (< 0.05) |  |  |
| (Achim et al., 2005), Canada | *Abies balsamea L. Mill.* | Stem weight (kg) |  |  | 0.67  (< 0.01) |  | 0.57  (< 0.01) |  |  |  |  |  |  |  |  |  |  |  |  |  |  |  |  |  |  |  |  |  | 0.89  (<0.01) |  |  |
| (Bergeron et al., 2008), Canada | *Picea mariana (Mill.) BSP* | Stem weight (kg) |  |  |  |  |  |  |  |  |  |  |  |  |  |  |  |  |  |  |  |  |  |  |  |  |  |  | 0.9165  (0.0001) |  |  |
| (Bergeron et al., 2008), Canada | *Pinus banksiana Lamb.* | Stem weight (kg) |  |  |  |  |  |  |  |  |  |  |  |  |  |  |  |  |  |  |  |  |  |  |  |  |  |  | 0.9110  (0.0001) |  |  |
| (Bergeron et al., 2008), Canada | *Pinus contorta var. latifolia Engelm.* | Stem weight (kg) |  |  |  |  |  |  |  |  |  |  |  |  |  |  |  |  |  |  |  |  |  |  |  |  |  |  | 0.8660  (0.0002) |  |  |
| (Bergeron et al., 2008), Canada | *Picea mariana (Mill.) BSP* | Stem weight (kg), stony soil |  |  |  |  |  |  |  |  |  |  |  |  |  |  |  |  |  |  |  |  |  |  |  |  |  |  | 0.8944  (0.0001) |  |  |
| (Bergeron et al., 2008), Canada | *Pinus banksiana Lamb.* | Stem weight (kg), stony soil |  |  |  |  |  |  |  |  |  |  |  |  |  |  |  |  |  |  |  |  |  |  |  |  |  |  | 0.9381  (0.0001) |  |  |
| (Cannon et al., 2015), USA | *Liriodendron tulipifera L.*  *Pinus taeda L.* | Stem weight |  |  |  |  |  |  |  |  |  |  |  |  |  |  |  |  |  |  |  |  |  |  |  |  |  |  | 0,9798  (< 0.05) |  |  |
| (Cucchi et al., 2004), France | *Pinus pinaster Ait.* | Stem weight (kg) |  |  |  |  |  |  |  |  |  |  |  |  |  |  |  |  |  |  |  |  |  |  |  |  |  |  | 0.9695  (<0.001) |  |  |
| (Fraser, 1962), UK | *Picea sitchensis (Bong.) Carr.*  *Pseudotsuga taxifolia (Lamb.)* | Stem weight |  |  |  |  |  |  |  |  |  |  |  |  |  |  |  |  |  |  |  |  |  |  |  |  |  |  | 0,8150  (< 0.05) |  |  |
| (Gardiner et al.,1997), UK | *Picea sitchensis (Bong.) Carr., Larix decidua Mill.* | Stem weight |  |  |  |  |  |  |  |  |  |  |  |  |  |  |  |  |  |  |  |  |  |  |  |  |  |  | 0.7211  (< 0.05) |  |  |
| (Lundström et al., 2007), Switzerland | *Picea abies L. Karst.* | Stem weight (kg) |  | 0.9644  (< 0.001) |  |  |  |  |  |  |  |  |  |  |  |  |  |  |  |  |  |  |  |  |  |  |  |  |  |  |  |
| (Peltola et al., 2000), FInnland | *Betula spp* | Stem weight |  |  |  |  |  |  |  |  |  |  |  |  |  |  |  |  |  |  | 0.79  (< 0.05) |  |  | 0.000 |  |  |  |  |  |  |  |
| (Peltola et al., 2000), Finnland | *Picea abies L. Karst* | Stem weight |  |  |  |  |  |  |  |  |  |  |  |  |  |  |  |  |  |  | 0.974  (< 0.01) |  |  | 0.964  (< 0.01) |  |  |  |  |  |  |  |
| (Peltola et al., 2000), Finnland | *Pinus sylvestris L..* | Stem weight |  |  |  |  |  |  |  |  |  |  |  |  |  |  |  |  |  |  | 0.972  (< 0.01) |  |  | 0.000 |  |  |  |  |  |  |  |
| (Ribeiro et al., 2016), Brazil | *Eschweilera spp.* | Stem weight |  |  |  |  |  |  |  |  |  |  |  |  |  |  |  |  |  |  |  |  |  |  |  |  |  |  | 0,8268  (< 0.05) |  |  |
| (Ribeiro et al., 2016), Brazil | *Scleronema micranthum (Ducke) Ducke* | Stem weight |  |  |  |  |  |  |  |  |  |  |  |  |  |  |  |  |  |  |  |  |  |  |  |  |  |  | 0.8946  (< 0.05) |  |  |
| (Fredericksen et al., 1993), USA | *Pinus taeda L.* | Taper (height / diameter) |  |  |  |  |  |  |  |  |  |  |  |  |  |  |  |  |  |  |  |  |  |  |  |  |  |  | 0.9539  (0.0001) |  |  |
| (Hedden et al., 1995), USA | *Pinus taeda L.* | Taper (height / diameter) |  |  |  |  |  |  |  |  |  |  |  |  |  |  |  |  |  |  |  |  |  | -0.61  (< 0.0001) |  |  |  |  |  |  |  |
| (Peterson & Claassen, 2013), USA | *Populus fremontii Wats.* | Taper |  |  |  |  |  |  |  |  |  |  |  |  |  |  |  |  |  |  |  |  |  |  |  |  |  |  | 0,7523 (<0.0001) |  |  |
| (Peterson & Claassen, 2013), USA | *Quercus lobata Nee* | Taper |  |  |  |  |  |  |  |  |  |  |  |  |  |  |  |  |  |  |  |  |  |  |  |  |  |  | 0,6542 (<0.0001) |  |  |
| (Hedden et al., 1995), USA | *Pinus taeda L.* | Total green tree weight |  |  |  |  |  |  |  |  |  |  |  |  |  |  |  |  |  |  |  |  |  | 0.71  (< 0.0001) |  |  |  |  |  |  |  |
| (Ribeiro et al., 2016), Brazil | *Eschweilera spp.* | Tree center of mass |  |  |  | 0.8117  (< 0.05) |  |  |  |  |  | 0.6504  (< 0.05) | 0.6492  (< 0.05) | 0.8517  (< 0.05) |  |  |  |  |  |  |  |  |  |  | 0.6983  (< 0.05) | 0.6766  (< 0.05) |  |  | 0.7820  (< 0.05) |  |  |
| (Ribeiro et al., 2016), Brazil | *Scleronema micranthum (Ducke) Ducke* | Tree center of mass |  |  |  | 0.8337  (< 0.05) |  |  |  |  |  | 0.7675  (< 0.05) | 0.7343  (< 0.05) | 0.8108  (< 0.05) |  |  |  |  |  |  |  |  |  |  | 0.8159  (< 0.05) | 0.8100  (< 0.05) |  |  | 0.6633  (< 0.05) |  |  |
| (Fredericksen et al., 1993), USA | *Pinus taeda L.* | Tree height + tree height² |  |  |  |  |  |  |  |  |  |  |  |  |  |  |  |  |  |  |  |  |  |  |  |  |  |  | 0.7937  (0.0001) |  |  |
| (Cannon et al., 2015), USA | *Liriodendron tulipifera L.* | Tree mass (kg) |  |  |  |  |  |  |  |  |  |  |  |  |  |  |  |  |  |  |  |  |  |  |  |  |  |  | 0,9487  (< 0.05) |  |  |
| (Kane & Smiley, 2006), USA | *Acer rubrum L.* | Tree mass (kg) |  |  |  |  |  |  |  |  |  | 0.95  (<0.001) |  |  |  |  |  |  |  |  |  |  |  |  |  |  |  |  |  |  |  |
| (Peterson & Claassen, 2013), USA | *Populus fremontii Wats.* | Tree mass |  |  |  |  |  |  |  |  |  |  |  |  |  |  |  |  |  |  |  |  |  |  |  |  |  |  | 0,9644 (<0.0001) |  |  |
| (Peterson & Claassen, 2013), USA | *Quercus lobata Nee* | Tree mass |  |  |  |  |  |  |  |  |  |  |  |  |  |  |  |  |  |  |  |  |  |  |  |  |  |  | 0,9628 (<0.0001) |  |  |
| (Veselkin et al., 2015), Russia | *Pinus sylvestris L.* | Tree stand density | -0.31  (0.059) |  |  |  |  |  |  |  |  | -0.87  (< 0.001) |  | -0.19 (0.249) |  |  |  |  |  |  |  |  |  |  |  |  |  |  |  |  |  |
| (Kamimura et al., 2012), Japan | *Chamaecyparis obtuse*  *(Sieb. Et Zucc.) Endl.* | Tree weight |  |  |  |  |  |  |  |  |  |  |  |  |  |  |  |  |  |  |  |  |  |  |  |  |  |  | 0.683  (0.043) |  |  |
| (Fredericksen et al., 1993), USA | *Pinus taeda L.* | Tree weight |  |  |  |  |  |  |  |  |  |  |  |  |  |  |  |  |  |  |  |  |  |  |  | 0.4899  (0.0002) |  |  | 0.9798  (0.0001) |  |  |
| (Lundström et al., 2007), Switzerland | *Picea abies L. Karst.* | Tree weight (kg) |  | 0.9644  (< 0.001) |  |  |  |  |  |  |  |  |  |  |  |  |  |  |  |  |  |  |  |  |  |  |  |  |  |  |  |
| (Fraser, 1962), UK | *Picea sitchensis (Bong.) Carr.* | Tree weight (kg) |  |  |  |  |  |  |  |  |  |  |  |  |  |  |  |  |  |  |  |  |  |  |  |  |  |  | 0.81  (< 0.05) |  |  |
| ([King, 1986](#_ENREF_1)), USA | *Acer saccharum Marsh.* | Trunk safety factor |  |  |  |  |  |  |  |  |  | 0.45  (< 0.001) |  |  |  |  |  |  |  |  |  |  |  |  |  |  |  |  |  |  |  |
| (Papesch et al., 1997), UK | *Pinus radiata D. Don* | Volume |  |  |  |  |  |  |  |  |  |  |  |  |  |  |  |  |  |  |  |  |  |  |  |  |  |  | 0.8307  (< 0.01) |  |  |
| (Cucchi et al., 2004), France | *Pinus pinaster Ait.* | Volume of the soil-root plate (m³) |  |  |  |  |  |  |  |  |  |  |  |  |  |  |  |  |  |  |  |  |  |  |  |  |  |  | 0.91  (<0.001) |  |  |
| (Kamimura et al., 2012), Japan | *Chamaecyparis obtuse*  *(Sieb. Et Zucc.) Endl.* | Water content below the root plate |  |  |  |  |  |  |  |  |  |  |  |  |  |  |  |  |  |  |  |  |  |  |  |  |  |  | -0.728  (0.026) |  |  |
| (Kamimura et al., 2012), Japan | *Chamaecyparis obtuse*  *(Sieb. Et Zucc.) Endl.* | Water content inside the root plate |  |  |  |  |  |  |  |  |  |  |  |  |  |  |  |  |  |  |  |  |  |  |  |  |  |  | 0.827  (0.011) |  |  |
| (Hale et al., 2012), UK | *Picea sitchensis (Bong.) Carr.* | Wind speed canopy |  |  |  |  |  |  |  |  |  |  |  |  |  |  |  |  |  |  |  |  |  |  |  |  |  |  | 0.9465  (<0.000) |  |  |
| (Hale et al., 2012), UK | *Larix decidua Mill* | Wind speed canopy |  |  |  |  |  |  |  |  |  |  |  |  |  |  |  |  |  |  |  |  |  |  |  |  |  |  | 0.9672  (<0.000) |  |  |
| ([King, 1986](#_ENREF_1)), USA | *Acer saccharum Marsh.* | Wind speed canopy |  |  |  |  |  |  |  |  |  | -0.15  (< 0.20) |  |  |  |  |  |  |  |  |  |  |  |  |  |  |  |  |  |  |  |
| ([King, 1986](#_ENREF_1)), USA | *Acer saccharum Marsh.* | Wind speed maximum |  |  |  |  |  |  |  |  |  | 0.41  (< 0.001) |  |  |  |  |  |  |  |  |  |  |  |  |  |  |  |  |  |  |  |
| (Silins et al., 2000), Canada | *Pinus contorta var. latifolia Engelm.* | Winter (temp. < 0° C) |  |  |  |  |  |  |  |  |  |  |  |  |  |  | 0.608  (< 0.01) | 0.35  (> 0.05) |  |  |  |  |  | 37 |  |  |  |  |  |  |  |
| (Asner & Goldstein, 1997), USA | 5 species¹ | Wood density / Specific gravity |  |  |  |  |  |  |  |  |  |  |  |  |  |  |  |  |  |  | 0.25  (> 0.05) |  |  | -0.45  (> 0.05) |  |  |  |  |  |  |  |
| Total number of correlations | | 531 | 24 | 25 | 7 | 14 | 16 | 5 | 2 | 3 | 3 | 37 | 54 | 49 | 34 | 3 | 6 | 9 | 7 | 9 | 34 | 31 | 4 | 39 | 60 | 3 | 4 | 7 | 28 | 2 | 12 |
| Author (year) | Species | Variables | Age | Anchorage strength (Maximum M (Nm)) | Crown depth | Crown mass | Crown length | Crown loss | Crown ratio | Damping ratio °ξ | Degree of spread | DBH | Diameter stump | Height | Leaf retention strength | Maximum declination at midstem | MOE | MOR | Natural frequency ƒn | Resistance to flexing | Root failure | root plate depth (height) | root plate volume | Stem failure | Stem mass | Stem volume | Stem weight | tree center of mass | Turning moment (kN m) | vertical radius root plate (width) | Wood density/Specific gravity |

1. *Metrosideros polymorpha Gaudich., Acacia koa A. Gray, Grevillea robusta A. Cunn., Acacia mearnsii De Wild., Eucalyptus robusta Sm.*
2. Swietenia mahagoni L. Jacq., Hymenaea courbarii var. sp., Calophyllum calaba L., Pinus caribaea sp., Melaleuca quinquinervia (Cav.) S.T. Blake, Delonix regia (Bojer) Raf., Lagerstroemia speciose Pers., Termlnalia catappa L., Tabebuia heterophylla Britton, Cassia javanica Vell., Casuarlna equisetifolia L., Pterocarpus macrocarpus Kurz, Bucida buceras Vell., Enterolobium cyclocarpum (Jacq.) Griseb., Mangifera indica L., Clitoria fairchieldiana R.A. Howard., Ficus benjamina L., Schefflera morototoni (Aubl.) Maguire, Steyerm. & Frodin, Albizia procera (Roxb.) Benth., Cecropia schreberiana Micq., Sterculia apetala (Jacq.) H. Karst., Peltophorum pterocarpum Backer ex K.Heyne, Senna siamea (Lam.) H.S. Irwin & Barneby, Spathodea campanulata P.Beauv.
3. Due to a bias in the field methods, only the following species are determined to be part of this investigation: Anacardium excelsum (Bertero and Balb.) Skeels (Anacardiaceae), Cavanillesia platanifolia (H. and B.) H.B.K., Ceiba pentandra L. Gaertn. (Bombacaceae), Enterolobium cyciocarpum (Jacq.) Griseb. (Leguminosae), Poulsenia armata (Miq.) StandI. (Moraceae), Virola sebifera Aubl., Virola surinamensis (Rol.) Warb. (Myristicaceae), Pouteria stipitata Cronq. (Sapotaceae), Terminalia amazonica (1. F. Gmel.) Exell in Pulle (Combretaceae), Jacaranda copaia (Aubl.) D. Don (Bignoniaceae)
4. Acacia doratoxylon Meisn., Acacia floribunda Wildd., Acacia havilandiorum Maiden, Acacia suaveolens Willd., Astrotricha floccosa DC., Bertya cunninghamii Planch., Beyeria opaca F.Muell., Boronia ledifolia (Vent.) DC., Brachychiton populneus R.Br., Cassinia laevis R.Br., Dodonaea viscosa f. angustissima (L.f.) Sherff, Eremophila glabra Ostenf., Eremophila longifolia F.Muell., Eriostemon australasius Pers., Eucalyptus socialis F.Muell. ex Miq., Geijera parviflora Lindl., Hakea dactyloides Cav., Hakea tephrosperma R.Br., Lambertia formosa Sm., Lasiopetalum ferrugineum Sm., Leptospermum trinervium (Sm.) Joy Thomps., Lomatia silaifolia R.Br., Melaleuca uncinata R.Br., Olearia pimeleoides Benth., Persoonia levis (Cav.) Domin, Persoonia linearis Sieber ex Schult., Philotheca difformis (A. Cunn. ex Endl.) Paul G. Wilson, Phyllota phylicoides Benth., Pultenaea flexilis Sm., Senna artemisioides (Gaudich. ex A.DC.) Kartesz & Gandhi, Syncarpia glomulifera Nied., Synoum glandulosum A.Juss.
5. DBH = diameter breast height, DBH/H² = diameter breast height divided by height squared, DBH²H = diameter breast height squared times height, DBH² = diameter breast height squared, DBH³ = diameter breast height cubed, H = height, H/DBH = height divided by diameter breast height, H x Green Crown Weight = height times green crown weight, MOE = modulus of elasticity, MOR = modulus of rupture.
