# Supplementary material for: Understanding tree failure—A systematic review and meta-analysis
Source: PLoS One. 2021 Feb 16;16(2):e0246805. doi: 10.1371/journal.pone.0246805 (PMC7886209; doi:10.1371/journal.pone.0246805)
Supplement: S2 Table — (DOCX) [file pone.0246805.s003.docx]

S2 Table: Overview of all effect sizes (correlations) mentioned in studies of root failure (Note: factors can be labelled horizontal and vertical)

| Root failure | Variables^1^ | | Anchorage  strength | Root depth | Root failure | Root spread | Root soil plate  depth | Root soil  plate length | Root volume | Soil type | Stem volume | Turning moment | vertical radius  root plate (width) |
| --- | --- | --- | --- | --- | --- | --- | --- | --- | --- | --- | --- | --- | --- |
| Author(year) | Species |  |  |  |  |  |  |  |  |  |  |  |  |
| (Smith, 1964), Canada | *Pinus contorta Bol.* | Age |  |  |  | 0.659  (< 0.05) |  |  |  |  |  |  |  |
| (Smith, 1964), Canada | *Pinus contorta Bol.* | Age |  |  |  | 0.427  (< 0.05) |  |  |  |  |  |  |  |
| (Smith, 1964), Canada | *Pseudotsuga menziesii (Mirb)* | Age |  |  |  | 0.500  (< 0.05) |  |  |  |  |  |  |  |
| (Peltola et al., 2000), Finnland | *Pinus sylvestris L.* | Angle of stem at maximum moment applied |  |  | -0.513  (< 0.01) |  |  |  |  |  |  |  |  |
| (Peltola et al., 2000), Finnland | *Picea abies L. Karst.* | Angle of stem at maximum moment applied |  |  | -0.406  (< 0.05) |  |  |  |  |  |  |  |  |
| (Smith, 1964), Canada | *Alnus rubra Bong.* | Average branch |  |  |  | 0.518  (0.01) |  |  |  |  |  |  |  |
| (Smith, 1964), Canada | *Pseudotsuga menziesii (Mirb)* | Average branch |  |  |  | 0.567  (0.01) |  |  |  |  |  |  |  |
| (Smith, 1964), Canada | *Thuja plicata Donn.* | Average branch |  |  |  | 0.594  (0.01) |  |  |  |  |  |  |  |
| (Smith, 1964), Canada | *Tsuga heterophylla (Raf.) Sarg.* | Average branch |  |  |  | 0.661  (0.01) |  |  |  |  |  |  |  |
| (Smith, 1964), Canada | *Alnus rubra Bong.* | Average root diameter |  |  |  | 0.260  (> 0.05) |  |  |  |  |  |  |  |
| (Smith, 1964), Canada | *Pseudotsuga menziesii (Mirb)* | Average root diameter |  |  |  | 0.282  (< 0.01) |  |  |  |  |  |  |  |
| (Smith, 1964), Canada | *Thuja plicata Donn.* | Average root diameter |  |  |  | 0.182  (> 0.05) |  |  |  |  |  |  |  |
| (Smith, 1964), Canada | *Tsuga heterophylla (Raf.) Sarg.* | Average root diameter |  |  |  | 0.208  (> 0.05) |  |  |  |  |  |  |  |
| (Smith, 1964), Canada | *Pseudotsuga menziesii (Mirb)* | Bark thickness |  |  |  | 0.512  (< 0.05) |  |  |  |  |  |  |  |
| (Gilman, 1989), USA | *Acer rubrum L.* | Branch crown radius |  |  |  | 0.97  (< 0.01) |  |  |  |  |  |  |  |
| (Gilman, 1989), USA | *Fraxinus pennsylvanica Marsh.* | Branch crown radius |  |  |  | 0.99  (< 0.01) |  |  |  |  |  |  |  |
| (Gilman, 1989), USA | *Gleditsia triacanthos var. Inermis L. C.K. Schneid.* | Branch crown radius |  |  |  | 0.99  (< 0.01) |  |  |  |  |  |  |  |
| (Gilman, 1989), USA | *Magnolia grandiflora L.* | Branch crown radius |  |  |  | 0.98  (< 0.01) |  |  |  |  |  |  |  |
| (Gilman, 1989), USA | *Populus × generosa A.Henry* | Branch crown radius |  |  |  | 0.74  (< 0.05) |  |  |  |  |  |  |  |
| (Gilman, 1989), USA | *Quercus virginiana Mill.* | Branch crown radius |  |  |  | 0.23  (> 0.05) |  |  |  |  |  |  |  |
| (Mueller & Cline, 1958), USA | *Prunus serotina Schur.; Ulmus americana L., Acer sacharum Marsh.; Liriodendron tulipifera L.* | Combined calcareous till |  | -0.426  (>0.05) |  | 0.732  (< 0.01) |  |  | 0.656  (< 0.01) |  |  |  |  |
| (Mueller & Cline, 1958), USA | *Prunus serotina Schur.; Ulmus americana L., Acer sacharum Marsh.; Liriodendron tulipifera L.* | Combined fragipan |  | 0.910  (< 0.01) |  | 0.556  (< 0.05) |  |  | 0.639  (< 0.05) |  |  |  |  |
| (Peltola et al., 2000), Finnland | *Pinus sylvestris L.* | Crown area |  |  | 0.875  (< 0.01) |  |  |  |  |  |  |  |  |
| (Peltola et al., 2000), Finnland | *Picea abies L. Karst.* | Crown area |  |  | 0.893  (< 0.01) |  |  |  |  |  |  |  |  |
| (Peltola et al., 2000), Finnland | *Betula spp.* | Crown area |  |  | 0.827  (< 0.05) |  |  |  |  |  |  |  |  |
| (Stokes et al., 2007), France | *Fagus sylvatica L.* | Crown biomass (kg) |  |  |  |  |  |  |  |  |  | 0.8124  (< 0.027) |  |
| (Stokes et al., 2007), France | *Picea alba Mill.* | Crown biomass (kg) |  |  |  |  |  |  |  |  |  | 0.9592  (< 0.002) |  |
| (Smith, 1964), USA | *Alnus rubra Bong.* | Crown length |  |  |  | 0.407  (0.01) |  |  |  |  |  |  |  |
| (Smith, 1964), USA | *Pseudotsuga menziesii (Mirb)* | Crown length |  |  |  | 0.546  (0.01) |  |  |  |  |  |  |  |
| (Smith, 1964), USA | *Thuja plicata Donn.* | Crown length |  |  |  | 0.545  (0.01) |  |  |  |  |  |  |  |
| (Smith, 1964), USA | *Tsuga heterophylla (Raf.) Sarg.* | Crown length |  |  |  | 0.659  (0.01) |  |  |  |  |  |  |  |
| (Danjon, Fourcaud, & Bert, 2005), France | *Pinus pinaster Ait.* | Crown ratio |  |  |  |  |  |  |  |  | 0.62  (<0.05) |  |  |
| (Smith, 1964), USA | *Picea glauca (Moench) Voss* | Crown width |  |  |  | 0.9476  (< 0.05) |  |  |  |  |  |  |  |
| (Smith, 1964), USA | *Pinus contorta Bol.* | Crown width |  |  |  | 0.9359  (< 0.05) |  |  |  |  |  |  |  |
| (Smith, 1964), USA | *Pinus contorta Bol.* | Crown width |  |  |  | 0.711  (< 0.05) |  |  |  |  |  |  |  |
| (Smith, 1964), USA | *Pinus contorta Bol.* | Crown width |  |  |  | 0.707  (< 0.05) |  |  |  |  |  |  |  |
| (Smith, 1964), USA | *Pinus contorta Bol.* | Crown width |  |  |  | 0.505  (< 0.05) |  |  |  |  |  |  |  |
| (Smith, 1964), USA | *Pinus ponderosa Lawsoniana* | Crown width |  |  |  | 0.9028  (< 0.05) |  |  |  |  |  |  |  |
| (Smith, 1964), USA | *Pseudotsuga menziesii (Mirb)* | Crown width |  |  |  | 0.7301  (0.01) |  |  |  |  |  |  |  |
| (Gilman, 1989), USA | *Acer rubrum L.* | DBH |  |  |  | 0.82  (0.05) |  |  |  |  |  |  |  |
| (Smith, 1964), USA | *Alnus rubra Bong.* | DBH |  |  |  | 0.610  (0.01) |  |  |  |  |  |  |  |
| (Gerhold & Johnson, 2003), USA | *Amelanchier 'Cumulus'* | DBH |  | 0.5477  (0.0001) |  | 0.8718  (< 0.05) |  |  |  |  |  |  |  |
| (Gerhold & Johnson, 2003), USA | *Amelanchier 'Snowcloud'* | DBH |  | 0.5477  (0.0001) |  | 0.8718  (< 0.05) |  |  |  |  |  |  |  |
| (Gerhold & Johnson, 2003), USA | *Amelanchier, Malus, Pyrus, Syringa* | DBH |  | 0.5477  (0.0001) |  | 0.8718  (< 0.05) |  |  |  |  |  |  |  |
| (Peltola et al., 2000), Finnland | *Betula spp.* | DBH |  |  | 0.809  (< 0.05) |  |  |  |  |  |  |  |  |
| (Stokes et al., 2007), France | *Fagus sylvatica L.* | DBH |  |  |  |  |  |  |  |  |  | 0.8718  (< 0.011) |  |
| (Gilman, 1989), USA | *Fraxinus pennsylvanica Marsh.* | DBH |  |  |  | 0.97  (0.01) |  |  |  |  |  |  |  |
| (Gilman, 1989), USA | *Gleditsia triacanthos var. Inermis L. C.K. Schneid.* | DBH |  |  |  | 0.79  (0.05) |  |  |  |  |  |  |  |
| (Gilman, 1989), USA | *Magnolia grandiflora L.* | DBH |  |  |  | 0.99  (0.01) |  |  |  |  |  |  |  |
| (Gerhold & Johnson, 2003), USA | *Malus 'Harvest Gold'®* | DBH |  | 0.5477  (0.0001) |  | 0.8485  (< 0.05) |  |  |  |  |  |  |  |
| (Gerhold & Johnson, 2003), USA | *Malus 'Professor Sprenger'* | DBH |  | 0.5477  (0.0001) |  | 0.8485  (< 0.05) |  |  |  |  |  |  |  |
| (Peltola et al., 2000), Finnland | *Picea abies (Karst) K.* | DBH |  |  | 0.932  (< 0.01) |  |  |  |  |  |  |  |  |
| (Stokes et al., 2007), France | *Picea alba Mill.* | DBH |  |  |  |  |  |  |  |  |  | 0.8944  (< 0.017) |  |
| (Smith, 1964), USA | *Pinus contorta Bol.* | DBH |  |  |  | 0.694  (< 0.05) |  |  |  |  |  |  |  |
| (Smith, 1964), USA | *Pinus contorta Bol.* | DBH |  |  |  | 0.458  (< 0.05) |  |  |  |  |  |  |  |
| (Smith, 1964), USA | *Pinus contorta Bol.* | DBH |  |  |  | 0.490  (< 0.05) |  |  |  |  |  |  |  |
| (Danjon et al., 2005), France | *Pinus pinaster Ait.* | DBH |  |  |  |  |  |  |  |  | 0.94  (<0.0001) |  |  |
| (Achim & Nicoll, 2009), USA | *Picea sitchensis (Bong.) Carr.* | DBH |  |  |  |  |  |  |  | 0.6856  (0.005) |  |  |  |
| (Peltola et al., 2000), Finnland | *Pinus sylvestris L.* | DBH |  |  | 0.896  (< 0.01) |  |  |  |  |  |  |  |  |
| (Gilman, 1989), USA | *Populus × generosa* | DBH |  |  |  | 0.83  (0.05) |  |  |  |  |  |  |  |
| (Smith, 1964), USA | *Pseudotsuga menziesii (Mirb)* | DBH |  |  |  | 0.593  (0.01) |  |  |  |  |  |  |  |
| (Smith, 1964), USA | *Pseudotsuga menziesii (Mirb)* | DBH |  |  |  | 0.680  (< 0.05) |  |  |  |  |  |  |  |
| (Gerhold & Johnson, 2003), USA | *Pyrus calleryana 'Autumn Blaze'* | DBH |  | 0.5477  (0.0001) |  |  |  |  |  |  |  |  |  |
| (Gerhold & Johnson, 2003), USA | *Pyrus calleryana 'Chanticleer'®* | DBH |  | 0.5477  (0.0001) |  |  |  |  |  |  |  |  |  |
| (Gilman, 1989), USA | *Quercus virginiana Mill.* | DBH |  |  |  | 0.41  (> 0.05) |  |  |  |  |  |  |  |
| (Gerhold & Johnson, 2003), USA | *Syringa reticulata 'Ivory Silk'* | DBH |  | 0.5477  (0.0001) |  | 0.8832  (< 0.05) |  |  |  |  |  |  |  |
| (Gerhold & Johnson, 2003), USA | *Syringa reticulata 'Ivory Silk'* | DBH |  | 0.5477  (0.0001) |  | 0.8832  (< 0.05) |  |  |  |  |  |  |  |
| (Smith, 1964), USA | *Thuja plicata Donn.* | DBH |  |  |  | 0.600  (0.01) |  |  |  |  |  |  |  |
| (Smith, 1964), USA | *Tsuga heterophylla (Raf.) Sarg.* | DBH |  |  |  | 0.714  (0.01) |  |  |  |  |  |  |  |
| (Lundström, Jonsson, et al., 2007), Switzerland | *Picea abies (Karst.) Mill* | DBH High elevation site | 0.9644  (< 0.001) |  |  |  | 0.4899  (< 0.001) | 0.5196  (< 0.001) |  |  |  |  | 0.5477  (< 0.001) |
| (Lundström, Jonsson, et al., 2007), Switzerland | *Picea abies (Karst.) Mill* | DBH Low elevation site | 0.9695  (< 0.001) |  |  |  | 0.3606  (< 0.001) | 0.4690  (< 0.001) |  |  |  |  | 0.4  (< 0.001) |
| (Lundström, Jonas, et al., 2007), Switzerland | *Abies alba Mill* | DBH² |  |  |  |  |  |  |  |  |  | 0.8000  (< 0.001) |  |
| (Lundström, Jonas, et al., 2007), Switzerland | *Picea abies*  *L. Karst, Abies alba Mill, Pinus*  *sylvestris L.* | DBH² |  |  |  |  |  |  |  |  |  | 0.8426  (< 0.001) |  |
| (Stokes et al., 2007), France | *Fagus sylvatica L.* | DBH² |  |  |  |  |  |  |  |  |  | 0.8426  (< 0.016) |  |
| (Lundström, Jonas, et al., 2007), Switzerland | *Picea abies L. Karst* | DBH² |  |  |  |  |  |  |  |  |  | 0.8544  (< 0.001) |  |
| (Stokes et al., 2007), France | *Picea alba Mill.* | DBH² |  |  |  |  |  |  |  |  |  | 0.9165  (< 0.010) |  |
| (Lundström, Jonas, et al., 2007), Switzerland | *Pinus sylvestris L.* | DBH² |  |  |  |  |  |  |  |  |  | -0.7141  (< 0.05) |  |
| (Lundström, Jonas, et al., 2007), Switzerland | *Abies alba Mill* | DBH²H | 0.8426  (< 0.001) |  |  |  |  |  |  |  |  |  |  |
| (Lundström, Jonas, et al., 2007), Switzerand | *Picea abies*  *L. Karst, Abies alba Mill, Pinus*  *sylvestris L.* | DBH²H | 0.8718  (< 0.001) |  |  |  |  |  |  |  |  |  |  |
| (Lundström, Jonas, et al., 2007), Switzerland | *Picea abies L. Karst.* | DBH²H | 0.8718  (< 0.001) |  |  |  |  |  |  |  |  |  |  |
| (Lundström, Jonas, et al., 2007), Switzerland | *Pinus sylvestris L.* | DBH²H | -0.3742  (< 0.05) |  |  |  |  |  |  |  |  |  |  |
| (Lundström, Jonsson, et al., 2007), Switzerand | *Picea abies (Karst.) Mill* | DBH²xH | 0.8944  (<0.001) |  |  |  |  |  |  |  |  |  |  |
| (Lundström, Jonsson, et al., 2007), Switzerland | *Picea abies (Karst.) Mill* | DBH²xH low elevation site | 0.9487  (< 0.001) |  |  |  |  |  |  |  |  |  |  |
| (Peltola et al., 2000), Finnland | *Pinus sylvestris L.* | DBH²xH |  |  | 0.9834  (< 0.0001) |  |  |  |  |  |  |  |  |
| (Peltola et al., 2000), Finnland | *Picea abies L. Karst.* | DBH²xH |  |  | 0.9726  (< 0.0001) |  |  |  |  |  |  |  |  |
| (Peltola et al., 2000), Finnland | *Betula spp.* | DBH²xH |  |  | 0.9576  (< 0.0001) |  |  |  |  |  |  |  |  |
| (Peltola et al., 2000), Finnland | *Pinus sylvestris L.* | Height |  |  | 0.752  (< 0.01) |  |  |  |  |  |  |  |  |
| (Peltola et al., 2000), Finnland | *Picea abies L. Karst.* | Height |  |  | 0.634  (< 0.01) |  |  |  |  |  |  |  |  |
| (Peltola et al., 2000), Finnland | *Betula spp.* | Height |  |  | 0.665  (> 0.05) |  |  |  |  |  |  |  |  |
| (Smith, 1964), USA | *Alnus rubra Bong.* | Height |  |  |  | 0.331  (> 0.05) |  |  |  |  |  |  |  |
| (Smith, 1964), USA | *Pinus contorta Bol.* | Height |  |  |  | 0.701  (< 0.05) |  |  |  |  |  |  |  |
| (Smith, 1964), USA | *Pinus contorta Bol.* | Height |  |  |  | 0.450  (< 0.05) |  |  |  |  |  |  |  |
| (Smith, 1964), USA | *Pseudotsuga menziesii (Mirb)* | Height |  |  |  | 0.356  (0.01) |  |  |  |  |  |  |  |
| (Smith, 1964), USA | *Pseudotsuga menziesii (Mirb)* | Height |  |  |  | 0.616  (< 0.05) |  |  |  |  |  |  |  |
| (Smith, 1964), USA | *Thuja plicata Donn.* | Height |  |  |  | 0.375  (0.01) |  |  |  |  |  |  |  |
| (Smith, 1964), USA | *Tsuga heterophylla (Raf.) Sarg.* | Height |  |  |  | 0.498  (0.01) |  |  |  |  |  |  |  |
| (Stokes et al., 2007), France | *Fagus sylvatica* | HxDBH² m³ |  |  |  |  |  |  |  |  |  | 0.8000  (< 0.030) |  |
| (Stokes et al., 2007), France | *Picea alba Mill.* | HxDBH² m³ |  |  |  |  |  |  |  |  |  | 0.9381  (< 0.005) |  |
| (Peltola et al., 2000), France | *Pinus sylvestris L.* | H/DBH |  |  | 0.818  (< 0.01) |  |  |  |  |  |  |  |  |
| (Peltola et al., 2000), France | *Picea abies L. Karst.* | H/DBH |  |  | 0.362  (> 0.05) |  |  |  |  |  |  |  |  |
| (Peltola et al., 2000), France | *Betula spp.* | H/DBH |  |  | 0.368  (> 0.05) |  |  |  |  |  |  |  |  |
| (Smith, 1964), USA | *Alnus rubra Bong.* | Longest branch |  |  |  | 0.377  (0.05) |  |  |  |  |  |  |  |
| (Smith, 1964), USA | *Pseudotsuga menziesii (Mirb)* | Longest branch |  |  |  | 0.534  (0.01) |  |  |  |  |  |  |  |
| (Smith, 1964), USA | *Thuja plicata Donn.* | Longest branch |  |  |  | 0.544  (0.01) |  |  |  |  |  |  |  |
| (Smith, 1964), USA | *Tsuga heterophylla (Raf.) Sarg.* | Longest branch |  |  |  | 0.652  (0.01) |  |  |  |  |  |  |  |
| (Nicoll & Ray, 1996), UK | *Picea sitchensis (Bong.) Carr.* | Mean winter table depth |  |  |  | (< 0.001) |  |  |  |  |  | 0.4472  (< 0.05) |  |
| (Danjon et al., 2005), France | *Pinus pinaster Ait.* | Root grafts relative surface |  |  |  |  |  |  |  |  | 0.53  (<0.05) |  |  |
| (Danjon et al., 2005), France | *Pinus pinaster Ait.* | Root length |  |  |  |  |  |  |  |  | 0.89  (<0.0001) |  |  |
| (Danjon et al., 2005), France | *Pinus pinaster Ait.* | Root maximal radial distance |  |  |  |  |  |  |  |  | 0.67  (<0.01) |  |  |
| (Danjon et al., 2005), France | *Pinus pinaster Ait.* | Root numer |  |  |  |  |  |  |  |  | 0.94  (<0.0001) |  |  |
| (Kamimura et al., 2012), Japan | *Chamaecyparis obtuse*  *(Sieb. Et Zucc.) Endl.* | Root plate area |  |  |  |  |  |  |  |  | 0.789  (0.011) |  |  |
| (Kamimura et al., 2012), Japan | *Chamaecyparis obtuse*  *(Sieb. Et Zucc.) Endl.* | Root plate volume |  |  |  |  |  |  |  |  | 0.691  (0.039) |  |  |
| (Peltola et al., 2000), Finnland | *Pinus sylvestris L.* | Root-soil plate depth |  |  | 0.693  (< 0.01) |  |  |  |  |  |  |  |  |
| (Peltola et al., 2000), Finnland | *Picea abies L. Karst.* | Root-soil plate depth |  |  | 0.495  (< 0.05) |  |  |  |  |  |  |  |  |
| (Peltola et al., 2000), Finnland | *Betula spp.* | Root-soil plate depth |  |  | 0.718  (< 0.05) |  |  |  |  |  |  |  |  |
| (Peltola et al., 2000), Finnland | *Pinus sylvestris L.* | Root-soil plate radius |  |  | 0.476  (< 0.05) |  |  |  |  |  |  |  |  |
| (Peltola et al., 2000), Finnland | *Picea abies L. Karst.* | Root-soil plate radius |  |  | 0.606  (> 0.05) |  |  |  |  |  |  |  |  |
| (Peltola et al., 2000), Finnland | *Betula spp.* | Root-soil plate radius |  |  | 0.661  (> 0.05) |  |  |  |  |  |  |  |  |
| (Danjon et al., 2005), France | *Pinus pinaster Ait.* | Root volume |  |  |  |  |  |  |  |  | 0.88  (<0.0001) |  |  |
| (Faulkner & Malcolm, 1972), UK | *Calluna vulgaris L.* | Rootable volume |  |  |  |  |  |  |  |  |  | 0.72111  (< 0.01) |  |
| (Mueller & Cline, 1959), USA | *Prunus serotina Schur., Ulmus americana L.; Acer sacharum Marsh.; Liriodendron tulipifera L.* | Soil wetness calcareous till |  | -0.422  (> 0.05) |  | 0.519  (< 0.05) |  |  | 0.313  (> 0.05) |  |  |  |  |
| (Mueller & Cline, 1959), USA | *Prunus serotina Schur., Ulmus americana L.; Acer sacharum Marsh.; Liriodendron tulipifera L.* | Soil wetness fragipan |  | -0.907  (< 0.01) |  | -0.112  (> 0.05) |  |  | -0.509  (< 0.05) |  |  |  |  |
| (Danjon et al., 2005), France | *Pinus pinaster Ait.* | Stem height |  |  |  |  |  |  |  |  | 0.61  (<0.05) |  |  |
| (Nicoll & Ray, 1996), UK | *Picea sitchensis (Bong.) Carr.* | Stem mass |  | < 0.001 |  |  |  |  |  |  |  |  |  |
| (Lundström, Jonsson, et al., 2007), Switzerland | *Picea abies (Karst.) Mill* | Stem mass (kg) high elevation site | 0.9592  (< 0.001) |  |  |  |  |  |  |  |  |  |  |
| (Lundström, Jonsson, et al., 2007), Switzerland | *Picea abies (Karst.) Mill* | Stem mass (kg) low elevation site | 0.9644  (< 0.001) |  |  |  |  |  |  |  |  |  |  |
| (Lundström, Jonsson, et al., 2007), Switzerland | *Picea abies (Karst.) Mill* | Stem volume high elevation site | 0.9592  (<0.001) |  |  |  |  |  |  |  |  |  |  |
| (Lundström, Jonsson, et al., 2007), Switzerland | *Picea abies (Karst.) Mill* | Stem volume low elevation site | 0.9644  (< 0.001) |  |  |  |  |  |  |  |  |  |  |
| (Faulkner & Malcolm, 1972), UK | *Calluna vulgaris L.* | Stem weight |  |  |  |  |  |  |  |  |  | 0.9381  (< 0.01) |  |
| (Peltola et al., 2000), Finnland | *Pinus sylvestris L.* | Stem weight |  |  | 0.972  (< 0.01) |  |  |  |  |  |  |  |  |
| (Peltola et al., 2000), Finnland | *Picea abies L. Karst.* | Stem weight |  |  | 0.974  (< 0.01) |  |  |  |  |  |  |  |  |
| (Peltola et al., 2000), Finnland | *Betula spp.* | Stem weight |  |  | 0.790  (< 0.05) |  |  |  |  |  |  |  |  |
| (Danjon et al., 2005), France | *Pinus pinaster Ait.* | Stump volume |  |  |  |  |  |  |  |  | 0.78  (<0.001) |  |  |
| (Stokes et al., 2007), France | *Fagus sylvatica L.* | Total biomass (stem+crown) (kg) |  |  |  |  |  |  |  |  |  | 0.8718  (< 0.011) |  |
| (Stokes et al., 2007), France | *Picea alba Mill.* | Total biomass (stem+crown) (kg) |  |  |  |  |  |  |  |  |  | 0.9110  (< 0.012) |  |
| (Lundström, Jonas, et al., 2007), Switzerland | *Abies alba Mill* | Total mass tree (kg) |  |  |  |  |  |  |  |  |  | 0.9055  (< 0.001) |  |
| (Lundström, Jonas, et al., 2007), Switzerland | *Abies alba Mill, Picea abies L. Karst, Pinus sylvestris L.* | Total mass tree (kg) |  |  |  |  |  |  |  |  |  | 0.9000  (< 0.001) |  |
| (Lundström, Jonas, Stöckli, & Ammann, 2007), Switzerland | *Picea abies L. Karst* | Total mass tree (kg) |  |  |  |  |  |  |  |  |  | 0.8718  (< 0.001) |  |
| (Lundström, Jonas, et al., 2007), Switzerland | *Pinus sylvestris L.* | Total mass tree (kg) |  |  |  |  |  |  |  |  |  | 0.9899  (< 0.001) |  |
| (Lundström, Jonas, et al., 2007), Switzerland | *Abies alba Mill* | Total stem mass (kg) |  |  |  |  |  |  |  |  |  | 0.8660  (< 0.001) |  |
| (Lundström, Jonas, et al., 2007), Switzerland | *Abies alba Mill, Picea abies L. Karst, Pinus sylvestris L.* | Total stem mass (kg) |  |  |  |  |  |  |  |  |  | 0.8718  (< 0.001) |  |
| (Lundström, Jonas, et al., 2007), Switzerland | *Picea abies L. Karst* | Total stem mass (kg) |  |  |  |  |  |  |  |  |  | 0.8544  (< 0.001) |  |
| (Lundström, Jonas, et al., 2007), Switzerland | *Pinus sylvestris L.* | Total stem mass (kg) |  |  |  |  |  |  |  |  |  | 0.1000  (< 0.05) |  |
| (Mueller & Cline, 1959), USA | *Prunus serotina Schur., Ulmus americana L., Acer sacharum Marsh.; Liriodendron tulipifera L.* | Tree diameter |  | 0.166  (> 0.05) |  | 0.633  (< 0.01) |  |  | 0.637  (< 0.01) |  |  |  |  |
| (Mueller & Cline, 1959), USA | *Prunus serotina Schur., Ulmus americana L., Acer sacharum Marsh.; Liriodendron tulipifera L.* | Tree diameter |  | 0.296  (> 0.05) |  | 0.555  (< 0.05) |  |  | 0.502  (< 0.05) |  |  |  |  |
| (Lundström, Jonsson, et al., 2007), Switzerland | *Picea abies (Karst.) Mill* | Tree mass (kg) high elevation site | 0.9592  (< 0.001) |  |  |  |  |  |  |  |  |  |  |
| (Lundström, Jonsson, et al., 2007), Switzerland | *Picea abies (Karst.) Mill* | Tree mass (kg) low elevation site | 0.9644  (< 0.001) |  |  |  |  |  |  |  |  |  |  |
| (Štofko & Kodrík, 2008), Czech Republic | *Picea abies L. Karst.* | Width root plate undamaged |  |  |  |  | 0.068  (> 0.05) | 0.576  (< 0.05) |  |  |  |  |  |
| (Štofko & Kodrík, 2008), Czech Republic | *Picea abies L. Karst.* | Width root plate undamaged |  |  |  |  | 0.021  (> 0.05) |  |  |  |  |  |  |
| (Štofko & Kodrík, 2008), Czech Republic | *Picea abies L. Karst.* | Width root plate wind throw |  |  |  |  | 0.156  (> 0.05) | 0.337  (> 0.05) |  |  |  |  |  |
| (Štofko & Kodrík, 2008), Czech Republic | *Picea abies L. Karst.* | Width root plate wind throw |  |  |  |  | 0.151  (> 0.05) |  |  |  |  |  |  |
| (Crook & Ennos, 1996), UK | *Larix europea L. x Larix japonica A.Murray bis* | Windward laterals area | 0.6928  (<0.001) |  |  |  |  |  |  |  |  |  |  |
| (Crook & Ennos, 1996), UK | *Larix europea L. x Larix japonica A.Murray bis* | Windward sinkers | 0.7681  (<0.000) |  |  |  |  |  |  |  |  |  |  |
| Total number of studies | | 46 | 16 | 28 | 26 | 68 | 6 | 4 | 6 | 1 | 9 | 25 | 2 |
| Author(year) | Species | Variables | Anchorage strength | Root depth | Root failure | Root spread® | Root soil plate depth | Root soil plate length | Root volume | Soil type | Stem volume | Turning moment | vertical radius root plate (width) |

1. DBH = diameter breast height, DBH² = diameter breast height squared, DBH²H = diameter breast height squared times height, H/DBH = height divided by diameter breast height.
