# Supplementary material for: Understanding tree failure—A systematic review and meta-analysis
Source: PLoS One. 2021 Feb 16;16(2):e0246805. doi: 10.1371/journal.pone.0246805 (PMC7886209; doi:10.1371/journal.pone.0246805)
Supplement: S3 Table — (DOCX) [file pone.0246805.s004.docx]

S3 Table: Overview of all effect sizes (correlations) mentioned in studies of branch failure (Note: factors can be labelled horizontal and vertical)

| Branch failure | | Variable | Aspect ratio | Attachment angle | Bifurcation strength | Breaking stress | Crown length | Crown width | DBH | DBH³ | H/D (Slenderness) | Height | MOE | MOR | Stem volume | Wood density |
| --- | --- | --- | --- | --- | --- | --- | --- | --- | --- | --- | --- | --- | --- | --- | --- | --- |
| Author (year) | Species |  |  |  |  |  |  |  |  |  |  |  |  |  |  |  |
| (Eisner, Gilman, & Grabosky, 2002), USA | *Acer rubrum L.* | Area of discolored wood | 0.8775  (< 0.01) |  |  |  |  |  |  |  |  |  |  |  |  |  |
| (Eisner et al., 2002), USA | *Quercus virginiana L.* | Area of discolored wood | 0.7000  (< 0.01) |  |  |  |  |  |  |  |  |  |  |  |  |  |
| (Kane, Farrell, Zedaker, Lofersky, & Smith, 2008), USA | *Acer rubrum L.* | Aspect ratio |  |  |  | 0.7483  (< 0.01) |  |  |  |  |  |  |  |  |  |  |
| (Gilman, 2003), USA | *Acer rubrum L.* | Aspect ratio |  |  |  | 0.7483  (< 0.001) |  |  |  |  |  |  |  |  |  |  |
| (Kane et al., 2008), USA | *Pyrus calleryana Decne* | Aspect ratio |  |  |  | 0.7681  (< 0.01) |  |  |  |  |  |  |  |  |  |  |
| (Kane, 2007), USA | *Pyrus calleryana var. ‘Bradford’* | Aspect ratio |  |  |  | 0.4359  (0.0242) |  |  |  |  |  |  |  |  |  |  |
| (Kane et al., 2008), USA | *Quercus acutissima Carruthers* | Aspect ratio |  |  |  | 0.7483  (< 0.01) |  |  |  |  |  |  |  |  |  |  |
| (Kane et al., 2008), USA | *Pyrus calleryana Decne* | Attachment angle |  |  | 0.4796  (< 0.01) | 0.4123  (< 0.01) |  |  |  |  |  |  |  |  |  |  |
| (Kane et al., 2008), USA | *Pyrus calleryana Decne* | Attachment angle |  |  |  | 0.5916  (< 0.01) |  |  |  |  |  |  |  |  |  |  |
| (Kane, 2007), USA | *Pyrus calleryana var. ‘Bradford’* | Attachment angle |  |  | 0.6481  (0.006) |  |  |  |  |  |  |  |  |  |  |  |
| (Kane et al., 2008), USA | *Quercus acutissima Carruthers* | Attachment angle |  |  |  | 0.6856  (< 0.01) |  |  |  |  |  |  |  |  |  |  |
| (Kane et al., 2008), USA | *Acer rubrum L.* | Attachment length |  |  |  | 0.3317  (< 0.01) |  |  |  |  |  |  |  |  |  |  |
| (Kane et al., 2008), USA | *Pyrus calleryana Decne* | Attachment length |  |  |  | 0.6403  (< 0.01) |  |  |  |  |  |  |  |  |  |  |
| (Kane et al., 2008), USA | *Quercus acutissima Carruthers* | Attachment length |  |  |  | 0.3873  (< 0.01) |  |  |  |  |  |  |  |  |  |  |
| (Kane & Clouston, 2008), USA | *Acer sp.* | Bending moment at failure point |  |  |  |  | 0.2449  (0.8751) | 0.3  (0.6527) | 0.5099  (0.4334) | 0.5292  (0.0617) | 0.2  (0.0092) | 0.1414  (0.2936) |  | 0.1  (0.043) | 0.5385  (0.0085) |  |
| (Kane et al., 2008), USA | *Acer rubrum L.* | Branch angle |  |  |  | 0.4123  (< 0.01) |  |  |  |  |  |  |  |  |  |  |
| (Kane et al., 2008), USA | *Pyrus calleryana Decne* | Branch angle |  |  |  | 0.5916  (< 0.01) |  |  |  |  |  |  |  |  |  |  |
| (Kane et al., 2008), USA | *Quercus acutissima Carruthers* | Branch angle |  |  |  | 0.6856  (< 0.01) |  |  |  |  |  |  |  |  |  |  |
| (Kane et al., 2008), USA | *Acer rubrum L.* | Branch bark ridge angle |  |  |  | 0.3873  (< 0.01) |  |  |  |  |  |  |  |  |  |  |
| (Kane et al., 2008), USA | *Pyrus calleryana Decne* | Branch bark ridge angle |  |  |  | 0.5568  (< 0.01) |  |  |  |  |  |  |  |  |  |  |
| (Kane et al., 2008), USA | *Quercus acutissima Carruthers* | Branch bark ridge angle |  |  |  | 0.6  (< 0.01) |  |  |  |  |  |  |  |  |  |  |
| (Kane et al., 2008), USA | *Acer rubrum L.* | Branch bark ridge length |  |  |  | 0.3317  (< 0.01) |  |  |  |  |  |  |  |  |  |  |
| (Kane et al., 2008), USA | *Pyrus calleryana Decne* | Branch bark ridge length |  |  |  | 0.6164  (< 0.01) |  |  |  |  |  |  |  |  |  |  |
| (Kane et al., 2008), USA | *Quercus acutissima Carruthers* | Branch bark ridge length |  |  |  | 0.3606  (< 0.01) |  |  |  |  |  |  |  |  |  |  |
| (Spatz, Brüchert, & Pfisterer, 2007), Germany | *Pseudotsuga menziesii* | Branch diameter |  |  |  |  |  |  |  |  |  |  | 0.9279  (< 0.05) |  |  |  |
| (Kane et al., 2008), USA | *Acer rubrum L.* | Branch diameter/attachment width |  |  |  | 0.7141  (< 0.01) |  |  |  |  |  |  |  |  |  |  |
| (Kane et al., 2008), USA | *Pyrus calleryana Decne* | Branch diameter/attachment width |  |  |  | 0.8062  (< 0.01) |  |  |  |  |  |  |  |  |  |  |
| (Kane et al., 2008), USA | *Quercus acutissima Carruthers* | Branch diameter/attachment width |  |  |  | 0.7483  (< 0.01) |  |  |  |  |  |  |  |  |  |  |
| (Spatz et al., 2007), Germany | *Pseudotsuga menziesii* | Branch mass |  |  |  |  |  |  |  |  |  |  |  |  | 0.0665  (<0.05) | 0.7510  (< 0.05) |
| (Kane et al., 2008), USA | *Acer rubrum L.* | Breaking stress |  |  |  |  |  |  |  |  |  |  |  |  |  | 0.1  (< 0.01) |
| (Kane et al., 2008), USA | *Pyrus calleryana Decne* | Breaking stress |  |  |  |  |  |  |  |  |  |  |  |  |  | 0.1  (< 0.01) |
| (Kane et al., 2008), USA | *Quercus acutissima Carruthers* | Breaking stress |  |  |  |  |  |  |  |  |  |  |  |  |  | 0.4243  (< 0.01) |
| (Kane & Finn, 2014), USA | 10 species¹ | DBH |  | 0.00  (> 0.05) |  |  |  |  |  |  |  |  |  |  |  |  |
| (Dahle et al., 2006), USA | *Acer platanoides L.* | Diameter at point of failure branch |  |  |  |  |  |  |  |  |  |  |  | 0.5657  (0.06) |  |  |
| (Buckley, Slater, & Ennos, 2015), UK | *Corylus avelana L.* | Diameter bifurcation |  |  |  |  |  |  |  |  |  |  |  | 0.4838  (< 0.001) |  |  |
| (Dahle et al., 2006), USA | *Acer platanoides L.* | Diameter decay at failure/diameter watersprout at failure |  |  |  |  |  |  |  |  |  |  |  | 0.7746  (0.008) |  |  |
| (Smiley, 2003), USA | *Acer rubrum L.* | Force to break bark excluded x DBH |  |  |  |  |  |  | 0.8718  (< 0.05) |  |  |  |  |  |  |  |
| (Smiley, 2003), USA | *Acer rubrum L.* | Force to break bark included x DBH |  |  |  |  |  |  | 0.9592  (< 0.05) |  |  |  |  |  |  |  |
| (Kane et al., 2008), USA | *Acer rubrum L.* | Rough branch diameter/attachment width |  |  |  | 0.6083  (< 0.01) |  |  |  |  |  |  |  |  |  |  |
| (Kane et al., 2008), USA | *Pyrus calleryana Decne* | Rough branch diameter/attachment width |  |  |  | 0.6325  (< 0.01) |  |  |  |  |  |  |  |  |  |  |
| (Kane et al., 2008), USA | *Quercus acutissima Carruthers* | Rough branch diameter/attachment width |  |  |  | 0.6164  (< 0.01) |  |  |  |  |  |  |  |  |  |  |
| (Buckley et al., 2015), UK | *Corylus avellana L.* | Shape of parent stem below bifurcation |  | 0.363  (< 0.001) |  |  |  |  |  |  |  |  |  |  |  |  |
| (Buckley et al., 2015), UK | *Corylus avellana L.* | Shape of arising branch from the bifurcation |  | 0.235  (< 0.001) |  |  |  |  |  |  |  |  |  |  |  |  |
| (Kane & Finn, 2014), USA | 10 species¹ | Species (typological or morphological) |  | 0.00  (0.096) |  |  |  |  |  |  |  |  |  |  |  |  |
| (Kane & Clouston, 2008), USA | *Acer sp.* | Stress at failure point |  |  |  |  | 0.2828  (0.0458) | 0.2828  (0.0458) | 0.4123  (0.0469) | 0.3873  (0.0584) | 0.1414  (0.0667) | 0.00  (0.7553) |  | 0.00  (0.8369) | 0.2828  (0.0001) |  |
| (Kane et al., 2008), USA | *Acer rubrum L.* | Trunk diameter/attachment width |  |  |  | 0.6  (< 0.01) |  |  |  |  |  |  |  |  |  |  |
| (Kane et al., 2008), USA | *Pyrus calleryana Decne* | Trunk diameter/attachment width |  |  |  | 0.7280  (< 0.01) |  |  |  |  |  |  |  |  |  |  |
| (Kane et al., 2008), USA | *Quercus acutissima Carruthers* | Trunk diameter/attachment width |  |  |  | 0.7  (< 0.01) |  |  |  |  |  |  |  |  |  |  |
| Total number of studies | | 22 | 2 | 4 | 2 | 16 | 2 | 2 | 4 | 3 | 2 | 2 | 1 | 5 | 3 | 1 |
| Author (year) | Species | Variable | Aspect ratio | Attachment angle | Bifurcation strength | Breaking stress | Crown length | Crown width | DBH | DBH³ | H/D (Slenderness) | Height | MOE | MOR | Stem volume | Wood density |

1. *Platanus x acerifolia (Air.) Willd.; Quercus palustris; Quercus rubra L.; Fraxinus pennsylvanica Marsh.; Pinus strobus L.; Gleditsia triacanthos L.; Acer rubrum L.; Pinus resinosa Ait.; Ulmus americana L. ‘American Liberty’; Tilia cordata Mill.*
2. DBH = diameter breast height, DBH³ = diameter breast height cubed, H/D = height divided by diameter, MOE = modulus of elasticity, MOR = modulus of rupture.
