# Supplementary material for: Understanding tree failure—A systematic review and meta-analysis
Source: PLoS One. 2021 Feb 16;16(2):e0246805. doi: 10.1371/journal.pone.0246805 (PMC7886209; doi:10.1371/journal.pone.0246805)
Supplement: S1 Text — (DOCX) [file pone.0246805.s005.docx]

S1 Text Quality Criteria and Assessment

The quality of each included study was determined across seven criteria: study design, method quality, description of the study population (forest, urban trees, singe tree), data collection (prospective or retrospective), inclusion criteria, exclusion criteria, full description of the relation to tree failure. All studies included in the meta-analysis matched all quality criteria

Table 1: Quality assessment of included studies

| Criteria | 0 point | 1 point |
| --- | --- | --- |
| Study design | Not deductible | All study designs mentioned |
| Analysis | No statistics | Descriptive statistics and/or statistical analysis |
| Description study population | No species specified, no characteristics (habitat) | Full description |
| Data collection | Prospective or no description | Retrospective |
| Inclusion criteria | No description | Full description |
| Exclusion criteria | No description | Full description |
| Relation factor to tree failure | No description | Full description |
