# Supplementary material for: Understanding tree failure—A systematic review and meta-analysis
Source: PLoS One. 2021 Feb 16;16(2):e0246805. doi: 10.1371/journal.pone.0246805 (PMC7886209; doi:10.1371/journal.pone.0246805)
Supplement: S2 Text — (DOCX) [file pone.0246805.s006.docx]

S2 Text: Overview of study characteristics included in the meta-analysis

| Author (year) | Study object | Factors^1^ | Type^2^ |
| --- | --- | --- | --- |
| (Achim, Nicoll, Mochan, & Gardiner, 2003), UK | 36 *Picea sitchensis (Bong.) Carr* | Stem weight | S |
| (Achim, Ruel, Gardiner, Laflamme, & Meunier, 2005), Canada | 160 *Abies balsamea (L.) Mill* | Stem mass | S |
| (Achim, Ruel, Gardiner, 2005) Canada | 40 *Abies balsamea (L.) Mill* | Stem weight | S |
| (Cannon, Barrett & Peterson, 2015), USA | 36 *Pinus taeda L.*  23 *Liriodendron tulipifera L.*  5 *Acer floridanum (Chapm.) Pax*  1 *Cornus florida L.*  1 *Fraxinus Americana L.*  1 *Liquidambar styraciflua L.*  1 *Quercus alba L.*  1 *Quercus rubra L.* | DBH, DBH², DBH³, DBH²H, Stem mass, Tree mass | S |
| (Cucchi, Meredieu, Stokes, Berthier, Bert, Najar, Denis,  Lastennet, 2004), France | 79 *Pinus pinaster Ait*. | DBH²H, DBH³, Stem weight, DBH, Crown biomass, Root-soil plate volume, Height, Root-soil plate depth, Stem taper | S |
| (Elie & Ruel, 2005), France | 30 *Pinus banksiana Lamb.*  55 *Picea mariana (Mill.) BSP* | Stem mass | S |
| (Foster, 1988), USA | 115 tree plots | Age, Height | S |
| (Fraser, 1962), UK | 56 *Picea sitchensis (Bong.) Carr.* | Stem weight | S |
| (Fredericksen, Hedden, & Williams, 1993), USA | 40 *Pinus taeda L.* | Tree weight, Stem volume, Stem DBH³, Tree height³, Taper coefficient | S |
| (Gardiner, Stacey, Belcher, & Wood, 1997), UK | 205 *Picea sitchensis (Bong.) Carr.* | Stem weight, DBH³ | S |
| (Hale, Gardiner, Wellpott, Nicoll, & Achim, 2012), UK | 26 *Picea sitchensis (Bong.) Carr.*  7 *Larix decidua Mill* | Wind speed | S |
| (Hedden, Fredericksen, & Williams, 1995), USA | 40 *Pinus taeda L.* | DBH, Height, Taper coefficient, Center of gravity  Total tree green weight, Crown green weight, Crown weight : stem weight, DBH²H, Deflection at midstem before failure | S |
| (Kamimura, Kitagawa, Saito & Mizunaga, 2012), Japan | 9 Chamaecyparis obtuse (Sieb. Et Zucc.) Endl. | DBH, DBH²H, Tree weight, Root plate area, Root plate volume, Water content below the root plate, Water content inside the root plate | S, R |
| (Kane, 2014), USA | 55 *Quercus rubra L.* | DBH, Crown width | S, R |
| (Kane, Modarres-Sadeghi, James, & Reiland, 2014), USA | 8 *Acer saccharum Marsh.* | Crown width | S |
| (Lundström, Jonas, Stöckli, Amman, 2007), Switzerland | 38 *Picea abies L. Karst*  19 *Abies alba Mill*  3 *Pinus sylvestris L.* | Tree mass, Stem mass, DBH²H, DBH² | R |
| (Lundström, Jonsson, & Kalberer, 2007), Switzerland | 66 *Picea abies L. Karst.* | Tree mass, Stem mass, Stem volume, DBH²H | R |
| (Moore, 2000), New Zealand | 164 *Pinus radiata D.Don* | DBH, Height, Stem volume, H/DBH, Root plate depth, Root plate width, Root plate width+depth, Volume+H/DBH+width | S, R |
| (Papesch, Moore, & Hawke, 1997), UK | 62 *Pinus radiata D. Don* | DBH, Height, Stem volume | S, R |
| (Peltola, Kellomäki, Hassinen, & Granander, 2000), Finnland | 71 *Pinus sylvestris L.*  33 *Picea abies (L.) Karst.*  11 *Betula spp.* | Stem angle at maximum moment, Crown area, DBH, H/DBH, Height, Root-soil plate depth, Root-soil plate radius, Stem mass | S, R |
| (Peterson & Claassen, 2013), USA | 21 *Populus fremontii Wats.*  39 *Quercus lobata Nee* | DBH, DBH², DBH³, DBH²H, Taper (H/DBH), Tree height, Tree mass | S |
| (Ribeiro, Chambers, Peterson, Trumbore, Magnabosco Marra, Wirth, Cannon, Négron-Juárez, Lima, de Paula, Santos & Higuchi, 2016), Brazil | 9 Eschweilera spp  20 Scleronema mincranthum (Ducke) Ducke | Diameter stump, Stem mass, Crown mass, Above ground biomass, Stem volume, Tree height, DBH, Tree center of mass, Stem fresh wood density, critical turning moment | S |
| (Smith, 1964), Canada | 203 *Pseudotsuga menziesii (Mirb.) Franco*  81 *Tsuga heterophylla (Raf.) Sarg.*  61 *Thuja plicata Donn.*  33 *Alnus rubra Bong.*  84 *Pinus contorta Dougl.*  42 *Pinus ponderosa Laws.*  7 *Picea engelmanii Parry*  23 *Picea glauca (Moench) Voss*  7 *Picea sitchensis (Bong.) Carr.* | DBH, Height, Crown width, Age | R |
| (Stokes, Salin, Dzifa Kokutse, Berthier, Jeannin, Mochan, Dorren, Kokutse, Ghani & Fourcaud, 2007), France | 4 *Fagus sylvatica L.*  2 *Abies alba Mill.*  5 *Picea abies L.* | DBH, DBH², DBH²H, Crown biomass, Total mass | R |

^1^ Factors: DBH = diameter breast height, DBH³ = diameter breast height cubed, DBH²H = diameter breast height squared times height, H/DBH = height divided by diameter breast height

^2^ Failure types: S = stem failure, R = Root failure
