# Supplementary material for: Understanding tree failure—A systematic review and meta-analysis
Source: PLoS One. 2021 Feb 16;16(2):e0246805. doi: 10.1371/journal.pone.0246805 (PMC7886209; doi:10.1371/journal.pone.0246805)
Supplement: S3 Text — (DOCX) [file pone.0246805.s007.docx]

S3 Text: Overview of characteristics of studies in the systematic review

| Reference | | Journal | Region | Study design | Method | Failure due to | Habitat | Failure type |
| --- | --- | --- | --- | --- | --- | --- | --- | --- |
| 1. | (Achim, Nicoll, Mochan, & Gardiner, 2003) | Proceedings International Conference ‘Wind Effects on Trees’ | Leanachan Forest, Fort William, Scotland, UK | Experiment (in the field) | Biomechanical modelling and analysis | Wind | Forest | Stem |
| 2. | (Achim, Ruel, & Gardiner, 2005) | Canadian Journal of Forest Research | Montmorency forest, Laurentian Mountains, Québec, Canada | Experiment (in the field) | Analysis of variance  Regression analysis | Wind | Forest | Stem |
| 3. | (Achim, Ruel, Gardiner, Laflamme, & Meunier, 2005) | Forest Ecology and Management | Montmorency  forest, Laurentian, Québec, Canada | Experiment (in the field) | Biomechanical analysis  Residual maximum likelihood analysis | Wind | Forest | Stem |
| 4. | (Achim & Nicoll, 2009) | Urban Forestry & Urban Greening | Nickerson state park, Barnstable County, Massachusetts, USA | Experiment (in the field) | Soil mechanical analysis  Analysis of variance  Regression analysis | Anchorage strength | Forest | Root |
| 5. | (Adams, 1967) | Journal of Applied Ecology | Kassala Province, Sudan | Case study | Descriptive statistics | Root spread and depth | Forest | Root |
| 6. | (Aldrich, Work, & Lewis, 1935) | Journal of Agricultural Research | Rogue River Valley of Oregon | Case study | Correlation analysis | Root spread and depth | Forest | Root |
| 7. | (Ashton, 1975) | Australian Journal of Botany | Victora, Wallaby Creek. Australia | Case study | Descriptive statistics | Root spread and depth | Forest | Root |
| 8. | (Asner & Goldstein, 1997) | Biotropica | Kokee state park, Kauai, Hawaii, USA | Case study | Biomechanical analysis | Hurricane Iniki | Forest | Stem |
| 9. | (Baker, 1997) | Journal of Experimental Biology | Nottingham University campus, UK | Experiment (in the field) | Biomechanical analysis | Wind | Urban | Stem |
| 10. | (Ballantyne, 1916) | UAES Bulletins. Paper 109 | Utah State University, Utah Agricultural Experimentation Station, St. George | Experiment (in the field) | Descriptive statistics | Root spread and depth | Forest | Root |
| 11. | (Bannan, 1940) | American Journal of Botany | Lake Superior, Thunder Bay, Ontario, USA | Case study | Descriptive statistics | Root spread and depth | Forest | Root |
| 12. | (Batanouny & Abdel Wahab, 1973) | Oecologia | wadi El-Assiuti, Egypt | Case study | Descriptive statistics | Root spread and depth | Desert | Root |
| 13. | (Bergeron, Ruel, Elie, & Mitchell, 2008) | Forestry | Québec, Quebec, Canada | Experiment (in the field) | Descriptive statistics  Regression analysis | Wind | Forest | Stem |
| 14. | (Berndt & Gibbons, 1958) | Research report | Rocky Mountain Forest and Range Experiment Station Fort Collins, Colorado, USA | Case study | Descriptive statistics | Root spread and depth | Forest | Root |
| 15. | (Bishop, 1962) | Ecology | Blue Mountains of Oregon, USA | Case study | Graphical analysis | Root spread and depth | Forest | Root |
| 16. | (Biswell, 1935) | Botanical Gazette | Fayette, Missouri, USA | Case study | Descriptive statistics | Root spread and depth | Forest | Root |
| 17. | (Brüchert, Becker & Speck, 2000) | Forest Ecology and Management | Altglashütten, Black Forest, Germany | Experiment (in the field) | Biomechanical analysis | Wind | Forest | Stem |
| 18. | (Brüchert & Gardiner, 2006) | American Journal of Botany | Kilmichael Forest, Argyll Forest District, western Scotland, UK | Case study | Descriptive statistics  Analysis of variance  Regression analysis | Wind | Forest | Stem |
| 19. | (Buckley, Slater, & Ennos, 2015) | Arboricultural Journal: The International Journal of Urban Forestry | Silverdale, Lancashire, UK | Experiment (laboratory) | Biomechanical analysis  Regression analysis | Wind  Angle of inclination | Forest | Branch |
| 20. | (Cable, 1977) | Journal of range management | Santa Rita Experimental Range, Tucson, Arizona | Case study | Descriptive statistics | Root spread and depth | Forest | Root |
| 21. | (Cannon, Barrett, & Peterson, 2015) | Forest Ecology and Management | Hillsboro, Georgia, USA | Experiment (in the field) | Biomechanical analysis  Regression analysis | Wind | Forest | Stem |
| 22. | (Castro-García, Blanco-Roldán, Gil-Ribes, & Agüera-Vega, 2008) | Trees | Cordoba, Spain | Quasi-experimental study | Biomechanical analysis  Analysis of variance | Wind | Orchard | Stem |
| 23. | (Chiba, 2000) | Forest Ecology and Management | Hita City, Ooita Prefecture, Japan | Experiment (in the field) | Biomechanical analysis  Regression analysis | Wind | Forest | Stem |
| 24. | (Ciftci, Arwade, Kane, & Brena, 2014) | Probabilistic Engineering Mechanics | Belchertown, Amherst, Massachusets, USA | Quasi-experimental study | Biomechanical analysis  Monte Carlo simulation | Wind | Solitary | Stem |
| 25. | (Coile, 1937) | Journal of Forestry | Duke forest, north carolina, USA | Case study | Analysis of variance | Anchorage strength | Forest | Root |
| 26. | (Coutts, 1986) | Forestry | Kershope forest, UK | Experiment (in the field) | Biomechanical analysis | Anchorage strength | Forest | Root |
| 27. | (Coutts, 1986) | Forestry | Roslin, Midlothian, UK | Experiment (in the field) | Soil mechanical analysis  Descriptive statistics | Anchorage strength | Forest | Root |
| 28. | (Crook & Ennos, 1996) | Journal of Experimental Botany | Granada Arboretum, Jodrell Bank, Manchester, UK | Experiment (in the field) | Soil mechanical analysis  Regression analysis | Anchorage strength | Forest | Root |
| 29. | (Cucchi et al., 2004) | Trees | France | Experiment (in the field) | Biomechanical analysis  Regression analysis and GLM | Rooting depth  Wind | Forest | Stem |
| 30. | (Dahle & Grabosky, 2010) | Urban Forestry & Urban Greening | Rutgers Gardens, New Brunswick, New Jersey, USA | Experiment (in the field) | Biomechanical analysis  Descriptive statistics  Analysis of variance | Wind | Urban | Branch |
| 31. | (Dahle et al., 2006) | Arboriculture & Urban Forestry | Tippecanoe County, Indiana, USA | Experiment (in the field) | Biomechanical analysis  Regression analysis | Decay | Forest | Branch |
| 32. | (Danjon, Fourcaud, & Bert, 2005) | New Phytologist | Landes de Gascogne Forest, Bordeaux, France | Case study | Biomechanical analysis  Principal component analysis | Wind  Anchorage strength | Forest | Root |
| 33. | (Davis, 1977) | Hydrology and water resources in Arizona and the Southwest | Nevada, Arizona, USA | Case study | Descriptive statistics | Root spread and depth | Forest | Root |
| 34. | (Day, 1944) | The American Midland Naturalist | Sault Ste. Marie, Michigan, USA | Case study | Correlation analysis | Root spread and depth | Forest | Root |
| 35. | (Dorren & Berger, 2006) | Tree physiology | Forêt Communale de Vaujany, France | Experiment (in the field) | Biomechanical analysis | Rockfall | Forest | Stem |
| 36. | (Eames & Cox, 1945) | American Journal of Botany | Norwich, New York, USA | Case study | Descriptive statistics | Graft union | Urban | Stem |
| 37. | (Edberg & Berry, 1999) | Journal of Arboriculture | San Luis Obispo County, California, USA | Case series | χ²-analysis | Decay  Structural defects | Urban | Root |
| 38. | (Eisner, Gilman, & Grabosky, 2002) | Journal of Arboriculture | Gainesville, Florida, USA | Experiment (in the field) | Biomechanical analysis  General Linear Models | Wind | Forest | Branch |
| 39. | (Elie & Ruel, 2005) | Canadian Journal of Forestry | Québec, Quebec, Canada | Experiment (in the field) | Biomechanical modelling (ForestGales)  Regression analysis | Wind  Anchorage strenght | Forest | Stem  Root |
| 40. | (Falinski, 1978) | Vegetatio | Bialowieza National Park, Poland | Case study | n.a. | Wind | Forest | Stem |
| 41. | (Faulkner & Malcolm, 1972) | Forestry | Pickering Vale, Scotland, UK | Experiment (in the field) | Regression analysis | Anchorage strength | Forest | Root |
| 42. | (Flesch & Wilson, 1999) | Agricultural and Forest Meteorology | Hotchkiss River, Manning Alberta Canada | Experiment (in the field) | Biomechanical analysis | Wind | Forest | Stem |
| 43. | (Foster, 1988) | Journal of Ecology | Harvard Forest, Petersham, Massachusetts, USA | Case study | Regression analysis | Hurricane | Forest | Stem |
| 44. | (Francis, 2000) | Journal of Arboriculture | San Juan, Puerto Rico, USA | Case study | Descriptive statistics  Regression analysis | Hurricane Hugo | Urban | Stem |
| 45. | (Fraser, 1962) | Forestry | Northumberland, UK | Experiment (in the field) | Biomechanical analysis  Regression analysis | Wind  Fungi | Forest | Stem |
| 46. | (Fredericksen, Hedden, & Williams, 1993) | Canadian Journal of Forest Research | Hobcaw Forest, Waccamaw Peninsula, Georgetown County, South Carolina, USA | Case study | Regression analysis | Wind | Forest | Stem |
| 47. | (Gardiner, Stacey, Belcher, & Wood, 1997) | Forestry | Kershope Forest, Cumbria, UK | Experiment (in the field) | Biomechanical analysis | Wind | Forest | Stem |
| 48. | (Gerhold & Johnson, 2003) | Journal of arboriculture | Pennsylvania, USA | Case study | Descriptive statistics  Regression analysis | Root spread and depth | Forest | Root |
| 49. | (Gifford, 1966) | The American Midland Naturalist | Farmington, Utah, USA | Case study | Descriptive statistics | Root spread and depth | Forest | Root |
| 50. | (Gilman, 1989) | Journal of Environmental Horticulture | East Brunswick, New York, USA | Case study | Regression analysis | Root spread and depth | Forest | Root |
| 51. | (Gilman, 2003) | Journal of Arboriculture | Agricultural Experiment Station, Florida, USA | Experiment (in the field) | Regression analysis | Branch trunk ratio | Urban | Branch |
| 52. | (Gilman, Masters, & Grabosky, 2008) | Arboriculture & Urban Forestry | University of Florida,  Alachua County, Florida, USA | Experiment (in the field) | Analysis of Variance | Pruning | Orchard | Branch |
| 53. | (Gilmore, 2001) | Forest ecology and management | University of Minnesota, Grand Rapids, Minnesota, USA | Case study | Regression analysis | Morphological characteristics | Solitary | Stem |
| 54. | (Glover, 1951) | East African Agricultural and Forestry Journal | Somalia | Case study | Descriptive statistics | Root spread and depth | Desert | Root |
| 55. | (Gurau, Cionca, Mansfield-Williams, Sawyer, & Zeleniuc, 2008) | Wood & Fiber Science | Forest Products  Research Center, High Wycombe, UK | Experiment (laboratory) | Biomechanical analysis | Wind | Forest | Branch  Stem |
| 56. | (Haidari, Jalilvand, Aghajani, & Nasiri, 2013) | International Journal of Advanced Biological and Biomedical Research | Babol, Mazandaran, Iran | Case-control | Analysis of variance  Descriptive statistics | Decay  Structural defects | Urban | Stem |
| 57. | (Hale, Gardiner, Wellpott, Nicoll, & Achim, 2012) | European Journal of Forest Resesarch | Clocaenog, Kyloe, Harwood, Kershope, Wales, UK | Experiment (in the field) | Biomechanical analysis  Correlation analysis | Wind | Forest | Stem |
| 58. | (Harcombe & Marks, 1983) | Oecologia | The Wier Forest, Lumberton, Hardin County, Texas, USA | Case study | Regression analysis | Wind | Forest | Stem |
| 59. | (Hassinen, Lemettinen, Peltola, Kellomäki, & Gardiner, 1998) | Agricultural and Forest Meteorology | Mekrijävi, Joensuu, Finnland | Experiment (in the field) | Descriptive statistics  Spectral analysis | Wind | Forest | Stem |
| 60. | (Hauer, Wang, & Dawson, 1993) | Journal of Arboriculture | Urbana, Illinois, USA | Case study | Descriptive statistics  Biomechanical analysis | Ice storm | Urban | Stem |
| 61. | (Hedden, Fredericksen, & Williams, 1995) | Canadian Journal of Forest Research | Hobcaw Forest, Waccamaw Penninsula, Georgetown County, South Carolina, USA | Experiment (in the field) | Regression analysis  Biomechanical analysis | Wind | Forest | Stem |
| 62. | (Heräjärvi, 2004) | Wood Science Technology | Finnland | Experiment (laboratory) | Biomechanical analysis | Wind | Forest | Stem |
| 63. | (Heyward, 1933) | Ecology | Choctawhatchee National Forest, Pensacola, Florida | Case study | Graphical analysis | Root spread and depth | Forest | Root |
| 64. | (Jahani, 2019) | International Journal of Environmental Science and Technology | Tehran, Iran | Case study | Artificial Neural Network  Sensitivity analysis | Urban conditions | Urban | Stem |
| 65. | (James, Hallam, & Spencer, 2013a) | Agricultural and Forest Meteorology | Australia | Experiment (in the field) | Biomechanical analysis | Wind | Forest | Root |
| 66. | (James, Hallam, & Spencer, 2013b) | Biosystems Engineering | Victoria, Australia | Experiment (in the field) | Biomechanical analysis | Wind | Urban | Root |
| 67. | (James, Haritos, & Ades, 2006) | American Journal of Botany | Australia | Experiment (in the field) | Biomechanical modelling and analysis | Wind | Urban | Stem |

| 68. | (Jim, 2005) | Journal of Environmental Management | Hong Kong | Case study | Descriptive statistics  Principal component analysis | Civil engineering Structural defects  Decay  Rooting space | Urban | Stem |
| --- | --- | --- | --- | --- | --- | --- | --- | --- |
| 69. | (Jim & Liu, 1997) | Landscape and Urban Planning | Guangzhou, China | Case study | Descriptive statistics | Storm | Urban | Stem |
| 70. | (Jonsson et al., 2007) | Trees | Davos, Switzerland | Experiment (in the field) | Biomechanical modelling | Wind | Forest | Stem |
| 71. | (Jonsson et al., 2006) | Plant and Soil | Davos, Switzerland | Case study | Biomechanical modelling | Morphological characteristics | Forest | Stem |
| 72. | (Kamimura, Gardiner, & Koga, 2017) | Forestry | Ashoro Research Forest, Kyushu University Hokkaido Island, Japan | Experiment (in the field) | Biomechanical analysis  Logistic regression analysis | Wind | Forest | Stem |
| 73. | (Kamimura, Kitagawa, Saito, & Mizunaga, 2012) | European Journal of Forest Research | Kamiatago forests, Shizuoka University, Shizuoka, Japan | Experiment (in the field) | Biomechanical analysis  Analysis of Covariance | Wind | Forest | Stem |
| 74. | (Kamo, Konoshima, & Yoshimoto, 2016) | Formath | Toyama Prefecture,  Japan | Experiment (in the field) | Cox regression modelling | Wind | Forest | Stem |
| 75. | (Kane, 2007) | Arboriculture & Urban Forestry | Virginia Tech campus, Blacksburg, Virginia, USA | Experiment (in the field) | Biomechanical analysis  Regression analysis | Attachment failure | Urban | Branch |
| 76. | (Kane, 2008) | Urban Forestry & Urban Greening | Nickerson state park, Barnstable County, Massachusetts, USA | Case study | Analysis of variance  Logistic regression | Storm | Forest | Stem |
| 77. | (Kane, 2014) | Trees | Pelham, Massachusetts, USA | Experiment (in the field) | Descriptive statistics  Regression analysis | Wind | Forest | Stem |
| 78. | (Kane & Clouston, 2008) | Arboriculture & Urban Forestry | Belchertown, Massachusetts, USA | Experiment (in the field) | Regression analysis  Biomechanical analysis | Wind | Urban | Stem |
| 79. | (Kane, Farrell, Zedaker, Lofersky, & Smith, 2008) | Arboriculture & Urban Forestry | Watkins Nursery  in Midlothian, Virginia, USA | Experiment (laboratory) | Regression analysis  Analysis of variance | Included bark | Urban | Branch |
| 80. | (Kane & Finn, 2014) | SpringerPlus | University of  Massachusetts in Amherst, USA | Case study | Generalized linear mixed effects model  Analysis of variance | Snowstorm | Urban | Branch |
| 81. | (Kane & James, 2011) | Canadian Journal of Forest Research | Charlotte, North Carolina, USA | Experiment (in the field) | Analysis of variance | Wind | Solitary | Stem |
| 82. | (Kane, Modarres-Sadeghi, James, & Reiland, 2014) | Trees | Belchertown, Massachusetts USA | Experiment (in the field) | Analysis of variance  Regression analysis | Wind | Urban | Stem |
| 83. | (Kane & Smiley, 2006) | Canadian Journal of Forest Research | Charlotte, North Carolina, USA | Experiment (in the field) | Analysis of variance  Biomechanical analysis | Wind | Orchard | Stem |
| 84. | (King, 1986) | Ecology | Wisconsin, USA | Experiment (in the field) | Regression analysis | Wind | Forest | Stem |
| 85. | (Kontogianni, Tsitsoni, & Goudelis, 2011) | Ecological Engineering | Thessaloniki, Greece | Experiment (in the field) | Regression analysis | Wind | Urban | Stem |
| 86. | (Leaf, Leonard, & Berglund, 1971) | Ecology | Charles Lathrop Pack Forest, Upper Hudson River Valey, Adirondack Mountains, New York state, USA | Case study | Descriptive statistics | Root spread and depth | Solitary | Root |
| 87. | (Lilly & Sydnor, 1995) | Journal of Arboriculture | Ohio State University, Ohio, USA | Experiment (in the field) | Biomechanical analysis  Least significant difference | Snow and ice loads | Urban | Branch |
| 88. | (Lopes, Oliveira, Fragoso, Andrade, & Pedro, 2009) | Internatonal Scientific Conference "Bioclimatology and Natural Hazards" | Lisbon, Portugal | Case study | Descriptive statistics | Storm | Urban | Stem |
| 89. | (Luley, Nowak, & Greenfield, 2009) | Arboriculture & Urban Forestry | Albany, Buffalo, Rochester, Syracuse, New York, USA | Case study | χ²-analysis | Decay | Urban | Stem |
| 90. | (Luley, Plenınger, & Sisinni, 2001) | Tree Structure and Mechanics Conference Proceedings: How Trees Stand Up and Fall Down. USA: International Society of Arboriculture | Rochester, New York, USA | Case study | Regression analysis | Wind Pruning | Urban | Branch |
| 91. | (Luley, Sisinni, & Pleninger, 2002) | Journal of Arboriculture | Rochester, New York, USA | Case control study | Least Square Difference | Pruning | Urban | Branch |
| 92. | (Lundström, Jonas, Stöckli, & Ammann, 2007) | Tree Physiology | Zürich, Switzerland | Experiment (in the field) | Biomechanical analysis  Regression analysis | Anchorage strength | Forest | Root |
| 93. | (Lundström, Jonsson, & Kalberer, 2007) | Plant Soil | Switzerland | Experiment (in the field) | Descriptive statistics | Wind | Forest | Stem |
| 94. | (Mattheck, Bethge, & Tesari, 2006) | Trees | Germany | Case study | Biomechanical analysis | Decay | Forest | Stem |
| 95. | (Mattheck, Bethge, & West, 1994) | Trees | Tasmania, Australia  South-West Australia | Case study | Biomechanical analysis | Decay | Forest | Stem |
| 96. | (McQuilkin, 1936) | Journal of agricultural research | Allegheny Forest Experiment Station, New Jersey, USA | Case study | Descriptive statistics | Root spread and depth | Forest | Root |
| 97. | (Miesbauer, Gilman, & Giurcanu, 2014) | Arboriculture & Urban Forestry | Florida, USA | Experiment (in the field) | Analysis of variance  Regression analysis | Wind | Urban | Stem |
| 98. | (Miesbauer, Gilman, Masters, & Nitesh, 2014) | Urban Forestry & Urban Greening | Environmental Horticulture Landscape Experimental laboratory, University of Florida, Gainesville, Florida, USA | Experiment (laboratory) | Biomechanical analysis  Analysis of variance | Ice, snow, wind | Urban | Branch |
| 99. | (Milne, 1986) | Proceedings Workshop Danish Forest Experiment Station and EC | Moffat forest, Scotland, UK | Experiment (in the field) | Descriptive statistics  Biomechanical analysis | Wind | Forest | Stem |
| 100. | (Milne, 1991) | Tree Physiology | Moffat forest, Scotland, UK | Experiment (in the field) | Biomechanical analysis | Wind | Forest | Branch |
| 101. | (Moore, 2000) | Forest Ecology and Management | New Zealand, 6 locations | Experiment (in the field) | Biomechanical analysis  Regression analysis | Wind | Forest | Stem |
| 102. | (Moore & Maguire, 2004) | Trees | Not available | Literature review | Regression analysis | Wind | Forest | Stem |
| 103. | (Moore & Maguire, 2005) | Trees | Corvallis, Oregon, USA | Experiment (in the field) | Regression analysis  Biomechanical analysis | Wind | Forest | Stem |
| 104. | (Moore & Maguire, 2008) | Tree Physiology | Corvallis, Oregon, USA | Case study | Descriptive statistics | Wind | Forest | Stem |
| 105. | (Mueller & Cline, 1959) | Soil Science | Ithaca, New York, USA | Case study | Regression analysis | Wind  Anchorage strength | Forest | Root |
| 106. | (Nicoll, Berthier, et al., 2016) | Trees | Leanachan Forest, Scotland, UK | Case study | Variance analysis | Root architecture | Forest | Root |
| 107. | (Nicoll, Gardiner, Rayner, & Peace, 2006) | Canadian Journal of Forest Research | UK | Experiment (in the field) | Regression analysis | Anchorage strength | Forest | Root |
| 108. | (Nicoll & Ray, 1996) | Tree Physiology | Crookburn, Kershope Forest, Cumbria, UK | Experiment (in the field) | Analysis of variance | Wind  Anchorage strength | Forest | Root |
| 109. | (Nilsen, Sharifi, Rundel, Jarrell, & Virginia, 1983) | Ecology | Sonoran Desert, California, USA | Case study | Descriptive statistics  Regression analysis | Drought | Desert | Root |
| 110. | (Nowak, Kuroda, & Crane, 2004) | Urban Forestry & Urban Greening | Baltimore, Maryland, USA | Cohort study | Descriptive statistics | Tree size, tree health, tree species, adjacent land use | Urban | Stem |
| 111. | (O'Brien, Hubbell, Spiro, Condit, & Foster, 1995) | Ecology | Barro Colorado Island, Panama | Case study | Biomechanical modelling | Morphological characteristics | Urban | Stem |
| 112. | (Oliver & Mayhead, 1974) | Forestry | Thetford forest park, Brandon, UK | Experiment (in the field) | Biomechanical analysis | Wind | Forest | Stem |
| 113. | (Onoda, Richards, & Westoby, 2010) | New Phytologist | Ku-ring-gai Chase National Park, Sydney, Australia | Experiment (laboratory) | Linear mixed models  Analysis of variance | Wind | Forest | Stem |
| 114. | (Ostertag, Silver, & Lugo, 2005) | Biotropica | Luquillo  Experimental Forest, Puerto Rico | Case study | Maximum likelihood ratio tests | Morphological characteristics | Forest | Branch |
| 115. | (Papesch, Moore, & Hawke, 1997) | New Zealand Journal of Forestry Science | Eyrewall Forest, Canterbury, UK | Experiment (in the field) | Analysis of covariance  Regression analysis | Wind | Forest | Stem |
| 116. | (Peltola & Kellomäki, 1993) | Silva fennica | Joensuu, Finland | Case study | Biomechanical modelling and analysis | Wind | Forest | Stem |
| 117. | (Peltola, Kellomäki, Hassinen, & Granander, 2000) | Forest Ecology and Management | Finland | Experiment (in the field) | Biomechanical analysis | Wind | Forest | Stem |
| 118. | (Peltola, Kellomäki, Väisänen, & Ikonen, 1999) | Canadian Journal of Forest Research | Joensuu, Finland | Case study | Biomechanical modelling and analysis | Wind | Forest | Stem |
| 119. | (Pereira & Hosegood, 1962) | Journal of Soil Science | Kinale, Kenya | Case study | Regression analysis | Root spread and depth | Forest | Root |
| 120. | (Peterson & Claassen, 2013) | Forestry | Stanislaus, Sutter and San Joaquin counties, California, USA | Experiment (in the field) | Analysis of covariance, Ordinary Least Squares | Wind | Urban | Stem |
| 121. | (Pfisterer, 2003) | Proceedings Second International Symposium on Plant Health in Urban Horticulture | Germany | Experiment (laboratory) | Biomechanical analysis | Wind | Orchard | Stem |
| 122. | (Pourhashemi, Esmaeilpour, & Heidari, 2012) | Iranian Journal of Forestry | Iran, Teheran | Experiment (laboratory) | Descriptive statistics | Drought | Urban | Stem |
| 123. | (Putz, Coley, Lu, Montalvo, & Aiello, 1983) | Canadian Journal of Forest Research | Barro Colorado Island, Republic of Panama | Case study | Discriminant analysis | Buttressing | Forest | Stem |
| 124. | (Putz & Sharitz, 1991) | Canadian Journal of Forest Research | Congaree Swamp, South Carolina, USA | Case study | Likelihood ratio tests  χ²-analysis | Hurricane | Forest | Stem |
| 125. | (Ray & Nicoll, 1998) | Forestry | Crookburn, Kershope Forest, Cumbria, UK | Experiment (in the field) | Regression analysis  Biomechanical analysis | Soil saturation  Root spread and depth | Forest | Root |
| 126. | (Reilly, 1991) | Biotropica | Virgin Islands National Park, St. John, U.S | Case study | Chi square tests | Morphological characteristics | Forest | Stem |
| 127. | (Ribeiro et al., 2016) | Forest Ecology and Management | 90 km north of Manaus, the capital of Amazonas State, Brazil | Experiment (in the field) | Analysis of Covariance | Wind | Forest | Stem |
| 128. | (Rigg & Harrar, 1931) | American Journal of Botany | Evans Creek, Seattle, Washington, USA | Case study | Descriptive statistics | Root spread and depth | Forest | Root |
| 129. | (Rodriguez, De Langre, & Moulia, 2008) | American Journal of Botany | New Zealand | Case study | Finite element modelling and analysis  Orthogonal regression analysis | Wind | Solitary | Stem |
| 130. | (Roodbaraky, Baker, Dawson, & Wright, 1994) | Journal of Wind Engineering and Industrial Aerodynamics | Blidworth, Nottinghamshire, UK | Case study | Biomechanical analysis | Wind | Urban | Stem |
| 131. | (Sánchez-Medina, Ayuga-Téllez, Contato-Carol, Grande-Ortiz, & González-García, 2017) | Arboriculture & Urban Forestry | Santiago del Estero, Argentina | Case study | Descriptive statistics  (Canonical) Correlation analysis | Morphological characteristics | Urban | Stem |
| 132. | (Saunderson, England, & Baker, 1999) | Journal of Theoretical Biology | Not available | Case study | Biomechanical modelling | Wind | Forest | Stem |
| 133. | (Sellier & Fourcaud, 2009) | American Journal of Botany | New Zealand | Case study | Finite element modelling and analysis | Wind | Solitary | Stem |
| 134. | (Sellier, Fourcaud, & Lac, 2006) | Tree Physiology | Canada | Case study | Finite element modelling and analysis | Wind | Forest | Stem |
| 135. | (Sharma, Bílek, Vacek, & Vacek, 2017) | Trees | Czech Republic | Case study | Biomechanical modelling  Regression analysis | Wind | Forest | Stem |
| 136. | (Silins, Lieffers, & Bach, 2000) | Trees | Whitecourt, Alberta, Canada | Experiment (in the field) | Biomechanical analysis | Wind | Forest | Stem |
| 137. | (Shin et al., 2016) | Proceedings at the 7th ICCM2016 | Kyungpook National University, Korea | Experiment (in the field) | Biomechanical analysis | Wind | Orchard | Stem |
| 138. | (Slater & Ennos, 2013) | Trees | Prestwich Country Park, Manchester, UK | Experiment (laboratory) | Biomechanical analysis  Analysis of variance | Ice, snow, wind | Orchard | Branch |
| 139. | (Slater & Ennos, 2015) | Arboriculture & Urban Forestry | Prestwich Country Park, Manchester, UK | Experiment (laboratory) | Biomechanical analysis  Analysis of variance | Included bark | Orchard | Branch |
| 140. | (Smiley, 2003) | Journal of Arboriculture | Bartlett Tree Research Laboratories, Charlotte, North Carolina, USA | Experiment (in the field) | Regression analysis | Included bark | Urban | Branch |
| 141. | (Smiley & Fraedrich, 1992) | Journal of Arboriculture | Charlotte, North Carolina, USA | Case study | Graphical analysis | Decay | Urban | Stem |
| 142. | (Smith, 1964) | The Forestry Chronicle | UBC Forest Haney, Paul Lake, Kamloops, Canada | Case study | Descriptive statistics  Regression analysis | Typhoon | Forest | Root |
| 143. | (Smith, Watts, & James, 1987) | Canadian Journal of Forest Research | Kapuskasing, Ontario, Canada | Experiment (in the field) | Biomechanical analysis  Regression analysis | Wind | Forest | Stem |
| 144. | (Soethe, Lehmann, & Engels, 2006) | Plant and Soil | Estación Cienífica San Francisco, Podocarpus National Park, Ecuador | Case study | Biomechanical analysis  Student’s t-test | Root spread and depth | Forest | Root |
| 145. | (Somerville, 1979) | New Zealand Journal of Forest Science | Eyrewell State Forest, New Zealand | Experiment (in the field) | Analysis of variance  Student’s t-test | Wind,  Root spread and depth | Forest | Root |
| 146. | (Spatz & Bruechert, 2000) | Forest Ecology and Management | Altglashütten, Black Forest, Germany | Case study | Biomechanical analysis | Wind | Forest | Stem |
| 147. | (Spatz, Brüchert, & Pfisterer, 2007) | American Journal of Botany | Freiburg, Germany | Experiment (in the field) | Biomechanical analysis  Regression analysis | Wind | Forest | Branch |
| 148. | (Spatz & Theckes, 2013) | American Journal of Botany | Freiburg, Germany | Experiment (laboratory) | Biomechanical analysis | Wind | Forest | Branch |
| 149. | (Štofko & Kodrík, 2008) | Journal of Forest Science | Hnilé Blatá Mountains, Czech Republic | Case control study | Correlation analysis | Wind | Forest | Root |
| 150. | (Stokes, 1999) | Plant and Soil | Forêt de l’Hermitage, Gironde, France | Experiment (in the field) | Regression analysis | Wind | Forest | Root |
| 151. | (Stokes et al., 2005) | Plant and Soil | Forêt Domaniale de Vaujany, Vall´ee de l’Eau d’Olle, Is`ere, France | Experiment (in the field) | Analysis of variance  Biomechanical analysis | Wind | Forest | Root |
| 152. | (Stone & Kalisz, 1991) | Forest Ecology and Management | Florida Agricultural Experiment Station, USA | Literature review | Descriptive statistics | Root spread and depth | Forest | Root |
| 153. | (Strong & Roi, 1983) | Canadian Journal of Forest Research | Alberta, Canada | Case study | Descriptive statistics | Root spread and depth | Forest | Root |
| 154. | (Sugden, 1962) | The Forestry Chronicle | Petawawa Forest Experiment Station, Ontario, Canada | Experiment (in the field) | Graphical analysis pruning | Wind | Forest | Stem |
| 155. | (Sundarapandian, Mageswaran, Gandhi, & Dar, 2014) | Current World Environment | Pondicherry University Campus, Puducherry, India | Case study | Analysis of variance | Cyclone | Urban | Stem |
| 156. | (Swiecki, Bernhardt, Drake, & Costello, 2006) | Phytosphere research | Marin County, California, USA | Case study | Logistic regression  χ²-analysis | Decay | Forest | Stem |
| 157. | (Terho, 2009) | Urban Forestry & Urban Greening | Helsinki, Finland | Case study | n.a. | Decay | Urban | Stem |
| 158. | (Turner, Slater, & Ennos, 2012) | Arboricultural Journal: The International Journal of Urban Forestry | Queen’s Square, Bristol city, UK | Experiment (laboratory) | Biomechanical analysis  Analysis of variance | Wind  Flexibility branch | Urban | Branch |
| 159. | (Vesselkin, Galako, Vlasenko, Shavnin, & Vorobeichik, 2015) | Contemporary problems of Ecology | Yekaterinburg, Russia | Case study | Analysis of (co)variance  Regression analysis | Morphological characteristics | Urban | Stem |
| 160. | (Vojáčková, Tippner, Horáček, Praus, Sebera, Brabec, 2019) | Arboriculture & Urban Forestry | Brno-Cerna Pole, Czech Republic | Experiment (in the field) | Finite element method  Sensitivity analysis | Wind, snow, ice | Urban | Branch |
| 161. | (Watson, Phillips & Marden, 1999) | Plant and Soil | Christchurch, New Zealand | Experiment (laboratory) | Biomechanical analysis | Root spread | Forest | Root |

References

1. Achim, A., Nicoll, B., Mochan, S., & Gardiner, B. (2003). Wind stability of trees on slopes. Paper presented at the Proceedings International Conference ‘Wind Effects on Trees’ September.

2. Achim, A., & Nicoll, B. C. (2009). Modelling the anchorage of shallow-rooted trees. Forestry, 82(3), 273-284.

3. Achim, A., Ruel, J.-C., & Gardiner, B. A. (2005). Evaluating the effect of precommercial thinning on the resistance of balsam fir to windthrow through experimentation, modelling, and development of simple indices. Canadian Journal of Forest Research, 35(8), 1844-1853.

4. Achim, A., Ruel, J.-C., Gardiner, B. A., Laflamme, G., & Meunier, S. (2005). Modelling the vulnerability of balsam fir forests to wind damage. Forest Ecology and Management, 204(1), 37-52.

5. Adams, M. E. (1967). A study of the ecology of Acacia mellifera, A. seyal and Balanites aegyptiaca in relation to land-clearing. Journal of Applied Ecology, 221-237.

6. Aldrich, W. W., Work, R. A., & Lewis, M. R. (1935). Pear root concentration in relation to soil moisture extraction in heavy clay soil. Jour. Agr. Res, 50, 975-988.

7. Ashton, D. H. (1975). The root and shoot development of Eucalyptus regnans F. Muell. Australian Journal of Botany, 23(6), 867-887.

8. Asner, G. P., & Goldstein, G. (1997). Correlating Stem Biomechanical Properties of Hawaiian Canopy Trees with Hurricane Wind Damage 1. Biotropica, 29(2), 145-150. doi: 10.1111/j.1744-7429.1997.tb00018.x

9. Baker, C. J. (1997). Measurements of the natural frequencies of trees. Journal of Experimental Botany, 48(5), 1125-1132.

10. Ballantyne, A. B. (1916). Bulletin No. 143-Fruit Tree Root Systems: Spread and Depth.

11. Bannan, M. W. (1940). The root systems of northern Ontario conifers growing in sand. American Journal of Botany, 27(2), 108-114.

12. Batanouny, K. H., & Abdel Wahab, A. M. (1973). Eco-physiological studies on desert plants. VIII. Root penetration of Leptadenia pyrotechnica (Forsk.) Decne. in Relation to Its Water Balance. Oecologia, 11(2), 151-161.

13. Bergeron, C., Ruel, J.-C., Elie, J.-G., & Mitchell, S. J. (2008). Root anchorage and stem strength of black spruce (Picea mariana) trees in regular and irregular stands. Forestry, 82(1), 29-41.

14. Berndt, H. W., & Gibbons, R. D. (1958). Root distribution of some native trees and understory plants growing on three sites within ponderosa pine watersheds in Colorado: Rocky Mountain Forest and Range Experiment Station.

15. Bishop, D. M. (1962). Lodgepole pine rooting habits in the Blue Mountains of north-eastern Oregon. Ecology, 43(1), 140-142.

16. Biswell, H. H. (1935). Effects of environment upon the root habits of certain deciduous forest trees. Botanical Gazette, 96(4), 676-708.

17. Brüchert, F., Becker, G., Speck, T. (2000). The mechanics of Norway spruce [Picea abies (L.) Karst]: mechanical properties of standing trees from different thinning regimes. Forest Ecology and Management, 135(1-3), 45-62.

18. Brüchert, F., & Gardiner, B. (2006). The effect of wind exposure on the tree aerial architecture and biomechanics of Sitka spruce (Picea sitchensis, Pinaceae). American Journal of Botany, 93(10), 1512-1521.

19. Buckley, G., Slater, D., & Ennos, R. (2015). Angle of inclination affects the morphology and strength of bifurcations in hazel (Corylus avellana L.). Journal of Arboriculture, 37(2), 99-112.

20. Cable, D. R. (1977). Seasonal use of soil water by mature velvet mesquite. Journal of Range Management, 4-11.

21. Cannon, J. B., Barrett, M. E., & Peterson, C. J. (2015). The effect of species, size, failure mode, and fire-scarring on tree stability. Forest Ecology and Management, 356, 196-203.

22. Castro-García, S., Blanco-Roldán, G. L., Gil-Ribes, J. A., & Agüera-Vega, J. (2008). Dynamic analysis of olive trees in intensive orchards under forced vibration. Trees, 22(6), 795-802.

23. Chiba, Y. (2000). Modelling stem breakage caused by typhoons in plantation Cryptomeria japonica forests. Forest Ecology and Management, 135(1-3), 123-131.

24. Ciftci, C., Arwade, S. R., Kane, B., & Brena, S. F. (2014). Analysis of the probability of failure for open-grown trees during wind storms. Probabilistic Engineering Mechanics, 37, 41-50. doi: 10.1016/j.probengmech.2014.04.002

25. Coile, T. S. (1937). Distribution of forest tree roots in North Carolina Piedmont soils. Journal of Forestry, 35(3), 247-257.

26. Coutts, M. P. (1983). Root architecture and tree stability. In Tree root systems and their mycorrhizas (pp. 171-188). Springer, Dordrecht.

27. Coutts, M. P. (1986). Components of tree stability in Sitka spruce on peaty gley soil. Forestry: An International Journal of Forest Research, 59(2), 173-197.

28. Crook, M. J., & Ennos, A. R. (1996). The anchorage mechanics of deep rooted larch, Larix europea× L. japonica. Journal of Experimental Botany, 47(10), 1509-1517.

29. Cucchi, V., Meredieu, C., Stokes, A., Berthier, S., Bert, D., Najar, M., Denis, A. & Lastennet, R. (2004). Root anchorage of inner and edge trees in stands of Maritime pine (Pinus pinaster Ait.) growing in different podzolic soil conditions. Trees, 18(4), 460-466.

30. Dahle, G. A., & Grabosky, J. C. (2010). Variation in modulus of elasticity (E) along Acer platanoides L.(Aceraceae) branches. Urban Forestry & Urban Greening, 9(3), 227-233.

31. Dahle, G. A., Holt, H. H., Chaney, W. R., Whalen, T. M., Cassens, D. L., Gazo, R., & McKenzie, R. L. (2006). Branch strength loss implications for silver maple (Acer saccharinum) converted from round-over to V-trim. Journal of Arboriculture, 32(4), 148.

32. Danjon, F., Fourcaud, T., & Bert, D. (2005). Root architecture and wind-firmness of mature Pinus pinaster. New Phytologist, 168(2), 387-400.

33. Davis, E. (1977). Root system of shrub live oak in relation to water yield by chaparral. Paper presented at the Hydrology and water resources in Arizona and the Southwest.

34. Day, M. W. (1944). The root system of aspen. American Midland Naturalist, 32(2), 502.

35. Dorren, L. K., & Berger, F. (2006). Stem breakage of trees and energy dissipation during rockfall impacts. Tree Physiology, 26(1), 63-71.

36. Eames, A. J., & Cox, L. G. (1945). A remarkable tree-fall and an unusual type of graft-union failure. American Journal of Botany, 32(6), 331-335.

37. Edberg, R., & Berry, A. (1999). Patterns of structural failures in urban trees: Coast live oak (Quercus agrifolia). Journal of Arboriculture, 25, 48-55.

38. Eisner, N. J., Gilman, E. F., & Grabosky, J. C. (2002). Branch morphology impacts compartmentalization of pruning wounds. Journal of Arboriculture, 28(2), 99-105.

39. Elie, J.-G., & Ruel, J.-C. (2005). Windthrow hazard modelling in boreal forests of black spruce and jack pine. Canadian Journal of Forest Research, 35(11), 2655-2663.

40. Falinski, J. B. (1978). Uprooted trees, their distribution and influence in the primeval forest biotope. Vegetatio, 38(3), 175-183.

41. Faulkner, M. E., & Malcolm, D. C. (1972). Soil physical factors affecting root morphology and stability of Scots pine on upland heaths. Forestry: An International Journal of Forest Research, 45(1), 23-36.

42. Flesch, T. K., & Wilson, J. D. (1999). Wind and remnant tree sway in forest cutblocks. II. Relating measured tree sway to wind statistics. Agricultural and forest meteorology, 93(4), 243-258.

43. Foster, D. R. (1988). Species and stand response to catastrophic wind in central New England, USA. The Journal of Ecology, 135-151.

44. Francis, J. K. (2000). Comparison of hurricane damage to several species of urban trees in San Juan, Puerto Rico. Journal of Arboriculture, 26(4), 189-197.

45. Fraser, A. (1962). The soil and roots as factors in tree stability. Forestry: An International Journal of Forest Research, 34(2), 117-127.

46. Fredericksen, T. S., Hedden, R. L., & Williams, S. A. (1993). Testing loblolly pine wind firmness with simulated wind stress. Canadian Journal of Forest Research, 23(9), 1760-1765.

47. Gardiner, B. A., Stacey, G. R., Belcher, R. E., & Wood, C. J. (1997). Field and wind tunnel assessments of the implications of respacing and thinning for tree stability. Forestry: An International Journal of Forest Research, 70(3), 233-252.

48. Gerhold, H. D., & Johnson, A. D. (2003). Root dimensions of landscape tree cultivars. Arboriculture & Urban Forestry, 29(6), 322.

49. Gifford, G. F. (1966). Aspen root studies on three sites in northern Utah. American Midland Naturalist, 132-141.

50. Gilman, E. F. (1989). Predicting root spread from trunk diameter and branch spread. Arboricultural Journal, 13(1), 25-32.

51. Gilman, E. F. (2003). Branch-to-stem diameter ratio affects strength of attachment. Journal of Arboriculture, 29(5), 291-294.

52. Gilman, E. F., Masters, F., & Grabosky, J. C. (2008). Pruning affects tree movement in hurricane force wind. Journal of Arboriculture, 34(1), 20.

53. Gilmore, D. W. (2001). Equations to describe crown allometry of Larix require local validation. Forest Ecology and Management, 148(1-3), 109-116.

54. Glover, P. E. (1951). The Root Systems of Some British Somaliland Plants—IV. The East African Agricultural Journal, 17(1), 38-50. doi: 10.1080/03670074.1951.11664783

55. Gurau, L., Cionca, M., Mansfield-Williams, H., Sawyer, G., & Zeleniuc, O. (2008). Comparison of the mechanical properties of branch and stem wood for three species. Wood and Fiber Science, 40(4), 647-656.

56. Haidari, M., Jalilvand, H., Aghajani, H., & Nasiri, M. (2013). Evaluation of Pinus eldarica hazardous criterias in the Babol city (Mazandaran province, northern state of Iran). International journal of Advanced Biological and Biomedical Research, 1(9), 957-962.

57. Hale, S. E., Gardiner, B. A., Wellpott, A., Nicoll, B. C., & Achim, A. (2012). Wind loading of trees: influence of tree size and competition. European Journal of Forest Research, 131(1), 203-217.

58. Harcombe, P. A., & Marks, P. L. (1983). Five years of tree death in a Fagus-Magnolia forest, southeast Texas (USA). Oecologia, 57(1-2), 49-54.

59. Hassinen, A., Lemettinen, M., Peltola, H., Kellomäki, S., & Gardiner, B. (1998). A prism-based system for monitoring the swaying of trees under wind loading. Agricultural and forest meteorology, 90(3), 187-194.

60. Hauer, R. J., Wang, W., & Dawson, J. O. (1993). Ice storm damage to urban trees. Journal of Arboriculture, 19, 187-187.

61. Hedden, R., Fredericksen, T., & Williams, S. (1995). Modeling the effect of crown shedding and streamlining on the survival of loblolly pine exposed to acute wind. Canadian Journal of Forest Research, 25(5), 704-712.

62. Heräjärvi, H. (2004). Static bending properties of Finnish birch wood. Wood Science and Technology, 37(6), 523-530.

63. Heyward, F. (1933). The root system of longleaf pine on the deep sands of western Florida. Ecology, 14(2), 136-148.

64. Jahani, A. (2019). Sycamore failure hazard classification model (SFHCM): an environmental decision support system (EDSS) in urban green spaces. International journal of environmental science and technology, 16(2), 955-964.

65. James, K., Hallam, C., & Spencer, C. (2013a). Measuring tilt of tree structural root zones under static and wind loading. Agricultural and forest meteorology, 168, 160-167.

66. James, K., Hallam, C., & Spencer, C. (2013b). Tree stability in winds: Measurements of root plate tilt. Biosystems Engineering, 115(3), 324-331. doi: 10.1016/j.biosystemseng.2013.02.010

67. James, K. R., Haritos, N., & Ades, P. K. (2006). Mechanical stability of trees under dynamic loads. American Journal of Botany, 93(10), 1522-1530.

68. Jim, C. Y. (2005). Monitoring the performance and decline of heritage trees in urban Hong Kong. Journal of Environmental Management, 74(2), 161-172.

69. Jim, C. Y., & Liu, H. H. T. (1997). Storm damage on urban trees in Guangzhou, China. Landscape and Urban Planning, 38, 45-59.

70. Jonsson, M. J., Foetzki, A., Kalberer, M., Lundström, T., Ammann, W., & Stöckli, V. (2006). Root-soil rotation stiffness of Norway spruce (Picea abies (L.) Karst) growing on subalpine forested slopes. Plant and Soil, 285(1-2), 267-277.

71. Jonsson, M. J., Foetzki, A., Kalberer, M., Lundström, T., Ammann, W., & Stöckli, V. (2007). Natural frequencies and damping ratios of Norway spruce (Picea abies (L.) Karst) growing on subalpine forested slopes. Trees, 21(5), 541-548.

72. Kamimura, K., Gardiner, B., & Koga, S. (2017). Observations and predictions of wind damage to Larix kaempferi trees following thinning at an early growth stage. Forestry: An International Journal of Forest Research, 90(4), 530-540.

73. Kamimura, K., Kitagawa, K., Saito, S., & Mizunaga, H. (2012). Root anchorage of hinoki (Chamaecyparis obtuse (Sieb. Et Zucc.) Endl.) under the combined loading of wind and rapidly supplied water on soil: analyses based on tree-pulling experiments. European Journal of Forest Research, 131(1), 219-227.

74. Kamo, K., Konoshima, M., & Yoshimoto, A. (2016). Statistical analysis of tree-forest damage by snow and wind: Logistic regression model for tree damage and cox regression for tree survival. FORMATH, Forest Recources & Mathematical modelling, 15, 44-55. doi: 10.15684/formath.15.005

75. Kane, B. (2007). Branch Strength of Bradford Pear (Pyrus calleryana var.Bradford'). Journal of Arboriculture, 33(4), 283.

76. Kane, B. (2008). Tree failure following a windstorm in Brewster, Massachusetts, USA Urban Forestry & Urban Greening 7(1), 15-23. doi: 10.1016/j.ufug.2007.11.001

77. Kane, B. (2014). Determining parameters related to the likelihood of failure of red oak (Quercus rubra L.) from winching tests. Trees, 28(6), 1667-1677.

78. Kane, B., & Clouston, P. (2008). Tree pulling tests of large shade trees in the genus Acer. Journal of Arboriculture, 34(2), 101.

79. Kane, B., Farrell, R. W., Zedaker, S. M., Lofersky, J. R., & Smith, D. W. (2008). Failure mode and prediction of the strength of branch attachments. Journal of Arboriculture, 34(5), 308-316.

80. Kane, B., & Finn, J. T. (2014). Factors affecting branch failures in open-grown trees during a snowstorm in Massachusetts, USA. SpringerPlus, 3(1), 720.

81. Kane, B., & James, K. R. (2011). Dynamic properties of open-grown deciduous trees. Canadian Journal of Forest Research, 41(2), 321-330.

82. Kane, B., Modarres-Sadeghi, Y., James, K. R., & Reiland, M. (2014). Effects of crown structure on the sway characteristics of large decurrent trees. Trees, 28(1), 151-159.

83. Kane, B., & Smiley, E. T. (2006). Drag coefficients and crown area estimation of red maple. Canadian Journal of Forest Research, 36(8), 1951-1958.

84. King, D. A. (1986). Tree form, height growth, and susceptibility to wind damage in Acer saccharum. Ecology, 67(4), 980-990.

85. Kontogianni, A., Tsitsoni, T., & Goudelis, G. (2011). An index based on silvicultural knowledge for tree stability assessment and improved ecological function in urban ecosystems. Ecological Engineering, 37(6), 914-919.

86. Leaf, A. L., Leonard, R. E., & Berglund, J. V. (1971). Root Distribution of a Plantation‐Grown Red Pine in an Outwash Soil. Ecology, 52(1), 153-158.

87. Lilly, S., & Sydnor, T. D. (1995). Comparison of branch failure during static loading of silver and Norway maples. Journal of Arboriculture, 21, 302-305.

88. Lopes, A., Oliveira, S., Fragoso, M., Andrade, J. A., & Pedro, P. (2009). Wind risk assessment in urban environments: the case of falling trees during windstorm events in Lisbon Bioclimatology and natural hazards (pp. 55-74): Springer.

89. Luley, C. J., Nowak, D. J., & Greenfield, E. J. (2009). Frequency and severity of trunk decay in street tree maples in four New York cities. Journal of Arboriculture, 35(2), 94.

90. Luley, C. J., Plenınger, A., & Sisinni, S. (2001). The effect of wind gusts on branch failures in the city of Rochester, New York, US. Paper presented at the Tree Structure and Mechanics Conference Proceedings: How Trees Stand Up and Fall Down: October.

91. Luley, C. J., Sisinni, S., & Pleninger, A. (2002). The effect of pruning on service requests, branch failures, and priority maintenance in the city of Rochester, New York, US. Journal of Arboriculture, 28(3), 137-143.

92. Lundström, T., Jonas, T., Stöckli, V., & Ammann, W. (2007). Anchorage of mature conifers: resistive turning moment, root–soil plate geometry and root growth orientation. Tree Physiology, 27(9), 1217-1227.

93. Lundström, T., Jonsson, M. J., & Kalberer, M. (2007). The root–soil system of Norway spruce subjected to turning moment: resistance as a function of rotation. Plant and Soil, 300(1-2), 35-49.

94. Mattheck, C., Bethge, K., & Tesari, I. (2006). Shear effects on failure of hollow trees. Trees, 20(3), 329-333.

95. Mattheck, C., Bethge, K., & West, P. W. (1994). Breakage of hollow tree stems. Trees, 9(1), 47-50.

96. McQuilkin, W. E. (1936). Root Development of Pitch Pine, with Some Comparative Observations on Short Leaf Pine. Graduate School of Arts and Sciences, University of Pennsylvania.

97. Miesbauer, J. W., Gilman, E. F., & Giurcanu, M. (2014). Effects of tree crown structure on dynamic properties of Acer rubrum L. ‘Florida Flame’. Arboriculture & Urban Forestry, 40(4).

98. Miesbauer, J. W., Gilman, E. F., Masters, F. J., & Nitesh, S. (2014). Impact of branch reorientation on breaking stress in Liriodendron tulipifera L. Urban Forestry & Urban Greening, 13(3), 526-533.

99. Milne, R. (1986). Methods of modelling tree stem bending under wind loading, In Proceedings Minimising Wind Damage in Coniferous Stands. Proceedings of the workshop organized jointly by the Danish forest experiment station and the commission of the european communities, Løvenholm Castle, Denmark, March 3-7 (pp. 12-16).

100. Milne, R. (1991). Dynamics of swaying of Picea sitchensis. Tree Physiology, 9(3), 383-399.

101. Moore, J. R. (2000). Differences in maximum resistive bending moments of Pinus radiata trees grown on a range of soil types. Forest Ecology and Management, 135(1-3), 63-71.

102. Moore, J. R., & Maguire, D. A. (2004). Natural sway frequencies and damping ratios of trees: concepts, review and synthesis of previous studies. Trees, 18(2), 195-203.

103. Moore, J. R., & Maguire, D. A. (2005). Natural sway frequencies and damping ratios of trees: influence of crown structure. Trees, 19(4), 363-373.

104. Moore, J. R., & Maguire, D. A. (2008). Simulating the dynamic behavior of Douglas-fir trees under applied loads by the finite element method. Tree Physiology, 28(1), 75-83.

105. Mueller, O. P., & Cline, M. G. (1959). Effects of mechanical soil barriers and soil wetness on rooting of trees and soil-mixing by blow-down in central New York. Soil Science, 88(2), 107-111.

106. Nicoll, B.C., Berthier, S., Achim, A., Gouskou, K., Danjon, F., Van Beek., L.P.H. (2016). The architecture of Picea sitchensis structural root systems on horizontal and sloping terrain. Trees, 20(6), 701-712

107. Nicoll, B. C., Gardiner, B. A., Rayner, B., & Peace, A. J. (2006). Anchorage of coniferous trees in relation to species, soil type, and rooting depth. Canadian Journal of Forest Research, 36(7), 1871-1883.

108. Nicoll, B. C., & Ray, D. (1996). Adaptive growth of tree root systems in response to wind action and site conditions. Tree Physiology, 16(11-12), 891-898.

109. Nilsen, E. T., Sharifi, M. R., Rundel, P. W., Jarrell, W. M., & Virginia, R. A. (1983). Diurnal and seasonal water relations of the desert phreatophyte Prosopis glandulosa (honey mesquite) in the Sonoran Desert of California. Ecology, 64(6), 1381-1393.

110. Nowak, D. J., Kuroda, M., & Crane, D. E. (2004). Tree mortality rates and tree population projections in Baltimore, Maryland, USA. Urban Forestry & Urban Greening, 2(3), 139-147.

111. O'Brien, S. T., Hubbell, S. P., Spiro, P., Condit, R., & Foster, R. B. (1995). Diameter, height, crown, and age relationship in eight neotropical tree species. Ecology, 76(6), 1926-1939.

112. Oliver, H. R., & Mayhead, G. J. (1974). Wind measurements in a pine forest during a destructive gale. Forestry: An International Journal of Forest Research, 47(2), 185-194.

113. Onoda, Y., Richards, A. E., & Westoby, M. (2010). The relationship between stem biomechanics and wood density is modified by rainfall in 32 Australian woody plant species. New Phytologist, 185(2), 493-501.

114. Ostertag, R., Silver, W. L., & Lugo, A. E. (2005). Factors Affecting Mortality and Resistance to Damage Following Hurricanes in a Rehabilitated Subtropical Moist Forest 1. Biotropica, 37(1), 16-24. doi: 10.1111/j.1744-7429.2005.04052.x

115. Papesch, A., Moore, J., & Hawke, A. (1997). Mechanical stability of Pinus radiata trees at Eyrewell Forest investigated using static tests. New Zealand Journal of Forestry Science, 27(2), 188-204.

116. Peltola, H., & Kellomäki, S. (1993). A mechanistic model for calculating windthrow and stem breakage of Scots pines at stand age.

117. Peltola, H., Kellomäki, S., Hassinen, A., & Granander, M. (2000). Mechanical stability of Scots pine, Norway spruce and birch: an analysis of tree-pulling experiments in Finland. Forest Ecology and Management, 135(1-3), 143-153.

118. Peltola, H., Kellomäki, S., Väisänen, H., & Ikonen, V.-P. (1999). A mechanistic model for assessing the risk of wind and snow damage to single trees and stands of Scots pine, Norway spruce, and birch. Canadian Journal of Forest Research, 29(6), 647-661.

119. Pereira, H., & Hosegood, P. (1962). Comparative water-use of softwood plantations and bamboo forest. Journal of Soil Science, 13(2), 299-313.

120. Peterson, C. J., & Claassen, V. (2013). An evaluation of the stability of Quercus lobata and Populus fremontii on river levees assessed using static winching tests. Forestry, 86(2), 201-209.

121. Pfisterer J.A., editor Towards a better understanding of tree failure: Investigations into bending stresses of branch junctions and reiterates of European Filbert (Corylus avellana L.) as a model organism. Second International Symposium on plant health in urban horticulture; 2003 27-29 August; Berlin, Germany: Biologische Bundesanstalt für Land-und Forstwirtschaft.

122. Pourhashemi, M., Esmaeilpour, K., & Heidari, M. (2012). The assessment of hazardous oriental plane (Platanus orientalis Linn.) trees in Valiasr street of Tehran.

123. Putz, F. E., Coley, P. D., Lu, K., Montalvo, A., & Aiello, A. (1983). Uprooting and snapping of trees: structural determinants and ecological consequences. Canadian Journal of Forest Research, 13(5), 1011-1020.

124. Putz, F. E., & Sharitz, R. R. (1991). Hurricane damage to old-growth forest in Congaree Swamp National Monument, South Carolina, USA. Canadian Journal of Forest Research, 21(12), 1765-1770.

125. Ray, D., & Nicoll, B. C. (1998). The effect of soil water-table depth on root-plate development and stability of Sitka spruce. Forestry: An International Journal of Forest Research, 71(2), 169-182.

126. Reilly, A. E. (1991). The effects of Hurricane Hugo in three tropical forests in the US Virgin Islands. Biotropica, 414-419.

127. Ribeiro, G. H. P. M., Chambers, J. Q., Peterson, C. J., Trumbore, S. E., Marra, D. M., Wirth, C., Cannon, J. B., Négron-Juarez, R. I., Lima, A. J. N., de Paula, E. V. C. M. (2016). Mechanical vulnerability and resistance to snapping and uprooting for Central Amazon tree species. Forest Ecology and Management, 380, 1-10.

128. Rigg, G. B., & Harrar, E. S. (1931). The root systems of trees growing in sphagnum. American Journal of Botany, 18(6), 391-397.

129. Rodriguez, M., De Langre, E., & Moulia, B. (2008). A scaling law for the effects of architecture and allometry on tree vibration modes suggests a biological tuning to modal compartmentalization. American Journal of Botany, 95(12), 1523-1537.

130. Roodbaraky, H., Baker, C., Dawson, A., & Wright, C. (1994). Experimental observations of the aerodynamic characteristics of urban trees. Journal of Wind Engineering and Industrial Aerodynamics, 52, 171-184.

131. Sánchez-Medina, A., Ayuga-Téllez, E., Contato-Carol, L., Grande-Ortiz, M., & González-García, C. (2017). Selection of Tree-Size Variables for Appraisal Methods for Urban Trees According to Their Collinearity. Arboriculture & Urban Forestry, 43(3).

132. Saunderson, S., England, A., & Baker, C. (1999). A dynamic model of the behaviour of Sitka spruce in high winds. Journal of theoretical Biology, 200(3), 249-259.

133. Sellier, D., & Fourcaud, T. (2009). Crown structure and wood properties: influence on tree sway and response to high winds. American Journal of Botany, 96(5), 885-896.

134. Sellier, D., Fourcaud, T., & Lac, P. (2006). A finite element model for investigating effects of aerial architecture on tree oscillations. Tree Physiology, 26(6), 799-806.

135. Sharma, R. P., Bílek, L., Vacek, Z., & Vacek, S. (2017). Modelling crown width–diameter relationship for Scots pine in the central Europe. Trees, 31(6), 1875-1889.

136. Shin, S., Kang, I., Park, S., Lee, Y., Shin, K., Kim, W., & Kim, H. (2016). Gust effect factors and natural sway frequencies of trees. Paper presented at the The 7th International Conference on Computational Methods (ICCM2016), Berkeley, CA, USA.

137. Silins, U., Lieffers, V. J., & Bach, L. (2000). The effect of temperature on mechanical properties of standing lodgepole pine trees. Trees, 14(8), 424-428.

138. Slater, D., & Ennos, A. R. (2013). Determining the mechanical properties of hazel forks by testing their component parts. Trees, 27(6), 1515-1524.

139. Slater, D., & Ennos, A. R. (2015). The Level of Occlusion of Included Bark Affects the Strength of Bifurcations in Hazel. Arboriculture & Urban Forestry, 41(4), 194-207.

140. Smiley, E. T. (2003). Does Included Bark Reduce the Strength of Codominant Stems? Journal of Arboriculture, 29(2), 104-106.

141. Smiley, E. T., & Fraedrich, B. R. (1992). Determining strength loss from decay. Journal of Arboriculture, 18(4), 201-204.

142. Smith, J. H. G. (1964). Root spread can be estimated from crown width of Douglas fir, lodgepole pine, and other British Columbia tree species. The Forestry Chronicle, 40(4), 456-473.

143. Smith, V. G., Watts, M., & James, D. F. (1987). Mechanical stability of black spruce in the clay belt region of northern Ontario. Canadian Journal of Forest Research, 17(9), 1080-1091.

144. Soethe, N., Lehmann, J., & Engels, C. (2006). Root morphology and anchorage of six native tree species from a tropical montane forest and an elfin forest in Ecuador. Plant and Soil, 279(1-2), 173-185.

145. Somerville, A. (1979). Root anchorage and root morphology of Pinus radiata on a range of ripping treatments. NZJ For. Sci, 9(3), 294-315.

146. Spatz, H. C., Brüchert, F., & Pfisterer, J. (2007). Multiple resonance damping or how do trees escape dangerously large oscillations? American Journal of Botany, 94(10), 1603-1611.

147. Spatz, H. C., & Bruechert, F. (2000). Basic biomechanics of self-supporting plants: wind loads and gravitational loads on a Norway spruce tree. Forest Ecology and Management, 135(1-3), 33-44.

148. Spatz, H. C., & Theckes, B. (2013). Oscillation damping in trees. Plant science, 207, 66-71.

149. Štofko, P., & Kodrík, M. (2008). Comparison of the root system architecture between windthrown and undamaged spruces growing in poorly drained sites. J. For. Sci, 54, 150-160.

150. Stokes, A. (1999). Strain distribution during anchorage failure of Pinus pinaster Ait. at different ages and tree growth response to wind-induced root movement. Plant and Soil, 217, 17-27.

151. Stokes, A., Salin, F., Kokutse, A. D., Berthier, S., Jeannin, H., Mochan, S., Dorren, L., Kokutse, N., Ghani, M.A. & Fourcaud, T. (2005). Mechanical resistance of different tree species to rockfall in the French Alps. Plant and Soil, 278(1-2), 107-117.

152. Stone, E. L., & Kalisz, P. J. (1991). On the maximum extent of tree roots. Forest Ecology and Management, 46(1-2), 59-102.

153. Strong, W., & Roi, G. L. (1983). Root-system morphology of common boreal forest trees in Alberta, Canada. Canadian Journal of Forest Research, 13(6), 1164-1173.

154. Sugden, M. J. (1962). Tree sway period—a possible new parameter for crown classification and stand competition. The Forestry Chronicle, 38(3), 336-344.

155. Sundarapandian, S., Mageswaran, K., Gandhi, D. S., & Dar, J. A. (2014). Impact of thane cyclone on tree damage in Pondicherry University campus, Puducherry, India. Current World Environment, 9(2), 287.

156. Swiecki, T. J., Bernhardt, E., Drake, C., & Costello, L. R. (2006). Relationships between Phytophthora ramorum canker (sudden oak death) and failure potential in coast live oak. Paper presented at the In: Frankel, Susan J.; Shea, Patrick J.; and Haverty, Michael I., tech. coords. Proceedings of the sudden oak death second science symposium: the state of our knowledge. Gen. Tech. Rep. PSW-GTR-196. Albany, CA: Pacific Southwest Research Station, Forest Service, US Department of Agriculture: 427-453.

157. Terho, M. (2009). An assessment of decay among urban Tilia, Betula, and Acer trees felled as hazardous. Urban Forestry & Urban Greening, 8(2), 77-85.

158. Turner, S., Slater, D., & Ennos, A. R. (2012). Failure of forks in clonal varieties of Platanus x acerifolia. Journal of Arboriculture, 34(4), 179-189.

159. Veselkin, D., Galako, V., Vlasenko, V., Shavnin, S., & Vorobeichik, E. (2015). Relationship between the characteristics of the state of Scots pine trees and tree stands in a large industrial city. Contemporary Problems of Ecology, 8(2), 243-249.

160. Vojáčková, B., Tippner, J., Horáček, P., Praus, L., Sebera, V., & Brabec, M. (2019). Numerical Analysis of Branch Mechanical Response to Loading. Arboriculture & Urban Forestry, 45(4).

161. Watson, A., Phillips, C. & Marden, M. Root strength, growth, and rates of decay: root reinforcement changes of two tree species and their contribution to slope stability. Plant and Soil 217, 39–47 (1999). https://doi.org/10.1023/A:1004682509514
