# Supplementary material for: Understanding tree failure—A systematic review and meta-analysis
Source: PLoS One. 2021 Feb 16;16(2):e0246805. doi: 10.1371/journal.pone.0246805 (PMC7886209; doi:10.1371/journal.pone.0246805)
Supplement: S4 Text — (DOCX) [file pone.0246805.s008.docx]

**Data preparation for regressions**

Regression analysis was used to analyze the impact of several factors on tree failure. To conduct regressions, data extraction was necessary for the factors DBH, Height and Slenderness. When the raw data was presented in a study descriptive statistics like the means of each factor needed to be derived. How these are calculated is described in this protocol. The input factors for the data extraction are dichotomous, categorical or continuous.

Dichotomous data are:

- Event for failed and non-failed trees

Categorical data are:

- Climate zone the study location is situated in

Continuous data are:

- Mean, standard deviation and sample size of the factors DBH, Height and Slenderness
- Correlation coefficients, R² and p-value

When several units of measurement were presented for one particular factor, all data was extracted but the one most often found in studies was used for calculations. If a study reported a factor related to failure, the factor was included. Studies on tree failure investigating factors related to sway where the trees did not actually fail or studies which did not report one or more of the factors DBH, Height or Slenderness were excluded from the regression analysis. Slenderness was calculated in case of absence by dividing Height (m) over DBH (m).

The factors that were expressed in measures of the British Imperial System or in the US Customary System were converted to the International Systems of Units (SI). Non-SI units that are exactly defined values in terms of SI, were expressed in terms of SI. If mean and standard deviation were not present in the article, the equations used to calculate as mentioned by Field [1] are:

Mean: $\bar{x}=\frac{\sum x_{i}}{n}$ in case of one group of one population.

$\bar{x}=\frac{\sum n_{i}\cdot x_{i}}{\sum n_{i}}$ where $n_{i}$ are tree numbers failed from >1 sub-groups; $x_{i}$ are means of >1 groups.

Standard deviation: $s=\sqrt{\frac{\sum\left( x_{i}-\bar{x} \right)^{2}}{n-1}}$ for deviations of 1 group

$s=\sqrt{\left[ \frac{\sum(n_{i}-1)\cdot s_{i}^{2}}{\sum n_{i}-k}+\frac{\sum{n_{i}\left( x_{i}-\bar{x} \right)}^{2}}{\sum n_{i}-k} \right]}$ where $s_{i}$ are standard deviations from >1 group.

When the included study only reported the mean (or median), minimum and maximum and sample size, the mean and standard deviation were estimated as mentioned by Hozo, Djulbgovic and Hozo [2]:

Mean: $\bar{x}=\frac{\min+ 2\cdot median+max}{4}$ if n ≤ 25

$\bar{x}=median$ if n > 25

Standard deviation: $s=\sqrt{\frac{1}{12}\left[ \frac{\left( min-2median+max \right)^{2}}{4}+\left( max-min \right)^{2} \right]}$ if n ≤ 15

$s= \frac{min-max}{4}$ if 15 < n ≤ 70 and normally distributed

$s= \frac{min-max}{6}$ if n > 70, for any random distribution

**Results binary logistic regressions**

Results of multiple binary logistic regression analyses on the presence of failure in articles of the systematic review and the reported factors in these articles Diameter Breast Height (DBH), Height (m), Slenderness (m), Climate zones. The logistic regressions are executed in SPSS 26 [3] and Stata 16 [4] and provided exactly similar results.

First the results are presented of four different binary logistic regressions of studies on the factors DBH, Height, Slenderness and Climate zones (table 1). These results indicate whether each factor is an improvement towards a model in which no predictors for failure are included (null model). The model fit is significant for DBH and Height, but non-significant for Climate zones and Slenderness. The significance from the Hosmer and Lemeshow test is non-significant for Height and Slenderness, indicating a good model fit. The results of the χ²-test and the Hosmer and Lemeshow test conflict for DBH and Slenderness.

Table 1: Results of 4 binary logistic regressions of studies reporting failure on different factors and climate zones

| Ref. | Failure x factor | -2 log likelihood | Nagelkerke R² | Model fit χ² | df | Sig. | Sign. Hosmer & Lemeshow Test |
| --- | --- | --- | --- | --- | --- | --- | --- |
| A. | Climate zones | 303.335 | 0.016 | 2.649 | 4 | 0.104 | 0.017 |
| B. | DBH (cm) | 289.956 | 0.086 | 14.574 | 1 | 0.000 | 0.001 |
| C. | Height (m) | 280.053 | 0.104 | 24.477 | 1 | 0.000 | 0.078 |
| D. | Slenderness (m) | 304.504 | 0.000 | 0.026 | 1 | 0.872 | 0.265 |

^**^ Correlation is significant at the 0.01 level (2-tailed)

Climate zones are included on the first level of the Köppen classification. Initially, estimations were done with all 18 categories of the Köppen classification, however the results were not useful because many climate zones were hardly present in the data. The number of observations for each category should be larger than 10 or even as is mentioned in some studies 20 [5-8]. A total sample size lower than 500 can be sufficient if the aim of the analysis is to determine factors which are highly associated with an outcome [9]. Second the assumptions of the binary logistic regression were violated when for some categories perfect multicollinearity arose, which is a common problem with small sample sizes [10]. The values of the Wald-test for one of the climate zones (Tundra, zone 5) and for Slenderness are not significant, indicating that those factors do not contribute significantly to explaining tree failure.

Table 2: Parameter estimates of binary logistic regressions

| Ref. |  | Estimate | S.E. | Wald | Sig. | Log OR (*θ* ) | 95% C.I. Log OR | |
| --- | --- | --- | --- | --- | --- | --- | --- | --- |
| A. | Climate zones* |  |  |  |  |  |  |  |
|  | Zone 2 | -2.031 | 0.732 | 7.693 | 0.006 | 0.131 | 0.031 | 0.551 |
|  | Zone 3 | -0.896 | 0.386 | 5.395 | 0.020 | 0.408 | 0.192 | 0.869 |
|  | Zone 4 | -0.799 | 0.397 | 4.057 | 0.044 | 0.450 | 0.207 | 0.979 |
|  | Zone 5 | -0.827 | 1.450 | 0.325 | 0.596 | 0.437 | 0.026 | 7.504 |
|  | Constant | 0.827 | 0.320 | 6.656 | 0.010 | 2.286 |  |  |
|  |  |  |  |  |  |  |  |  |
| B. | DBH (cm) | 0.029 | 0.009 | 10.961 | 0.001 | 1.029 | 1.012 | 1.047 |
|  | Constant | -0.692 | 0.264 | 6.897 | 0.009 | 0.500 |  |  |
|  |  |  |  |  |  |  |  |  |
| C. | Height (m) | 0.098 | 0.022 | 20.102 | 0.000 | 1.104 | 1.057 | 1.152 |
|  | Constant | -1.380 | 0.351 | 15.488 | 0.000 | 0.252 |  |  |
|  |  |  |  |  |  |  |  |  |
| D. | Slenderness (m) | 0.001 | 0.004 | 0.026 | 0.872 | 1.001 | 0.993 | 1.009 |
|  | Constant | 0.051 | 0.281 | 0.033 | 0.855 | 1.053 |  |  |

* Zone 1 (tropical) is used as baseline.

All factors are included in one binary logistic regression, which explains previous contrasting results. In the full model the predictors Diameter Breast Height (DBH) and Slenderness are not significant see table 3. This outcome suggests that the factors Slenderness and DBH can therefore not be used worldwide, to predict tree failure.

Table 3: Binary logistic regression on the presence of failure in the study population

| Full model | -2 log likelihood | | Nagelkerke R² | | Model fit χ² | | df | | Sig. | | Sign. Hosmer & Lemeshow Test | |
| --- | --- | --- | --- | --- | --- | --- | --- | --- | --- | --- | --- | --- |
| Failure x Climate zones, DBH, Height, Slenderness | 268.218 | | 0.203 | | 36.312 | | 7 | | 0.000 | | 0.242 | |
|  | | | | | | | | | | | | |
|  | Estimate | S.E. | | Wald | | Sig. | | Log OR (*θ* ) | | 95% C.I. Log OR | | |
| Climate zones* |  |  | | 9.301 | | 0.054 | |  | |  | |  |
| Dry | -1.758 | 0.811 | | 4.698 | | 0.030 | | 5.797 | | 0.169 | | 3.347 |
| Temperate | -1.033 | 0.409 | | 6.377 | | 0.012 | | 2.818 | | 0.231 | | 1.835 |
| Continental | -0.675 | 0.438 | | 2.509 | | 0.123 | | 2.005 | | -0.183 | | 1.532 |
| DBH (cm) | 0.010 | 0.013 | | 0.898 | | 0.418 | | 0.988 | | -0.036 | | 0.015 |
| Height (m) | 0.089 | 0.032 | | 7.229 | | 0.005 | | 0.918 | | -0.152 | | -0.026 |
| Slenderness | -0.000 | 0.006 | | 0.031 | | 0.964 | | 0.999 | | -0.012 | | 0.013 |
| Constant | -0.761 | 0.578 | | 10.625 | | 0.188 | | 7.206 | | -0.371 | | 1.893 |

* Zone 1 (Tropical) is used as baseline.

The relative odds ratios are derived from the binary logistic regression output, indicating the odds that the factors mentioned appear in case of failure reported in the studies. It turns out that study populations from dry and temperate climate zones more often report tree failure than in a continental climate zone. However, relative odds do have some interpretation issues, since these are scaled by an arbitrary factor which is partly based on the unexplained variation of a binary outcome after inclusion of all explanatory variables [11].

An alternative figure to the log of odds are marginal effects that can be used to describe the impact of each factor on the presence of failure in the different study populations, see table 4. This means that for a 1 m increase in Height, the probability that the tree fails increases with 0.02 on a 0/1 scale.

Table 4: Marginal effects of the full model on failure

|  | dy/dx | S.E. | Z | P > \|z\| | X | 95% C.I. Log OR | |
| --- | --- | --- | --- | --- | --- | --- | --- |
| Climate zones |  |  |  |  |  |  |  |
| Dry | -0.378 | 0.123 | 3.07 | 0.002 | 0.055 | 0.137 | 0.620 |
| Temperate | -0.253 | 0.096 | 2.64 | 0.008 | 0.399 | 0.065 | 0.440 |
| Continental | -0.167 | 0.106 | 1.57 | 0.116 | 0.335 | -0.042 | 0.376 |
| DBH (cm) | 0.003 | 0.003 | -0.81 | 0.418 | 28.168 | -0.009 | 0.004 |
| Height (m) | 0.022 | 0.008 | -2.78 | 0.005 | 15.103 | -0.038 | -0.007 |
| Slenderness | -0.000 | 0.002 | 0.04 | 0.964 | 61.273 | -0.003 | 0.003 |

**References**

1. Field, A. (2017). Discovering statistics using IBM SPSS statistics: North American edition. sage.

2. Hozo, S. P., Djulbegovic, B., & Hozo, I. (2005). Estimating the mean and variance from the median, range, and the size of a sample. BMC medical research methodology, 5(1), 13.

3. IBM Corp. (2019). IBM SPSS statistics for windows. (Version 26.0). Armonk, NY: IBM Corp

4. StataCorp. (2019). Stata Statistical Software: Release 16. College Station, TX: StataCorp LLC.

5. Peduzzi P, Concato J, Kemper E, Holford TR, Feinstein AR. A simulation study of the number of events per variable in logistic regression analysis. J Clin Epidemiol. 1996;49(2):1373–1379. doi: 10.1016/S0895-4356(96)00236-3.

6. Concato J, Peduzzi P, Holford TR, Feinstein AR. The importance of event per variable (EPV) in proportional hazard analysis: I. Background, goals and general strategy. J Clin Epidemiol. 1995;48(12):1495–1501. doi: 10.1016/0895-4356(95)00510-2.

7. van Smeden Maarten, de Groot JAH, Moons KGM, Collins GS, Altman DG, Eijkemans MJC, et al. No rationale for 1 variable per 10 events criterion for binary logistic regression analysis. BMC Med Res Methodol. 2016;16:163. doi: 10.1186/s12874-016-0267-3.

8. Austin PC, Steyerberg EW. Events per variable (EPV) and the relative performance of different strategies for estimating the out-of-sample validity of logistic regression models. Stat Methods Med Res. 2017;26(2):796–808. doi: 10.1177/0962280214558972.

9. Bujang, M. A., Sa’at, N., & Bakar, T. M. I. T. A. (2018). Sample size guidelines for logistic regression from observational studies with large population: emphasis on the accuracy between statistics and parameters based on real life clinical data. The Malaysian journal of medical sciences: MJMS, 25(4), 122.

10. Midi, H., Sarkar, S. K., & Rana, S. (2010). Collinearity diagnostics of binary logistic regression model. Journal of Interdisciplinary Mathematics, 13(3), 253-267.

11. Norton, E. C., & Dowd, B. E. (2018). Log odds and the interpretation of logit models. Health services research, 53(2), 859-878.
